# Phylogenomics of the oxidative phosphorylation in fungi reveals extensive gene duplication followed by functional divergence

**DOI:** 10.1186/1471-2148-9-295

**Published:** 2009-12-21

**Authors:** Marina Marcet-Houben, Giuseppe Marceddu, Toni Gabaldón

**Affiliations:** 1 Comparative Genomics, Centre for Genomics Regulation, Dr. Aiguader, 88, 08003 Barcelona, Spain; 2 Dipartamento di Biologia Vegetale, Università Degli Studi di Torino, Viale P.A. Mattioly, 25, 10125 Torino, Italy

## Abstract

**Background:**

Oxidative phosphorylation is central to the energy metabolism of the cell. Due to adaptation to different life-styles and environments, fungal species have shaped their respiratory pathways in the course of evolution. To identify the main mechanisms behind the evolution of respiratory pathways, we conducted a phylogenomics survey of oxidative phosphorylation components in the genomes of sixty fungal species.

**Results:**

Besides clarifying orthology and paralogy relationships among respiratory proteins, our results reveal three parallel losses of the entire complex I, two of which are coupled to duplications in alternative dehydrogenases. Duplications in respiratory proteins have been common, affecting 76% of the protein families surveyed. We detect several instances of paralogs of genes coding for subunits of respiratory complexes that have been recruited to other multi-protein complexes inside and outside the mitochondrion, emphasizing the role of evolutionary tinkering.

**Conclusions:**

Processes of gene loss and gene duplication followed by functional divergence have been rampant in the evolution of fungal respiration. Overall, the core proteins of the respiratory pathways are conserved in most lineages, with major changes affecting the lineages of microsporidia, *Schizosaccaromyces *and *Saccharomyces/Kluyveromyces *due to adaptation to anaerobic life-styles. We did not observe specific adaptations of the respiratory metabolism common to all pathogenic species.

## Background

Oxidative phosphorylation (OXPHOS) is the primary energy-producing pathway in aerobic organisms [1]. It functions by coupling the energy obtained from the oxidation of certain metabolic substrates to the phosphorylation of adenosine biphosphate (ADP) to produce ATP. This is achieved by a process of electronic transference through an intricate assembly of more than 20 discrete carriers. These carriers are mainly grouped into four membrane-embedded protein complexes, named Complex I through Complex IV, which form the electron transport chain (ETC). Some of the complexes in this chain are able to use the energy liberated by the electron transfer to the pumping of protons across the membrane, thereby generating a proton gradient. Finally, the energy obtained from the dissipation of this gradient is used by a fifth protein complex, ATP-synthase or Complex V, to synthesize ATP.

In eukaryotes, the oxidative phosphorylation machinery resides in the inner membrane of the mitochondrion. Molecular phylogenies of eukaryotic OXPHOS components indicate that the core subunits of the complexes were inherited from the alpha-proteobacterial ancestor of mitochondria [2,3]. In contrast, other subunits might have different origins and show complex phylogenetic distributions [4]. Besides providing important information on how complex systems evolve, knowledge about lineage-specific variations may serve to identify novel components or interactions. For instance, the evolutionary analysis of Complex I across a set of eighteen eukaryotes, lead to the prediction that the so-far uncharacterised human protein B17L was involved in Complex I function [4]. This protein was later found to be participating as a chaperone in Complex I assembly and a mutation in this gene was identified in patients showing severe encephalopathy [5].

Fungi is the group of eukaryotic organisms that is best sampled in terms of fully sequenced genomes [6,7]. The adaptation of this kingdom to a diversity of environments is reflected in a high metabolic variability that also affects the respiratory pathway [3,8]. Indeed, the adaptation to oxygen-limited conditions or to high levels of oxidative stress during certain phases of their life cycle may have been crucial in the emergence of fermentative or pathogenic lifestyles. A recent comparative genomics study [9] has provided a comprehensive view of the patterns of presence and absence of OXPHOS components in 27 fungal species. Here we extend the analyses to 60 fully-sequenced fungal genomes and use a phylogenetics approach that enables us not only to obtain reliable orthology relationships but also to trace the history of duplications of OXPHOS components and related pathways during fungal evolution. In particular, we wanted to assess the role that gene duplication and functional divergence has played in the evolution of this pathway. A prediction of the gene-balance hypothesis is that independent duplications of protein complexes are likely to have deleterious effects [10], thereby constraining this mode of evolution in a pathway that is mostly composed of large complexes. Moreover, we wanted to test whether some loss or duplications of OXPHOS components could be associated to specific phenotypes such as virulence or adaptation to anaerobic environments. Altogether, our results show a relatively high rate of duplication events that affect 76% of the protein families surveyed. Interestingly, some of these duplications have been directly followed by processes of functional divergence, sometimes involving the recruitment of one of the duplicates to other multi-protein complexes.

## Results and Discussion

### Phylogenomic profiling of the OXPHOS pathway

**Figure 1:**
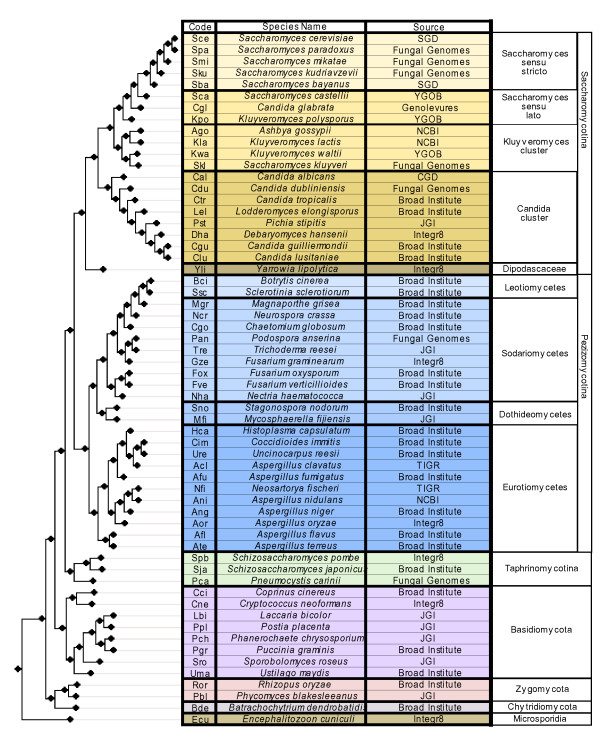
**Species table**. List of species and their corresponding three-letter codes used in the analysis. The tree on the left represents the fungal species tree as described by a recent analysis [7]. The names of the major fungal taxa, as provided by the source database, are indicated to the right of the tree. A list of synonyms for this species names is provided in the additional file 1. Sources of the sequences are: JGI http://www.jgi.doe.gov, Broad Institute http://broad.mit.edu, YGOB http://wolfe.gen.tcd.ie/ygob, SGD http://www.yeastgenome.org, Fungal Genomes http://fungalgenomes.org, Genolevures http://www.genolevures.org/, integr8 http://www.ebi.ac.uk/integr8, Candida genome database http://www.candidagenome.org, NCBI http://www.ncbi.nlm.nih.gov.

**Figure 2:**
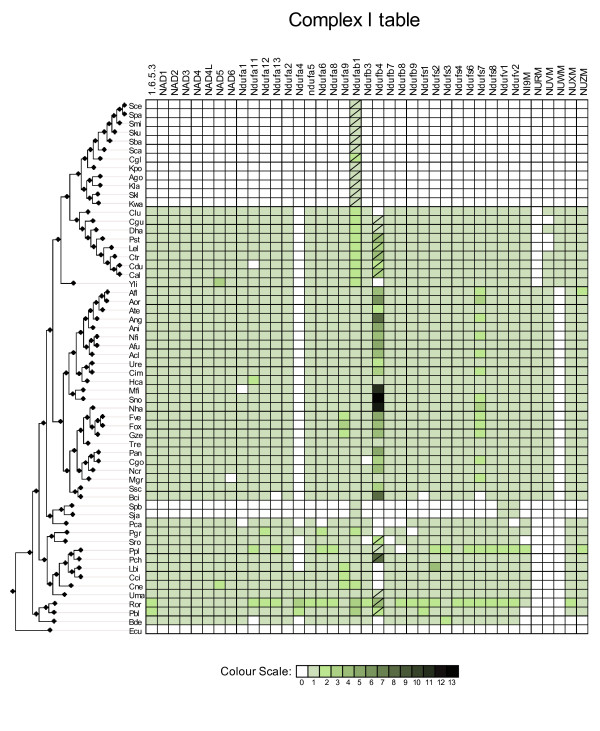
**Complex I**. Phylogenetic distribution across 60 fungal species of Complex I subunits. Absences of a corresponding ortholog in a given species is indicated with a blank square or a crossed green square. Crossed green squares indicate that no ortholog was found but at least one paralog is present. Presence of orthologs is indicated with uncrossed green squares. The different colour intensities correspond to the number of homologs of the query protein found in that specific genome. The species are ordered according to their phylogenetic position in the fungal species tree [7].

**Figure 3:**
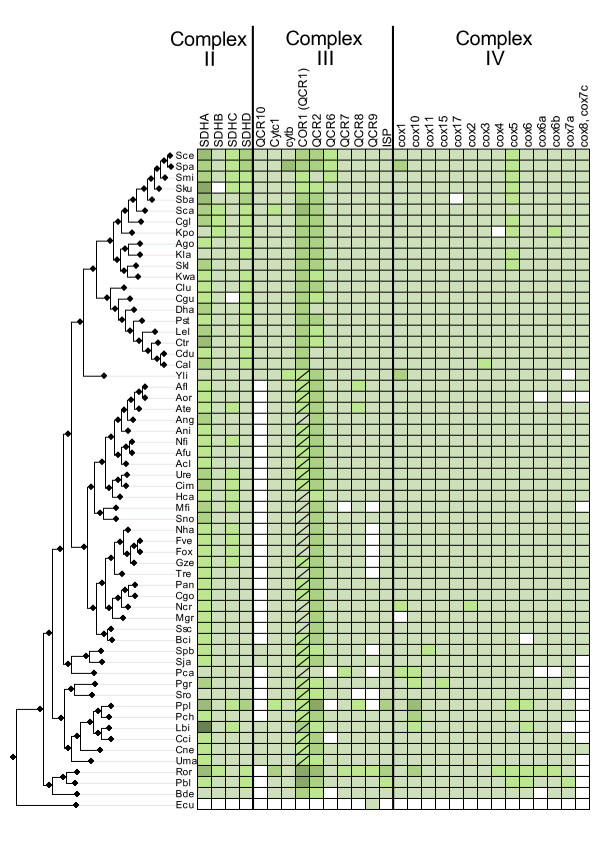
**Complex II-III-IV**. Phylogenetic distribution across 60 fungal species of subunits from Complexes II, III and IV subunits. Symbols and codes as in figure 2.

**Figure 4:**
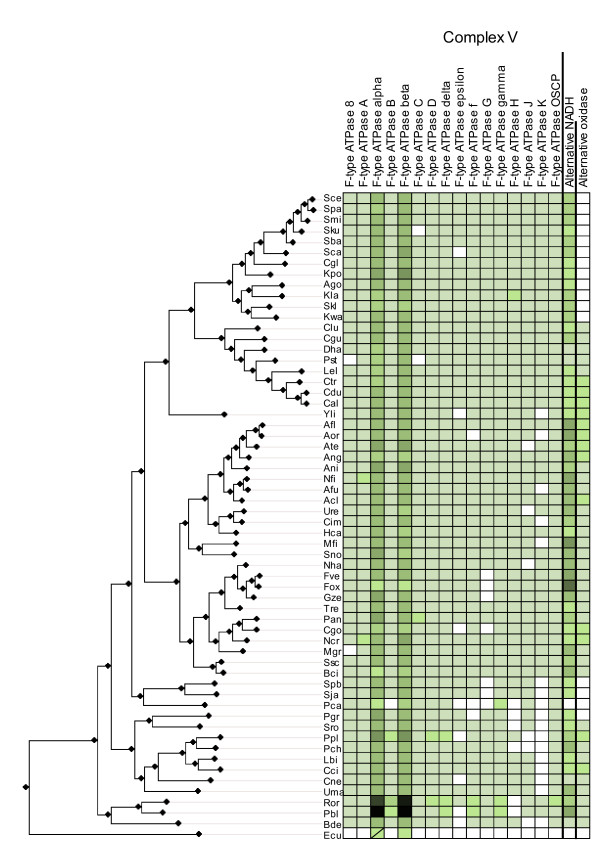
**Complex V-Alternative oxidases and dehydrogenases**. Phylogenetic distribution across 60 fungal species of Complex V and alternative oxidases and dehydrogenases. Symbols and codes as in figure 2.

Sequences of fungal proteins annotated as OXPHOS components were retrieved from the KEGG database [11] and used as queries for blastp searches against the proteins encoded in 60 fully-sequenced fungal genomes (see figure 1 and Methods section). A phylogenetic analysis was performed on each set of homologous proteins to derive a phylogenetic tree. This tree was used to establish orthology and paralogy relationships using a species-overlap algorithm that has been described earlier [12]. This phylogeny-based approach to orthology detection, approaches more closely the original definition of orthology and reflects more appropriately the complex evolutionary relationships within protein families [13,14]. The presence of the different components of the respiratory pathway in the species surveyed is summarized in figures 2, 3 and 4. Overall, our results agree with those reported by Lavin et. al in the 27 species that both surveys have in common [9]. That the two approaches render so similar results, indicates that, despite using different approaches both methods have a similar stringency in the detection of OXPHOS components in this taxonomic range. In addition, our study extends the information on the distribution of components of the respiratory pathway to 33 additional species. The main advantage of our approach, however, is more qualitative than quantitative. By performing phylogenetic analyses on every protein family, we can readily obtain information on duplication events affecting components of the respiratory pathway, an important evolutionary process that was ignored in the previous study. Recognizing gene duplications is important, since this process is considered one of the main processes that drive functional innovation [15]. Our study reveals that duplication events have affected the OXPHOS pathway extensively. Overall, we detect duplications in 76% of the families surveyed. These results were similar (duplications in 75% of the families surveyed), when more stringent cut-offs for homology detection were applied (see figures S1, S2 and S3 in the additional file 1). Such high proportion of duplications is not the result of errors in the annotation or assembly of the genomes. We controlled for this by inspecting manually every duplication case to discard dubious cases. Moreover, even when species-specific duplications in which duplicates had more than 95% identity at the nucleotide level or all duplications from the recently assembled genomes *Postia placenta *and *Puccina graminis *were not taken into account, the fraction of OXPHOS families with a duplication event remained high (71% and 74%, respectively). Zygomycota, in particular, present the highest proportion of duplicated proteins in the OXPHOS pathway. For instance, we found duplications in 60% of the genes involved in *Rhizopus oryzae *OXPHOS pathway. A large percentage of these duplications (82%) can be mapped specifically to the *R. oryzae *lineage or to the lineage preceding the separation of *R. oryzae *and *Phycomyces blakesleeanus *and thus are specific of Zygomycota species. This large amount of lineage specific duplications seems to be general in *R. oryzae *and *P. blakesleeanus *(unpublished observation from our group). An interesting possibility is that the ancestors of these organisms underwent a Whole Genome Duplication (WGD) event, similar to that described for *Saccharomyces *[16]. This possibility has recently been confirmed for *R. oryzae *[17], in a comprehensive study that catalogues duplicated regions where the gene order is conserved. Consistently with our results, a duplication of nearly all subunits of the protein complexes associated with respiratory electron transport chains is detected, although our phylogeny-based approach detects additional, more ancestral, duplications that are not associated to the WGD event.

### Complete loss of the OXPHOS pathway in microsporidia and two additional independent losses of Complex I coupled with alternative dehydrogenase expansions in Schizosaccharomyces and Saccharomycetales

**Figure 5:**
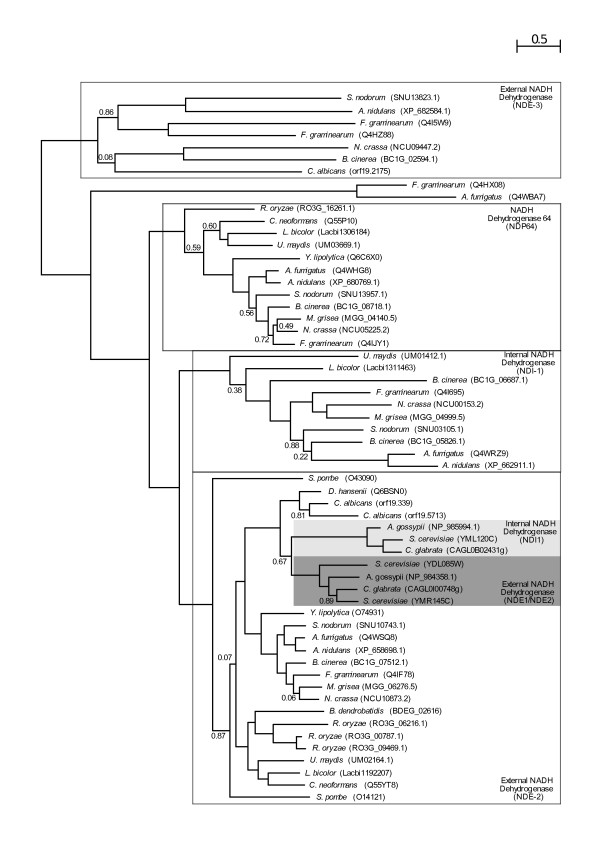
**Phylogenetic tree representing the evolution of the alternative dehydrogenase protein family**. The model used was WAG and approximate Likelihood (aLRT) support of the tree partitions is indicated if lower than 0.9. Duplications involving *S. cerevisiae *were marked with coloured boxes, while those involving *N. crassa *are indicated with white boxes. The species name is followed by the protein name according to the database from which the sequences where retrieved. Functional annotations were taken from Saccharomyces Genome Database (*S. cerevisiae*) [39] and the Broad Institute (*N. crassa*). This tree represents a subset of the sequences used in the analysis, the tree with the full set of sequences can be accessed in the additional file 1.

Our results confirm earlier findings of a complete loss of the OXPHOS pathway in microsporidia [18] and the absence of most components of Complex I in *Schizosaccharomyces *and Saccharomycetales [4]. We are able to find most of the subunits of complex I in the Taphrinomycotina species *Pneumocystis carinii*, suggesting that the event of gene loss occurred after the diversification of *Pneumocystis *and *Schizosaccharomyces lineages*. The apparent multiple absences of Complex I subunits, and those of other complexes, in *P. carinii *is probably related to a low coverage of the genome sequence for this organism. Similarly, the presence of a complete repertoire of Complex I subunits in all species in the *Candida *cluster and the lack of this complex in all surveyed species from the *Saccharomyces/Kluyveromyces *clade, situates the loss of Complex I in the latter lineage. Remarkably, the two independent losses of Complex I in the Taphrinomycotina and *Saccharomyces/Kluyveromyces *clades are concomitant with independent expansions of their alternative NADH dehydrogenases repertoire by virtue of gene duplications. Alternative NADH dehydrogenases bypass Complex I electron transport, oxidizing NADH without pumping of protons. The duplication of alternative NADH dehydrogenases (Figure 5) might have provided a selective advantage for yeast species using predominantly fermentative metabolism, due to adaptation to anaerobic environments. Excess of NADH causes a problem under fermentative anaerobic growth, since it prevents further oxidation of substrates due to a lack of a sufficient NAD+ pool to accept electrons. Thus, the diversification of pathways to further oxidize NADH would have been beneficial in such conditions. The loss of Complex I in the same evolutionary periods might also be related to adaptation to fermentative growth. It is unclear which of the processes preceded the other or whether both processes were concomitant. A higher taxon sampling within the Saccharomycotina and Taphrinomycotina might help to solve this issue in the future. Also coupled with Complex I loss, and in line with adaptations to anaerobic environments in the abovementioned lineages, we observe the loss of alternative oxidases.

### Duplications in Alternative Oxidases are not necessarily coupled with a pathogenic life-style

Alternative oxidases catalyze the cyanide-resistant alternative pathway of mitochondrial respiration in some fungi, plants and several protists. This pathway directly transfers electrons from the ubiquinone pool to oxygen, thereby bypassing complex III and cytochrome c oxidase [19]. Alternative oxidases are common in yeasts but limited almost exclusively to non-fermentative and crabtree-negative yeasts. Alternative oxidases participate in energy production but also in antioxidant defense of cells. It has been shown that alternative oxidases represent an important factor for the survival of pathogenic fungi inside macrophages [20]. Considering this, it could be postulated that the duplication of these enzymes might have played a role in the emergence of pathogenesis in several mammal fungal pathogens. In our survey we detect several copies of alternative oxidases in 13 species. Some of these duplications seem to have occurred quite recently in their respective lineages, such as the duplication that lead to AOX1 and AOX2 (orf19.4774 and orf19.4773 in *C. albicans*) involving some *Candida s*pecies, which can be mapped before the speciation *of C. tropicalis, C. dubliniensis *and *C. albicans*. Although many of these duplications do affect pathogenic genera such as *Candida *and *Aspergillus*, there are notable exceptions such as the intra-specific duplications found in the generally non-pathogenic species *Yarrowia lypolytica *or *Coprinus cinereus*. Conversely, we find pathogenic species such as *Histoplasma capsulatum *or *Cryptococcus neoformans *that have been shown to survive in macrophages [21] and nevertheless present a single alternative oxidase. Taken all together, our results suggest that a single copy of alternative oxidase gene is sufficient to protect fungal pathogens against macrophages and rather points to alternative selective advantages for the duplication of this gene. Conversely, alternative adaptations might be behind the emergence of the ability to survive inside macrophages in certain lineages. For instance, the presence of a polysaccharide capsule in Cryptococcus has been shown to confer resistance to oxidative stress [22].

### Extensive duplication followed by functional divergence in the fungal OXPHOS pathway

According to the gene balance hypothesis [10], the duplication of genes that encode for subunits of multi-protein complexes should have a higher chance of being deleterious due to dosage effects. As a result, one would expect to find few duplication events in the OXPHOS system, as this is mainly formed by intricate complexes. Contrary to that expectation, we find numerous cases of duplications in OXPHOS proteins, which overall affect 66 (76%) of the proteins surveyed. These have occurred at different moments in fungal evolution. At least for the genes duplicated during the Whole Genome Duplication event (WGD) occurred in the yeast lineage about 80 Myr ago, the rate of gene loss of duplicated OXPHOS genes is not higher than the overall rate for *Saccharomyces cerevisiae*. Indeed, our study finds six yeast proteins (all complex II subunits, Qcr6p and Cox5p), whose duplication is mapped to the WGD event. These represent 7% of the OXPHOS proteins, meaning that for 93% of the nuclear OXPHOS proteins supposedly duplicated in WGD were subsequently lost, a rate of gene loss that is roughly similar to the 88% estimated for the whole *S. cerevisiae *genome [16]. It must be noted that one of the duplications affected the whole Complex II, meaning that the duplication was conservative in terms of stoichiometry of the different subunits. However, one of the duplicated subunits has been subsequently lost in the *Saccharomyces sensu stricto *species, suggesting the four duplicates do not form an alternative complex II. A possible reconciliation between the extensive rate of gene duplications and the gene balance hypothesis is that functional divergence directly followed the duplication event, thereby facilitating the retention of both duplicates [23]. Differences in the expression patterns of some of the WGD duplicates, point to a functional specialization of each duplicate. For instance, Cox5 (YNL052W) is expressed during aerobic growth whereas its paralog (YIL111W) is expressed under anaerobic growth [24]. Similarly, the duplicate of the SDHA complex II subunit (YJL045W) is specifically expressed during the diauxic shift. Several other observations suggest that functional divergence processes have been common after duplication of OXPHOS protein families (see below).

### Evolutionary cross-talk between the OXPHOS complexes and other multi-protein complexes

Several instances of paralogy relationships between complex I subunits and other mitochondrial multi-protein complexes have been previously reported [4]. This is the case for the NDUFA11 subunit, which is paralogous to the Tim17/22 family as well as that of NI8M (NDUFA2) and NUZM, which are paralogous to L43 and L2 subunits of the mitochondrial ribosome. It has been suggested that OXPHOS proteins with paralogs in other complexes would play a structural role rather than being involved in proton or electron transport, since ribosomes and the import machinery do not display those functions [4]. Similarly, we find several instances of paralogs of OXPHOS subunits that play a role in other complexes. Interestingly, another evolutionary connection between OXPHOS and the mitochondrial import machinery (MIM) is evidenced by the fact that the MIM subunit TIM18 (YOR297C) is a paralog of the Complex II subunit SDHD (YDR178W). Yet another paralog of the same Complex II subunit, which originated from a more recent duplication in the Saccharomycotina lineage (YLR164W) encodes for a mitochondrial inner membrane protein of yet unknown function. Paralogies to the protein import system in the mitochondrion extend to the two subunits of the Mitochondrial processing peptidase (MPP), an essential processing enzyme that cleaves the N-terminal targeting sequences from mitochondrially imported proteins [25]. Indeed the large and small subunits MAS1 (YLR163C) and MAS2 (YHR024C) are homologous to QCR1 (YBL045C) and QCR2 (YPR191W) subunits of Complex III. Paralogy relationships to other multi-protein complexes extend beyond mitochondria. Indeed, several paralogs of Complex V subunits have been described as components of complexes from other cell compartments. For instance, the alpha and beta subunits of the F1 sector of the mitochondrial ATP synthase (YBL099W, YJR121W) are paralogous to the A and B subunits of the vacuolar ATP synthase (YDL185W, YBR127C). Vacuolar ATP synthases are found in the membranes of a large number of organelles which include endosomes, lysosomes and secretory vesicles. This duplication, however is not specific to fungi, since both paralogous groups have representatives in *Arabidopsis thaliana *and *Homo sapiens *(see phylogenetic trees additional file 1: figure S4), which indicates that the duplication preceded the diversification of plants and opisthokonts.

### Yeast ACPM is possibly not a complex I remnant but a Saccharomycotina-specific paralog of complex I Acyl-carrier protein

**Figure 6:**
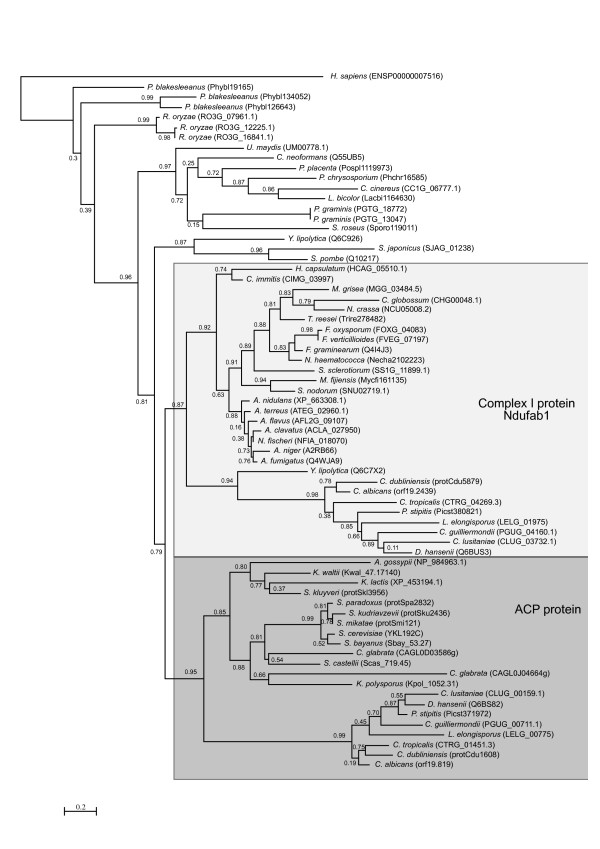
**Phylogenetic tree representing the evolution of the ACPM protein family**. The model used was WAG and approximate Likelihood (aLRT) support of the tree partitions is indicated. This tree represents a subset of the sequences used in the analysis, the tree with the full set of sequences can be accessed in the additional file 1.

Although previously identified as a remnant Complex I subunit in the Complex I devoid organism *S. cerevisiae *[4], our current phylogenomic analysis suggest that this protein might actually be a paralog originated from an ancient duplication that is specifically conserved in Saccharomycotina (Figure 6). This paralogy relationship is supported by approximate Likelihood Ratio Tests (aLRT) analyses of the duplication node (0.79, shown in the figure) as well as by bootstrap analyses (74% bootstrap support, not shown in the figure). This finding clarifies apparent inconsistencies in the function of Complex I acyl-carrier protein and the isolated ACPM protein (YKL192C). Indeed *S. cerevisiae *ACPM protein has been found to participate in the synthesis of octanoic acid, a precursor of lipoic acid [26], whereas the acyl carrier Complex I subunit in *Neurospora crassa *seems not to participate in this process [27]. The fact that a mammalian homolog of this protein family has also been identified as a Complex I subunit in bovine mitochondria [28], suggests that association with Complex I is an ancestral feature of the family. Taken together, these results indicate that a process of functional divergence might have occurred after the duplication event diverting the new duplicate for specializing in the synthesis of octanoid acid. This specialization is presumably present in all Saccharomycotina species including the Candida group, which additionally possesses the true acyl carrier Complex I subunit.

## Conclusions

Altogether our results shed light on how processes of gene loss, duplication and functional divergence have shaped the core of the respiratory pathway in fungi. Although most fungal organisms present a similar overall composition in terms of respiratory complexes, extensive differences in what particular units have been lost or duplicated in each complex, might help explaining differences found at the physiological level. This continuous evolution of OXPHOS components seems to be common in other groups of organisms [4,29,30], emphasizing the plasticity of this central energetic pathway.

## Methods

### Sequence data

Proteins encoded in 60 fully-sequenced fungal genomes were downloaded from several databases (figure 1). For consistency, we used in our analysis the species names as provided by the database source. Some of these species have been renamed and the corresponding new names and synonyms are listed in the additional file 1 (Additional table S1). Additionally, genomes from *Homo sapiens *and *Arabidopsis thaliana *were downloaded from ensembl http://www.ensembl.org. The final database comprises 626,834 unique protein sequences.

### Reconstruction of the presence/absence matrix

Fungal proteins annotated as being part of the OXPHOS pathway were downloaded from the KEGG database (map 00190) [31]. In addition, 6 proteins that were identified in the literature as belonging to complex I but were not present in the KEGG database were downloaded from UniProt and included in the analyses (NI9M, NURM, NUWM, NUXM and NUZM). The resulting 85 proteins were used to perform a blast search against a database of fungal proteins encoded in 60 fungal genomes (figure 1). Low complexity filters were used in the blast search. To detect homology, we used the same parameters that have been used previously in the same taxonomic range [7]. In brief, only significant hits (E-val < 10^-3^) that aligned with a continuous region covering more than one third of the query sequence were selected. Note that the use of low complexity filters in the blast can reduce significantly the length of continuous regions of homology. Sets of homologous sequences were aligned and used to reconstruct a Maximum Likelihood tree from which orthology relationships were inferred (see below). These orthology relationships were used to build a presence/absence matrix (figures 2, 3 and 4) in which for each OXPHOS component the species with a corresponding ortholog are indicated. Putative absences in the matrix were double-checked by tBlastN [32] searches against the corresponding genome sequence and Blast searches from family members of more related species. These hits were checked manually and whenever they were considered orthologous to the already identified members they were added to the list.

### Phylogenetic analyses

We used a similar pipeline to that described in [33]. Sets of homologous proteins were aligned using MUSCLE 3.6 [34] with default parameters. Positions in the alignment with gaps in more than 10% of the sequences were trimmed with trimAl [35]. Finally, PhyML aLRT version [36,37] was used to derive Maximum Likelihood (ML) trees. Four different evolutionary models were used for each seed sequence (JTT, WAG, Blosum62 and VT). In all cases, a discrete gamma-distribution model with four rate categories plus invariant positions was used, estimating the gamma parameter and the fraction of invariant positions from the data. The evolutionary model best fitting the data was determined by comparing the likelihood of the used models according to the AIC criterion [38]. Orthology and paralogy relationships among members of a family were inferred from the analysis of their corresponding phylogenetic trees, using a previously described algorithm that has been described before and has been shown to be accurate [7,12]. Phylogeny-based methods are considered to better reflect the actual complexity of orthology relationships than pair-wise methods such as best-bidirectional hits [14]. All phylogenetic trees are provided in the additional file 1: figure S4 as well as a list of proteins used as a seed in our analyses (Additional file 1: table S2). All duplications where manually checked to discard possible cases of spurious duplications. This was done by manually inspecting the alignments and the nucleotide sequences of the relevant duplicates. Moreover, the corresponding genome browsers or assembly data were searched to analyze the sequence context of the duplicates. Highly similar sequences in which one is only partially sequenced or in a small contig can be taken as possible source of errors. These cases were discarded. Total counts of duplications were also computed discarding the fraction of duplications that is expected to be more sensitive to error annotation: lineage-specific duplications with highly similar duplicates and duplications found in recently assembled genomes (see main text).

## Authors' contributions

TG conceived of the study and coordinated the analyses. MMH and GM performed the analyses. TG and MMH wrote the manuscript. All authors read and approved the manuscript.

## Supplementary Material

Additional file 1 **Additional Material**. Additional figures and tables cited in the text. Click here for file
