# Supplementary material for: Phylogenomics of the oxidative phosphorylation in fungi reveals extensive gene duplication followed by functional divergence
Source: BMC Evol Biol. 2009 Dec 21;9:295. doi: 10.1186/1471-2148-9-295 (PMC2803194; doi:10.1186/1471-2148-9-295)

Additional files for the paper:

Marina Marcet-Houben Giussepe Marceddu and Toni Gabaldón:
"Phylogenomics of the oxidative phosphorylation in fungi reveals extensive gene duplication followed by functional divergence."

Index of contents:

1.- Additional files Legends

2.- Additional files

1.- Additional files legends

Additional table 1

Table of species names as supplied by taxonomy (http://www.ncbi.nlm.gov/sites/entrez?db=taxonomy). The first column represents the names taken from the source proteome databases. The remaining columns supply the different sysnonym names each species can have taken from Taxonomy.

Additional table 2

Table depicting the locus tag for the seed sequence used in each protein.

Additional table 3

List of protein accession numbers according to the source database from which they were downloaded (see Figure 1). The first column represents the OXPHOS subunit in which the protein was detected, the second corresponds to the code of the species and the third is the list of paralogs separated by "||".

Additional figure 1

Phylogenetic distribution across 60 fungal species of Complex I subunits using strict blast filters (e-value: 1e-04 and a continuous overlap 0.4). Symbols and codes as in figure 2.

Additional figure 2

Phylogenetic distribution across 60 fungal species of subunits from Complexes II, III and IV subunits using strict blast filters (e-value: 1e-04 and a continuous overlap 0.4). Symbols and codes as in figure 2.

Additional figure 3

Phylogenetic distribution across 60 fungal species of Complex V and alternative oxidases and dehydrogenases using strict blast filters (e-value: 1e-04 and a continuous overlap 0.4). Symbols and codes as in figure 2.

Additional figure 4

Trees corresponding to each protein in the fungal Oxidative phosphorilation pathway. Trees were done using a maximum Likelihood approach. Four different evolutionary models were used and the

best one was included in this file. Evolutionary events are indicated at each node. Red nodes indicate speciation events while blue nodes represent duplication events.

2.- Additional files

Additional table 1

Names from source proteome databases	Data taken from Taxonomy (http://www.ncbi.nlm.nih.gov/sites/entrez?db=taxonomy)		
	Species name	Synonym	Anamorph
Ashbya gossypii	Ashbya gossypii ATCC 10895	Eremothecium gossypii ATCC 10895	
Aspergillus clavatus	Aspergillus clavatus		
Aspergillus flavus	Aspergillus flavus		
Aspergillus fumigatus	Aspergillus fumigatus		
Aspergillus nidulans	Emericella nidulans	Aspergillus nidulellus	Aspergillus nidulans
Aspergillus niger	Aspergillus niger	Aspergillus niger Tiegh.	
Aspergillus oryzae	Aspergillus oryzae		
Aspergillus terreus	Aspergillus terreus		
Batrachochytrium dendrobatis	Batrachochytrium dendrobatidis		
Botrytis cinerea	Botrytis fuckeliana		
Candida albicans	Candida albicans	Candida stellatoidea, Candida stellatoidea type I	
Candida dubliniensis	Candida dubliniensis		
Candida glabrata	Candida glabrata	Torulopsis glabrata	
Candida guilliermondii	Pichia guilliermondii	Candida guilliermondii var. carpophila, Yamadazyma guilliermondii	Candida guilliermondii
Candida lusitaniae	Clavispora lusitaniae	Candida lusitaniae	
Candida tropicalis	Candida tropicalis		
Chaetomium globosum	Chaetomium globosum		
Coccidioides immitis	Coccidioides immitis		
Coprinopsis cinerea	Coprinopsis cinerea	Coprinus cinereus, Coprinus macrorhizus	
Cryptococcus neoformans	Filobasidiella neoformans		Cryptococcus neoformans
Debaryomyces hansenii	Debaryomyces hansenii	Torulaspora hansenii	
Encephalitozoon cuniculi	Encephalitozoon cuniculi		
Fusarium graminearum	Gibberella zeae		Fusarium graminearum
Fusarium oxysporum	Fusarium oxysporum		
Fusarium verticillioides	Gibberella moniliformis	Fusarium moniliforme	Fusarium verticillioides
Histoplasma capsulatum	Ajellomyces capsulatus	Ajellomyces capsulata	Histoplasma capsulatum
Kluyveromyces lactis	Kluyveromyces lactis	Kluyveromyces drosophilarum, Kluyveromyces lactis var. drosophilarum, Kluyveromyces lactis var. lactis, Kluyveromyces marxianus lactis, Kluyveromyces marxianus var. drosophilarum, Kluyveromyces marxianus var. lactis	Candida sphaerica
Kluyveromyces polysporus	Vanderwaltozyma polyspora	Kluyveromyces polysporus, Vanderwaltozyma sp. SA20S10	
Kluyveromyces waltii	Lachancea waltii	Kluyveromyces waltii, Zygofabospora waltii	
Laccaria bicolor	Laccaria bicolor		
Lodderomyces elongisporus	Lodderomyces elongisporus		
Magnaporthe grisea	Magnaporthe grisea	Magnaporthe grisea (T.T. Hebert) M.E. Barr, Pyricularia grisea (Cooke) Sacc.	Pyricularia grisea
Mycosphaerella fijiensis	Mycosphaerella fijiensis	Mycosphaerella fijiensis M. Morelet	Paracercospora fijiensis var. difformis
Nectria haematococca	Nectria haematococca	Haematonectria haematococca, Nectria haematococca var. brevicona	
Neosartorya fischeri	Neosartorya fischeri	Aspergillus fischeri, Neosartorya fischeri var. fischeri	Aspergillus fischerianus
Neurospora crassa	Neurospora crassa		
Phanerochaete chrysosporium	Phanerochaete chrysosporium	Chrysosporium pruinosum (Gilman et Abbott) Carmich., Phanerochaete chrysosporium Burdsall, Sporotrichum pruinosum Gilman et Abbott	Sporotrichum pruinosum
Phycomyces blakesleanus	Phycomyces blakesleeanus		
Pichia stipitis	Pichia stipitis	Yamadazyma stipitis	
Pneumocystis carnii	Pneumocystis carinii	Pneumocystis carinii f. sp. carinii, Pneumocystis carinii f. sp. ratti	
Podospora anserina	Podospora anserina		
Postia placenta	Postia placenta		
Puccinia graminis	Puccinia graminis		
Rhizopus oryzae	Rhizopus oryzae	Rhizopus arrhizus, Rhizopus arrhizus var. arrhizus, Rhizopus chiuniang, Rhizopus delemar, Rhizopus formosaensis, Rhizopus formosaensis var. chlamydosporus, Rhizopus hangchow, Rhizopus javanicus Y. Takeda, Rhizopus liquefaciens, Rhizopus peka, Rhizopus pseudochinensis, Rhizopus suinus, Rhizopus tamarii, Rhizopus thermosus, Rhizopus tritici	
Saccharomyces cerevisiae	Saccharomyces cerevisiae	Saccharomyces capensis, Saccharomyces italicus, Saccharomyces oviformis, Saccharomyces uvarum var. melibiosus	Candida robusta
Saccharomyces bayanus	Saccharomyces bayanus		
Saccharomyces castellii	Naumovia castellii	Saccharomyces castellii, Saccharomyces castellii Capriotti	
Saccharomyces kluyveri	Lachancea kluyveri	Saccharomyces kluyveri	
Saccharomyces kudriavzevii	Saccharomyces kudriavzevii		
Saccharomyces mikatae	Saccharomyces mikatae		
Saccharomyces paradoxus	Saccharomyces paradoxus		
Schizosaccharomyces japonicus	Schizosaccharomyces japonicus	Schizosaccharomyces japonicus var. japonicus, Schizosaccharomyces japonicus var. versatilis	
Schizosaccharomyces pombe	Schizosaccharomyces pombe	Schizosaccharomyces malidevorans	
Sclerotinia sclerotiorum	Sclerotinia sclerotiorum		
Sporobolomyces roseus	Sporobolomyces roseus		
Stagonospora nodorum	Phaeosphaeria nodorum	Leptosphaeria nodorum	Septoria nodorum, Stagonospora nodorum
Trichoderma reesei	Hypocrea jecorina		Trichoderma reesei
Uncinocarpus reesii	Uncinocarpus reesii		
Ustilago maydis	Ustilago maydis		
Yarrowia lipolytica	Yarrowia lipolytica	Mycotorula lipolytica	Candida lipolytica

Additional table 2

	KEGG annotation (map00190)	Locus tag of seed sequence
Complex I	1.6.5.3	NCU03093
	NAD1	CaalfMp03
	NAD2	CaalfMp09
	NAD3	CaalfMp10
	NAD4	CaalfMp14
	NAD4L	CaalfMp12
	NAD5	CaalfMp13
	NAD6	UsmafMp16
	Ndufa1	NCU04781
	Ndufa11	NCU02280
	Ndufa12	NCU01142
	Ndufa13	NCU09299
	Ndufa2	NCU03156
	Ndufa4	UM02800.1
	ndufa5	NCU05299
	Ndufa6	NCU00418
	Ndufa8	NCU02472
	Ndufa9	NCU02373
	Ndufab1	NCU05008
	Ndufb3	NCU09002
	Ndufb4	NCU07736
	Ndufb7	orf19.446.2
	Ndufb8	NCU09460
	Ndufb9	orf19.5547
	Ndufs1	NCU01765
	Ndufs2	NCU02534
	Ndufs3	NCU04074
	Ndufs4	NCU05221
	Ndufs6	NCU00484
	Ndufs7	NCU03953
	Ndufs8	NCU05009
	Ndufv1	NCU04044
	Ndufv2	NCU01169
	NI9M	NCU00670
	NURM	NCU00969
	NUVM	NCU00160
	NUWM	orf19.1549
	NUXM	NCU01859
	NUZM	NCU08930
Alternative NADH	1.6.5.3 / 1.6.99.3	NCU10873
Alternative oxidase	AOX	NCU07953
Complex II	SDHA	NCU08336
	SDHB	orf19.637
	SDHC	YKL141W
	SDHD	NCU03031
Complex III	QCR10	orf19.2439.1
	Cytc1	YOR065W
	cytb	Q0105
	COR1 (QCR1)	YBL045C
	QCR2	YPR191W
	QCR6	YFR034C
	QCR7	NCU08940
	QCR8	YJL166W
	QCR9	YGR183C
	ISP	YEL024W
Complex IV	cox1	Q0045
	cox10	YPL172C
	cox11 (assembly protein)	orf19.1416
	cox15 (assembly protein)	NCU04817.2
	cox17 (assembly protein)	YLL009C
	cox2	Q0250
	cox3	Q0275
	cox4	YGL187C
	cox5	YIL111W
	cox6	YHR051W
	cox6a	YGL191W
	cox6b	YLR038C
	cox7a	YDL067C
	cox8, cox7c	YLR395C
Complex V	F-type ATPase (Eukaryotes) 8	Q0080
	F-type ATPase (Eukaryotes) A	Q0085
	F-type ATPase (Eukaryotes) alpha	YBL099W
	F-type ATPase (Eukaryotes) B	YPL078C
	F-type ATPase (Eukaryotes) beta	YJR121W
	F-type ATPase (Eukaryotes) C (ATP9)	Q0130
	F-type ATPase (Eukaryotes) D	YLR123C
	F-type ATPase (Eukaryotes) delta	YDL004W
	F-type ATPase (Eukaryotes) epsilon	YPL271W
	F-type ATPase (Eukaryotes) f	YDR377W
	F-type ATPase (Eukaryotes) G	YHR039C-A
	F-type ATPase (Eukaryotes) gamma	NCU09119
	F-type ATPase (Eukaryotes) H	YLR295C
	F-type ATPase (Eukaryotes) J	YML081C-A
	F-type ATPase (Eukaryotes) K	YOL077W-A
	F-type ATPase (Eukaryotes) OSCP	YDR298C

Additional table 3

Alternative NADH	Cgo	CHG10832.1 || CHG03788.1 || CHG02921.1 || CHG03027.1
Alternative NADH	Ure	UREG_07032.1 || UREG_00726.1 || UREG_03337.1
Alternative NADH	Uma	UM01412.1 || UM03669.1 || UM02164.1
Alternative NADH	Ctr	CTRG_02605.3 || CTRG_02112.3
Alternative NADH	Spa	protSpa21 || protSpa759 || protSpa4515
Alternative NADH	Gze	Q4HX08 || Q4I5W9 || Q4HZ88 || Q4I695 || Q4IJY1 || Q4IF78
Alternative NADH	Ror	RO3G_16261.1 || RO3G_06216.1 || RO3G_09469.1 || RO3G_00787.1
Alternative NADH	Kwa	Kwal_0.366 || Kwal_33.14300 || Kwal_34.15827
Alternative NADH	Afl	AFL2G_08112 || AFL2G_00016 || AFL2G_05143 || AFL2G_09295 || AFL2G_07578
Alternative NADH	Ago	NP_985994.1 || NP_984358.1
Alternative NADH	Bci	BC1G_02594.1 || BC1G_06687.1 || BC1G_08718.1 || BC1G_05826.1 || BC1G_07512.1
Alternative NADH	Sro	Sporo111927 || Sporo117808 || Sporo110322
Alternative NADH	Aor	Q2U6V8 || Q2UPG2 || Q2U0P2 || Q2UMR1 || Q2UNI0
Alternative NADH	Tre	Trire260352 || Trire2120272 || Trire267588 || Trire258815
Alternative NADH	Hca	HCAG_02304.1 || HCAG_00968.1 || HCAG_01305.1
Alternative NADH	Nfi	NFIA_095160 || NFIA_010910 || NFIA_082190 || NFIA_013570
Alternative NADH	Pch	Phchr13743 || Phchr1134180 || Phchr1123031 || Phchr16157
Alternative NADH	Afu	Q4WBA7 || Q4WRZ9 || Q4WHG8 || Q4WSQ8
Alternative NADH	Lel	LELG_02069 || LELG_00216
Alternative NADH	Ssc	SS1G_05812.1 || SS1G_01797.1 || SS1G_13269.1 || SS1G_08382.1 || SS1G_12988.1 || SS1G_03190.1
Alternative NADH	Acl	ACLA_094700 || ACLA_089240 || ACLA_023620
Alternative NADH	Pbl	Phybl172947 || Phybl180496 || Phybl174617 || Phybl123503 || Phybl129264
Alternative NADH	Pst	Picst366598
Alternative NADH	Cne	Q55P10 || Q55YT8
Alternative NADH	Sce	YDL085W || YMR145C || YML120C
Alternative NADH	Cgl	CAGL0I00748g || CAGL0B02431g
Alternative NADH	Lbi	Lacbi1311463 || Lacbi1306184 || Lacbi1192207
Alternative NADH	Bde	BDEG_02616
Alternative NADH	Clu	CLUG_04257.1
Alternative NADH	Cdu	protCdu2184 || protCdu4817 || protCdu4359
Alternative NADH	Cal	orf19.2175 || orf19.339 || orf19.5713
Alternative NADH	Sja	SJAG_01539 || SJAG_04152
Alternative NADH	Ncr	(NCU09447.2) || (NCU05225.2) || (NCU00153.2) || (NCU10873.2)
Alternative NADH	Smi	protSmi1408 || protSmi2332 || protSmi3659
Alternative NADH	Fox	FOXG_04003 || FOXG_08346 || FOXG_17236 || FOXG_16007 || FOXG_15549 || FOXG_02154 || FOXG_10437 || FOXG_11507
Alternative NADH	Cci	CC1G_06972.1 || CC1G_01142.1
Alternative NADH	Fve	FVEG_11034 || FVEG_12740 || FVEG_06327 || FVEG_05317 || FVEG_09091 || FVEG_10316
Alternative NADH	Sno	SNU13823.1 || SNU13957.1 || SNU03105.1 || SNU10743.1
Alternative NADH	Pan	protPan6510 || protPan3593 || protPan5926
Alternative NADH	Dha	Q6BSN0
Alternative NADH	Skl	protSkl3758 || protSkl316 || protSkl3922 || protSkl1921
Alternative NADH	Ath	Q9C5A3 || Q8GXR9 || Q9LML0 || O80874 || Q9SKT7 || O65414 || Q9S9T5 || Q9M0I5 || Q8LDE7
Alternative NADH	Mgr	MGG_04999.5 || MGG_04140.5 || MGG_06276.5
Alternative NADH	Sba	Sbay_67.20 || Sbay_16.57 || Sbay_65.162
Alternative NADH	Cim	CIMG_03156 || CIMG_00748 || CIMG_09051
Alternative NADH	Pgr	PGTG_06612 || PGTG_08874
Alternative NADH	Cgu	PGUG_02433.1 || PGUG_04603.1
Alternative NADH	Kpo	Kpol_1058.54 || Kpol_541.17
Alternative NADH	Ani	XP_682584.1 || XP_662911.1 || XP_680769.1 || XP_658698.1
Alternative NADH	Spb	O14121 || O43090
Alternative NADH	Ate	ATEG_08966.1 || ATEG_00653.1 || ATEG_06734.1 || ATEG_00394.1
Alternative NADH	Sca	Scas_680.13 || Scas_623.4 || Scas_692.10
Alternative NADH	Sku	protSku5010 || protSku2675 || protSku1973
Alternative NADH	Kla	XP_451367.1 || XP_452480.1 || XP_454942.1
Alternative NADH	Nha	Necha251665 || Necha2100191 || Necha2100859 || Necha2100932
Alternative NADH	Ang	A2QAR7 || A2QXL0 || A2QQZ5
Alternative NADH	Ppl	Pospl1104195 || Pospl120586 || Pospl1106667 || Pospl158339 || Pospl1104801 || Pospl162125
Alternative NADH	Yli	Q6C6X0 || O74931
Alternative NADH	Mfi	Mycfi183691 || Mycfi129791 || Mycfi187800 || Mycfi131160 || Mycfi188293
Alternative oxidase	Uma	UM02774.1
Alternative oxidase	Acl	ACLA_061560 || ACLA_089590
Alternative oxidase	Dha	Q6BVC1
Alternative oxidase	Pch	Phchr140093
Alternative oxidase	Fve	FVEG_01477
Alternative oxidase	Ncr	(NCU04874.2) || (NCU07953.2)
Alternative oxidase	Ani	XP_659703.1
Alternative oxidase	Afu	Q4WHK6
Alternative oxidase	Cne	Q560U5
Alternative oxidase	Cdu	protCdu6208 || protCdu6207
Alternative oxidase	Sro	Sporo112387
Alternative oxidase	Ppl	Pospl1108799 || Pospl1127449
Alternative oxidase	Ure	UREG_00996.1
Alternative oxidase	Ror	RO3G_15542.1
Alternative oxidase	Lbi	Lacbi1309224
Alternative oxidase	Aor	Q2U1I0 || Q2ULQ6

Alternative oxidase	Pbl	Phybl117724
Alternative oxidase	Ctr	CTRG_03270.3 || CTRG_03271.3
Alternative oxidase	Ang	A2QXC9 || A2QWD9
Alternative oxidase	Afl	AFL2G_04829 || AFL2G_02670
Alternative oxidase	Mgr	MGG_12936.5
Alternative oxidase	Hca	HCAG_03721.1
Alternative oxidase	Ssc	SS1G_02882.1
Alternative oxidase	Cgo	CHG05093.1 || CHG05032.1
Alternative oxidase	Ate	ATEG_07440.1 || ATEG_05999.1
Alternative oxidase	Pan	protPan9712
Alternative oxidase	Clu	CLUG_02042.1
Alternative oxidase	Sno	SNU03031.1
Alternative oxidase	Tre	Trire257940
Alternative oxidase	Fox	FOXG_00039
Alternative oxidase	Nfi	NFIA_081770
Alternative oxidase	Cal	orf19.4774 || orf19.4773
Alternative oxidase	Nha	Necha298640
Alternative oxidase	Cci	CC1G_03463.1 || CC1G_10695.1
Alternative oxidase	Lel	LELG_03594
Alternative oxidase	Ath	Q9C612 || Q9LQM4 || O22049 || O23913 || O22048 || Q9ZRT8 || Q39219
Alternative oxidase	Cim	CIMG_01051
Alternative oxidase	Mfi	Mycfi188001
Alternative oxidase	Gze	Q4IN66
Alternative oxidase	Yli	Q6C9M5 || Q8J0I8
Alternative oxidase	Bde	BDEG_05772
Alternative oxidase	Bci	BC1G_05703.1
Alternative oxidase	Cgu	PGUG_05793.1
Alternative oxidase	Pst	Picst367332
Complex I, 1.6.5.3	Pbl	Phybl19694 || Phybl115275
Complex I, 1.6.5.3	Cgo	CHG00836.1
Complex I, 1.6.5.3	Pgr	PGTG_06530
Complex I, 1.6.5.3	Tre	Trire22676
Complex I, 1.6.5.3	Aor	Q2UCY0
Complex I, 1.6.5.3	Ath	Q94C12 || Q9LS49 || Q9M9B4
Complex I, 1.6.5.3	Ure	UREG_01361.1
Complex I, 1.6.5.3	Nfi	NFIA_090140
Complex I, 1.6.5.3	Mgr	MGG_01267.5
Complex I, 1.6.5.3	Bci	BC1G_12783.1
Complex I, 1.6.5.3	Hca	HCAG_08298.1
Complex I, 1.6.5.3	Ssc	SS1G_13925.1
Complex I, 1.6.5.3	Cim	CIMG_01447
Complex I, 1.6.5.3	Cne	Q55SS5
Complex I, 1.6.5.3	Gze	Q4IP60
Complex I, 1.6.5.3	Afu	Q4X030
Complex I, 1.6.5.3	Ang	A2R5B4
Complex I, 1.6.5.3	Afl	AFL2G_03308
Complex I, 1.6.5.3	Sno	SNU04381.1
Complex I, 1.6.5.3	Ate	ATEG_07830.1
Complex I, 1.6.5.3	Mfi	Mycfi155284
Complex I, 1.6.5.3	Sro	Sporo17733
Complex I, 1.6.5.3	Ani	XP_680830.1
Complex I, 1.6.5.3	Ppl	Pospl138581
Complex I, 1.6.5.3	Ror	RO3G_14168.1 || RO3G_02491.1
Complex I, 1.6.5.3	Lbi	Lacbi1249657
Complex I, 1.6.5.3	Acl	ACLA_072580
Complex I, 1.6.5.3	Nha	Necha273373
Complex I, 1.6.5.3	Fox	FOXG_00397
Complex I, 1.6.5.3	Pan	protPan6143
Complex I, 1.6.5.3	Cci	CC1G_06429.1
Complex I, 1.6.5.3	Ncr	(NCU03093.2)
Complex I, 1.6.5.3	Uma	UM04766.1
Complex I, 1.6.5.3	Fve	FVEG_01120
Complex I, 1.6.5.3	Cal	orf19.3223.1
Complex I, NAD1	Pan	protPan12926
Complex I, NAD1	Cne	None
Complex I, NAD1	Cal	CaalfMp03
Complex I, NAD1	Pca	protPca3295
Complex I, NAD1	Ncr	Neur.crassa.mt15
Complex I, NAD1	Uma	UM_mit19
Complex I, NAD1	Yli	Q9B6E8
Complex I, NAD1	Ath	P92558 || Q37165
Complex I, NAD1	Hsa	ENSP00000354687
Complex I, NAD2	Pan	protPan12882
Complex I, NAD2	Cne	None
Complex I, NAD2	Hsa	ENSP00000355046
Complex I, NAD2	Ath	O05000 || Q3EC49 || Q9T3G4 || P93313
Complex I, NAD2	Yli	Q9B6C8
Complex I, NAD2	Cal	CaalfMp09
Complex I, NAD2	Ncr	Neur.crassa.mt16

Complex I, NAD2	Uma	UM_mit5
Complex I, NAD3	Cne	None
Complex I, NAD3	Uma	UM_mit6
Complex I, NAD3	Hsa	ENSP00000355206
Complex I, NAD3	Ath	P92533
Complex I, NAD3	Cal	CaalfMp10
Complex I, NAD3	Yli	Q9B6C7
Complex I, NAD4	Ath	P93313 || P26288
Complex I, NAD4	Sno	SNU16564.1
Complex I, NAD4	Yli	Q9B6D6
Complex I, NAD4	Cne	NADH
Complex I, NAD4	Pan	protPan12923
Complex I, NAD4	Cal	CaalfMp14
Complex I, NAD4	Hsa	ENSP00000354961
Complex I, NAD4	Cdu	protCdu3762
Complex I, NAD4	Uma	UM_mit18
Complex I, NAD4L	Yli	Q9B6D4
Complex I, NAD4L	Cdu	protCdu3757
Complex I, NAD4L	Cal	CaalfMp12
Complex I, NAD5	Yli	Q9B6D3 || Q9B6D2 || Q9B6D1
Complex I, NAD5	Cgu	PGUG_05917.1
Complex I, NAD5	Cal	CaalfMp13
Complex I, NAD5	Ncr	Neur.crassa.mt21
Complex I, NAD5	Uma	UM_mit24
Complex I, NAD5	Pan	protPan12913
Complex I, NAD5	Hsa	ENSP00000354813
Complex I, NAD5	Ath	P93313 || P26288 || P56752 || P29388
Complex I, NAD5	Cdu	protCdu3760
Complex I, NAD5	Cne	NADH || NADH
Complex I, NAD6	Pan	protPan12886
Complex I, NAD6	Cne	None
Complex I, NAD6	Ath	P60497
Complex I, NAD6	Uma	UM_mit26
Complex I, NAD6	Sro	Sporo116111
Complex I, Ndufa1	Ang	A2QJ43
Complex I, Ndufa1	Ncr	(NCU04781.2)
Complex I, Ndufa1	Nfi	NFIA_104880
Complex I, Ndufa1	Cci	CC1G_08864.1
Complex I, Ndufa1	Afl	AFL2G_01914
Complex I, Ndufa1	Afu	Q4WPY6
Complex I, Ndufa1	Sno	SNU06351.1
Complex I, Ndufa1	Dha	Q6BTU2
Complex I, Ndufa1	Fve	FVEG_02151
Complex I, Ndufa1	Gze	Q4I2S8
Complex I, Ndufa1	Pbl	Phybl135379
Complex I, Ndufa1	Mgr	MGG_04614.5
Complex I, Ndufa1	Acl	ACLA_050050
Complex I, Ndufa1	Hca	HCAG_01593.1
Complex I, Ndufa1	Lbi	Lacbi1290877
Complex I, Ndufa1	Uma	UM03043.1
Complex I, Ndufa1	Pgr	PGTG_04510
Complex I, Ndufa1	Cdu	protCdu1531
Complex I, Ndufa1	Tre	Trire282286
Complex I, Ndufa1	Ppl	Pospl148773
Complex I, Ndufa1	Sro	Sporo115905
Complex I, Ndufa1	Ssc	SS1G_00313.1
Complex I, Ndufa1	Cim	CIMG_01523
Complex I, Ndufa1	Pst	Picst377954
Complex I, Ndufa1	Bci	BC1G_00827.1
Complex I, Ndufa1	Fox	FOXG_03279
Complex I, Ndufa1	Ure	UREG_05259.1
Complex I, Ndufa1	Cne	Q55ZI9
Complex I, Ndufa1	Ror	RO3G_03061.1
Complex I, Ndufa11	Cim	CIMG_01782
Complex I, Ndufa11	Uma	UM02893.1
Complex I, Ndufa11	Gze	Q4I8X1
Complex I, Ndufa11	Cne	Q55KN3
Complex I, Ndufa11	Bci	BC1G_11395.1
Complex I, Ndufa11	Ure	UREG_05482.1
Complex I, Ndufa11	Hca	HCAG_00357.1 || HCAG_05309.1
Complex I, Ndufa11	Pgr	PGTG_07666
Complex I, Ndufa11	Sro	Sporo119020
Complex I, Ndufa11	Lbi	Lacbi1186871
Complex I, Ndufa11	Pca	protPca3845
Complex I, Ndufa11	Fve	FVEG_05012
Complex I, Ndufa11	Ang	A2QLI8
Complex I, Ndufa11	Afl	AFL2G_10464
Complex I, Ndufa11	Ani	XP_663274.1
Complex I, Ndufa11	Sno	SNU12121.1

Complex I, Ndufa11	Pch	Phchr16644
Complex I, Ndufa11	Cci	CC1G_10542.1
Complex I, Ndufa11	Mfi	Mycfi133688
Complex I, Ndufa11	Ppl	Pospl144560 || Pospl144904
Complex I, Ndufa11	Yli	Q6C674
Complex I, Ndufa11	Acl	ACLA_085520
Complex I, Ndufa11	Ath	O48528
Complex I, Ndufa11	Afu	Q4WLU6
Complex I, Ndufa11	Ssc	SS1G_11018.1
Complex I, Ndufa11	Mgr	MGG_00878.5
Complex I, Ndufa11	Nha	Necha259807
Complex I, Ndufa11	Ror	RO3G_07616.1 || RO3G_06554.1
Complex I, Ndufa11	Fox	FOXG_08089
Complex I, Ndufa11	Ate	ATEG_03294.1
Complex I, Ndufa11	Ncr	(NCU02280.2)
Complex I, Ndufa11	Tre	Trire2106834
Complex I, Ndufa11	Nfi	NFIA_058240
Complex I, Ndufa11	Cgo	CHG09612.1
Complex I, Ndufa11	Pbl	Phybl134597
Complex I, Ndufa12	Pch	Phchr11105
Complex I, Ndufa12	Ctr	CTRG_02400.3
Complex I, Ndufa12	Afu	Q4WTY5
Complex I, Ndufa12	Dha	Q6BJH4
Complex I, Ndufa12	Sro	Sporo17511
Complex I, Ndufa12	Mgr	MGG_07542.5
Complex I, Ndufa12	Pst	Picst344890
Complex I, Ndufa12	Ncr	(NCU01142.2)
Complex I, Ndufa12	Ath	Q9M9M9
Complex I, Ndufa12	Fve	FVEG_06660
Complex I, Ndufa12	Cgu	PGUG_00665.1
Complex I, Ndufa12	Ror	RO3G_04950.1 || RO3G_11038.1
Complex I, Ndufa12	Ani	XP_659864.1
Complex I, Ndufa12	Nfi	NFIA_036580
Complex I, Ndufa12	Fox	FOXG_09059
Complex I, Ndufa12	Lbi	Lacbi1149273
Complex I, Ndufa12	Cne	Q55RY7
Complex I, Ndufa12	Ate	ATEG_09372.1
Complex I, Ndufa12	Sno	SNU09335.1
Complex I, Ndufa12	Lel	LELG_02747
Complex I, Ndufa12	Cci	CC1G_01296.1
Complex I, Ndufa12	Clu	CLUG_02669.1
Complex I, Ndufa12	Hca	HCAG_09039.1
Complex I, Ndufa12	Afl	AFL2G_05834
Complex I, Ndufa12	Bci	BC1G_06544.1
Complex I, Ndufa12	Nha	Necha295277
Complex I, Ndufa12	Tre	Trire2106617
Complex I, Ndufa12	Hsa	ENSP00000330737
Complex I, Ndufa12	Acl	ACLA_009870
Complex I, Ndufa12	Cal	orf19.1625
Complex I, Ndufa12	Yli	Q6CG53
Complex I, Ndufa12	Ang	A2R9E8
Complex I, Ndufa12	Uma	UM04337.1
Complex I, Ndufa12	Mfi	Mycfi132657
Complex I, Ndufa12	Bde	BDEG_02122
Complex I, Ndufa12	Pbl	Phybl127870
Complex I, Ndufa12	Ppl	Pospl1115860
Complex I, Ndufa12	Cdu	protCdu4878
Complex I, Ndufa12	Pan	protPan5386
Complex I, Ndufa12	Pgr	PGTG_07636 || PGTG_02672
Complex I, Ndufa12	Gze	Q4I1A3
Complex I, Ndufa13	Sro	Sporo123600
Complex I, Ndufa13	Fox	FOXG_04069
Complex I, Ndufa13	Ure	UREG_07597.1
Complex I, Ndufa13	Nfi	NFIA_068350
Complex I, Ndufa13	Afu	Q4WXA2
Complex I, Ndufa13	Uma	UM01311.1
Complex I, Ndufa13	Pch	Phchr1139852
Complex I, Ndufa13	Ate	ATEG_01594.1
Complex I, Ndufa13	Hsa	ENSP00000252576
Complex I, Ndufa13	Fve	FVEG_07184
Complex I, Ndufa13	Ctr	CTRG_04003.3
Complex I, Ndufa13	Ror	RO3G_00378.1 || RO3G_08458.1
Complex I, Ndufa13	Nha	Necha2102228
Complex I, Ndufa13	Tre	Trire262389
Complex I, Ndufa13	Mgr	MGG_03486.5
Complex I, Ndufa13	Ncr	(NCU09299.2)
Complex I, Ndufa13	Acl	ACLA_036750
Complex I, Ndufa13	Aor	Q2UQ49
Complex I, Ndufa13	Sno	SNU15741.1

Complex I, Ndufa13	Cim	CIMG_07692
Complex I, Ndufa13	Pst	Picst376226
Complex I, Ndufa13	Cne	Q55NC6
Complex I, Ndufa13	Cci	CC1G_01335.1
Complex I, Ndufa13	Dha	Q6BUG1
Complex I, Ndufa13	Cdu	protCdu5792
Complex I, Ndufa13	Ani	XP_660615.1
Complex I, Ndufa13	Afl	AFL2G_01300
Complex I, Ndufa13	Bde	BDEG_02630
Complex I, Ndufa13	Ang	A2R734
Complex I, Ndufa13	Ssc	SS1G_07981.1
Complex I, Ndufa13	Gze	Q4I4K7
Complex I, Ndufa13	Ath	O49313 || Q8RWA7 || O23022
Complex I, Ndufa13	Mfi	Mycfi150654
Complex I, Ndufa13	Lbi	Lacbi1188614
Complex I, Ndufa13	Pgr	PGTG_02318
Complex I, Ndufa13	Hca	HCAG_04359.1
Complex I, Ndufa13	Ppl	Pospl1128470 || Pospl1115197
Complex I, Ndufa13	Pbl	Phybl137151
Complex I, Ndufa2	Cal	orf19.6898.1
Complex I, Ndufa2	Cci	CC1G_06520.1
Complex I, Ndufa2	Cdu	protCdu4000
Complex I, Ndufa2	Gze	Q4I2J2
Complex I, Ndufa2	Sro	Sporo111477
Complex I, Ndufa2	Nfi	NFIA_049290
Complex I, Ndufa2	Mfi	Mycfi129187
Complex I, Ndufa2	Bde	BDEG_04653
Complex I, Ndufa2	Ctr	CTRG_04848.3
Complex I, Ndufa2	Ppl	Pospl162187
Complex I, Ndufa2	Uma	UM01562.1
Complex I, Ndufa2	Cim	CIMG_10111
Complex I, Ndufa2	Hsa	ENSP00000252102
Complex I, Ndufa2	Lbi	Lacbi1189840
Complex I, Ndufa2	Pgr	PGTG_09726
Complex I, Ndufa2	Hca	HCAG_05081.1
Complex I, Ndufa2	Acl	ACLA_097460
Complex I, Ndufa2	Ncr	(NCU03156.2)
Complex I, Ndufa2	Ang	A2R062
Complex I, Ndufa2	Ani	XP_682032.1
Complex I, Ndufa2	Ath	Q9FIJ2
Complex I, Ndufa2	Pbl	Phybl124435
Complex I, Ndufa2	Ror	RO3G_06650.1
Complex I, Ndufa2	Cne	Q55J89
Complex I, Ndufa2	Sno	SNU07736.1
Complex I, Ndufa2	Tre	Trire265717
Complex I, Ndufa2	Bci	BC1G_09933.1
Complex I, Ndufa2	Afl	AFL2G_06531
Complex I, Ndufa2	Afu	Q4WCZ6
Complex I, Ndufa2	Lel	LELG_05455
Complex I, Ndufa2	Pst	Picst365836
Complex I, Ndufa2	Fox	FOXG_03380
Complex I, Ndufa2	Clu	CLUG_04278.1
Complex I, Ndufa2	Dha	Q6BKG9
Complex I, Ndufa2	Mgr	MGG_02669.5
Complex I, Ndufa2	Yli	Q6CD73
Complex I, Ndufa2	Ssc	SS1G_05418.1
Complex I, Ndufa2	Pch	Phchr1135812
Complex I, Ndufa2	Fve	FVEG_02252
Complex I, Ndufa2	Nha	Necha291987
Complex I, Ndufa2	Cgu	PGUG_00372.1
Complex I, Ndufa4	Cci	CC1G_00454.1
Complex I, Ndufa4	Uma	UM02800.1
Complex I, Ndufa4	Pgr	PGTG_12848
Complex I, Ndufa4	Ror	RO3G_11750.1 || RO3G_02769.1 || RO3G_11423.1
Complex I, Ndufa4	Pbl	Phybl115058 || Phybl177427
Complex I, Ndufa5	Gze	Q4ILG7
Complex I, Ndufa5	Ani	XP_663575.1
Complex I, Ndufa5	Acl	ACLA_069600
Complex I, Ndufa5	Ssc	SS1G_13352.1
Complex I, Ndufa5	Ctr	CTRG_00851.3
Complex I, Ndufa5	Bci	BC1G_14980.1
Complex I, Ndufa5	Ang	A2QD54
Complex I, Ndufa5	Pca	protPca1688
Complex I, Ndufa5	Ppl	Pospl1119864
Complex I, Ndufa5	Mfi	Mycfi164015
Complex I, Ndufa5	Uma	UM03931.1
Complex I, Ndufa5	Cal	orf19.2570
Complex I, Ndufa5	Nha	Necha272338
Complex I, Ndufa5	Ncr	(NCU05299.2)

Complex I, Ndufa5	Lel	LELG_00132
Complex I, Ndufa5	Sno	SNU12756.1
Complex I, Ndufa5	Yli	Q6C4W9
Complex I, Ndufa5	Pbl	Phybl135140
Complex I, Ndufa5	Aor	Q2U057
Complex I, Ndufa5	Cgu	PGUG_01435.1
Complex I, Ndufa5	Ror	RO3G_06118.1 || RO3G_15124.1
Complex I, Ndufa5	Pan	protPan10320
Complex I, Ndufa5	Hsa	ENSP00000257777
Complex I, Ndufa5	Bde	BDEG_08373
Complex I, Ndufa5	Ure	UREG_02221.1
Complex I, Ndufa5	Cci	CC1G_06737.1
Complex I, Ndufa5	Fox	FOXG_04188
Complex I, Ndufa5	Sro	Sporo120886
Complex I, Ndufa5	Afu	Q4X1B0
Complex I, Ndufa5	Tre	Trire252847
Complex I, Ndufa5	Lbi	Lacbi1186840
Complex I, Ndufa5	Hca	HCAG_08725.1
Complex I, Ndufa5	Cgo	CHG04840.1
Complex I, Ndufa5	Fve	FVEG_07304
Complex I, Ndufa5	Pch	Phchr16740
Complex I, Ndufa5	Ate	ATEG_01513.1
Complex I, Ndufa5	Cim	CIMG_07919
Complex I, Ndufa5	Pgr	PGTG_12946
Complex I, Ndufa5	Pst	Picst346630
Complex I, Ndufa5	Ath	Q9FLX7
Complex I, Ndufa5	Nfi	NFIA_085960
Complex I, Ndufa5	Cne	Q55I06
Complex I, Ndufa5	Mgr	MGG_06030.5
Complex I, Ndufa5	Afl	AFL2G_05329
Complex I, Ndufa5	Cdu	protCdu2391
Complex I, Ndufa5	Clu	CLUG_04573.1
Complex I, Ndufa5	Dha	Q6BM85
Complex I, Ndufa6	Pan	protPan12136
Complex I, Ndufa6	Sno	SNU09371.1
Complex I, Ndufa6	Cgo	CHG03294.1
Complex I, Ndufa6	Pst	Picst384570
Complex I, Ndufa6	Lbi	Lacbi1326056
Complex I, Ndufa6	Ror	RO3G_02063.1 || RO3G_04569.1
Complex I, Ndufa6	Ang	A2R4K0
Complex I, Ndufa6	Ppl	Pospl187762 || Pospl190422
Complex I, Ndufa6	Afl	AFL2G_05733
Complex I, Ndufa6	Ure	UREG_03059.1
Complex I, Ndufa6	Bde	BDEG_04382
Complex I, Ndufa6	Mgr	MGG_06310.5
Complex I, Ndufa6	Pbl	Phybl116029
Complex I, Ndufa6	Ctr	CTRG_04374.3
Complex I, Ndufa6	Cgu	PGUG_04737.1
Complex I, Ndufa6	Fve	FVEG_08261
Complex I, Ndufa6	Sro	Sporo114733
Complex I, Ndufa6	Ani	XP_664161.1
Complex I, Ndufa6	Pgr	PGTG_04698 || PGTG_20274
Complex I, Ndufa6	Lel	LELG_01485
Complex I, Ndufa6	Uma	UM02437.1
Complex I, Ndufa6	Clu	CLUG_04466.1
Complex I, Ndufa6	Nfi	NFIA_051150
Complex I, Ndufa6	Hsa	ENSP00000330937
Complex I, Ndufa6	Cdu	protCdu246
Complex I, Ndufa6	Cal	orf19.6035
Complex I, Ndufa6	Hca	HCAG_08225.1
Complex I, Ndufa6	Acl	ACLA_095940
Complex I, Ndufa6	Ncr	(NCU00418.2)
Complex I, Ndufa6	Nha	Necha2102572
Complex I, Ndufa6	Cne	Q55ZF0
Complex I, Ndufa6	Pch	Phchr15561
Complex I, Ndufa6	Mfi	Mycfi163363
Complex I, Ndufa6	Bci	BC1G_06579.1
Complex I, Ndufa6	Dha	Q6BSH5
Complex I, Ndufa6	Cim	CIMG_02709
Complex I, Ndufa6	Yli	Q6CI60
Complex I, Ndufa6	Ate	ATEG_06987.1
Complex I, Ndufa6	Gze	Q4I772
Complex I, Ndufa6	Ath	Q9LHI0
Complex I, Ndufa6	Afu	Q4WDG0
Complex I, Ndufa6	Ssc	SS1G_00589.1
Complex I, Ndufa6	Tre	Trire273846
Complex I, Ndufa6	Cci	CC1G_09433.1
Complex I, Ndufa6	Fox	FOXG_01884
Complex I, Ndufa8	Lbi	Lacbi1186286

Complex I, Ndufa8	Ani	XP_658667.1
Complex I, Ndufa8	Ath	Q9SQT4 || Q8LGE7
Complex I, Ndufa8	Pan	protPan1464
Complex I, Ndufa8	Cgu	PGUG_05443.1
Complex I, Ndufa8	Ate	ATEG_00428.1
Complex I, Ndufa8	Cim	CIMG_06906
Complex I, Ndufa8	Pst	Picst382538
Complex I, Ndufa8	Dha	Q6BQ05
Complex I, Ndufa8	Cdu	protCdu2300
Complex I, Ndufa8	Ang	A2QR63
Complex I, Ndufa8	Yli	Q6CGB4
Complex I, Ndufa8	Cal	orf19.2821
Complex I, Ndufa8	Cci	CC1G_08867.1
Complex I, Ndufa8	Afl	AFL2G_07525
Complex I, Ndufa8	Cgo	CHG06735.1
Complex I, Ndufa8	Ncr	(NCU02472.2)
Complex I, Ndufa8	Fve	FVEG_04096
Complex I, Ndufa8	Ror	RO3G_05071.1 || RO3G_06908.1
Complex I, Ndufa8	Ctr	CTRG_00836.3
Complex I, Ndufa8	Bci	BC1G_07911.1
Complex I, Ndufa8	Nfi	NFIA_013230
Complex I, Ndufa8	Acl	ACLA_023170
Complex I, Ndufa8	Nha	Necha266641
Complex I, Ndufa8	Aor	Q2UNM8
Complex I, Ndufa8	Clu	CLUG_05194.1
Complex I, Ndufa8	Mgr	MGG_03073.5
Complex I, Ndufa8	Bde	BDEG_06189
Complex I, Ndufa8	Gze	Q4HZR1
Complex I, Ndufa8	Tre	Trire2111033
Complex I, Ndufa8	Sno	SNU01289.1
Complex I, Ndufa8	Mfi	Mycfi142743
Complex I, Ndufa8	Pbl	Phybl132562
Complex I, Ndufa8	Ssc	SS1G_03473.1
Complex I, Ndufa8	Cne	Q55LV7
Complex I, Ndufa8	Ppl	Pospl1119560 || Pospl1110271
Complex I, Ndufa8	Pgr	PGTG_04591
Complex I, Ndufa8	Hsa	ENSP00000238369
Complex I, Ndufa8	Uma	UM05598.1
Complex I, Ndufa8	Afu	Q4WSM3
Complex I, Ndufa8	Sro	Sporo134021
Complex I, Ndufa8	Hca	HCAG_01733.1
Complex I, Ndufa8	Ure	UREG_05940.1
Complex I, Ndufa8	Fox	FOXG_06244
Complex I, Ndufa8	Lel	LELG_00111
Complex I, Ndufa8	Pch	Phchr1129717
Complex I, Ndufa9	Pan	protPan10709
Complex I, Ndufa9	Uma	UM00381.1
Complex I, Ndufa9	Hca	HCAG_05248.1
Complex I, Ndufa9	Ror	RO3G_11657.1
Complex I, Ndufa9	Afl	AFL2G_02591
Complex I, Ndufa9	Cim	CIMG_01717
Complex I, Ndufa9	Sno	SNU12004.1
Complex I, Ndufa9	Ang	A2QSH0
Complex I, Ndufa9	Pch	Phchr1128800
Complex I, Ndufa9	Hsa	ENSP00000266544
Complex I, Ndufa9	Cgo	CHG00409.1
Complex I, Ndufa9	Mfi	Mycfi160941
Complex I, Ndufa9	Mgr	MGG_03525.5
Complex I, Ndufa9	Cgu	PGUG_00563.1
Complex I, Ndufa9	Ctr	CTRG_02150.3
Complex I, Ndufa9	Yli	Q6C7X4
Complex I, Ndufa9	Dha	Q6BRM2
Complex I, Ndufa9	Afu	Q4WLP5
Complex I, Ndufa9	Gze	Q4IEI7 || Q4I4A1
Complex I, Ndufa9	Ani	XP_661322.1
Complex I, Ndufa9	Lbi	Lacbi1243354 || Lacbi1230624
Complex I, Ndufa9	Fox	FOXG_13860 || FOXG_05080 || FOXG_03709
Complex I, Ndufa9	Pgr	PGTG_12713
Complex I, Ndufa9	Bci	BC1G_16156.1
Complex I, Ndufa9	Ppl	Pospl1111199
Complex I, Ndufa9	Pst	Picst376018
Complex I, Ndufa9	Tre	Trire277353
Complex I, Ndufa9	Acl	ACLA_086040
Complex I, Ndufa9	Aor	Q2ULI3
Complex I, Ndufa9	Cne	Q560L2 || Q55TD0
Complex I, Ndufa9	Cdu	protCdu5075
Complex I, Ndufa9	Sro	Sporo113603
Complex I, Ndufa9	Lel	LELG_02225
Complex I, Ndufa9	Pbl	Phybl176352

Complex I, Ndufa9	Nfi	NFIA_058750
Complex I, Ndufa9	Ncr	(NCU02373.2)
Complex I, Ndufa9	Cal	orf19.1682
Complex I, Ndufa9	Ssc	SS1G_11051.1
Complex I, Ndufa9	Cci	CC1G_01817.1 || CC1G_03467.1
Complex I, Ndufa9	Ath	O65502 || Q9SK66
Complex I, Ndufa9	Ate	ATEG_03315.1
Complex I, Ndufa9	Clu	CLUG_01993.1
Complex I, Ndufa9	Fve	FVEG_11290 || FVEG_12156
Complex I, Ndufa9	Bde	BDEG_06693
Complex I, Ndufa9	Nha	Necha267928
Complex I, Ndufab1	Cim	CIMG_03997
Complex I, Ndufab1	Cal	orf19.819 || orf19.2439
Complex I, Ndufab1	Pan	protPan12417
Complex I, Ndufab1	Cgu	PGUG_00711.1 || PGUG_04160.1
Complex I, Ndufab1	Dha	Q6BS82 || Q6BUS3
Complex I, Ndufab1	Ago	NP_984963.1
Complex I, Ndufab1	Acl	ACLA_027950
Complex I, Ndufab1	Ppl	Pospl1119973
Complex I, Ndufab1	Skl	protSkl3956
Complex I, Ndufab1	Tre	Trire278482
Complex I, Ndufab1	Ani	XP_663308.1
Complex I, Ndufab1	Spa	protSpa2832
Complex I, Ndufab1	Ssc	SS1G_11899.1
Complex I, Ndufab1	Hca	HCAG_05510.1
Complex I, Ndufab1	Smi	protSmi121
Complex I, Ndufab1	Pgr	PGTG_13047 || PGTG_18772
Complex I, Ndufab1	Fox	FOXG_04083
Complex I, Ndufab1	Clu	CLUG_00159.1 || CLUG_03732.1
Complex I, Ndufab1	Sca	Scas_719.45
Complex I, Ndufab1	Afl	AFL2G_09107
Complex I, Ndufab1	Cdu	protCdu1608 || protCdu5879
Complex I, Ndufab1	Yli	Q6C926 || Q6C7X2
Complex I, Ndufab1	Sno	SNU02719.1
Complex I, Ndufab1	Lel	LELG_00775 || LELG_01975
Complex I, Ndufab1	Sba	Sbay_53.27
Complex I, Ndufab1	Hsa	ENSP00000007516
Complex I, Ndufab1	Sce	YKL192C
Complex I, Ndufab1	Ctr	CTRG_01451.3 || CTRG_04269.3
Complex I, Ndufab1	Afu	Q4WJA9
Complex I, Ndufab1	Lbi	Lacbi1164630
Complex I, Ndufab1	Kpo	Kpol_1052.31
Complex I, Ndufab1	Ath	P25701 || P25702 || O04652 || P11829 || O80800 || P53665 || Q9FGJ4
Complex I, Ndufab1	Ncr	(NCU05008.2)
Complex I, Ndufab1	Uma	UM00778.1
Complex I, Ndufab1	Sja	SJAG_01238
Complex I, Ndufab1	Pbl	Phybl19165 || Phybl126643 || Phybl134052
Complex I, Ndufab1	Pch	Phchr16585
Complex I, Ndufab1	Cne	Q55U89 || Q55UB5
Complex I, Ndufab1	Kla	XP_453194.1
Complex I, Ndufab1	Spb	Q10217
Complex I, Ndufab1	Nfi	NFIA_018070
Complex I, Ndufab1	Pst	Picst380821 || Picst371972
Complex I, Ndufab1	Fve	FVEG_07197
Complex I, Ndufab1	Sro	Sporo119011
Complex I, Ndufab1	Nha	Necha2102223
Complex I, Ndufab1	Ang	A2RB66
Complex I, Ndufab1	Kwa	Kwal_47.17140
Complex I, Ndufab1	Cgl	CAGL0J04664g || CAGL0D03586g
Complex I, Ndufab1	Cgo	CHG00048.1
Complex I, Ndufab1	Sku	protSku2436
Complex I, Ndufab1	Mfi	Mycfi161135
Complex I, Ndufab1	Cci	CC1G_06777.1
Complex I, Ndufab1	Mgr	MGG_03484.5
Complex I, Ndufab1	Ror	RO3G_07961.1 || RO3G_16841.1 || RO3G_12225.1
Complex I, Ndufab1	Gze	Q4I4J3
Complex I, Ndufab1	Ate	ATEG_02960.1
Complex I, Ndufb3	Gze	Q4IB44
Complex I, Ndufb3	Sno	SNU09148.1
Complex I, Ndufb3	Afl	AFL2G_05941
Complex I, Ndufb3	Acl	ACLA_010650
Complex I, Ndufb3	Pbl	Phybl19775
Complex I, Ndufb3	Mgr	MGG_01667.5
Complex I, Ndufb3	Ppl	Pospl162325
Complex I, Ndufb3	Ani	XP_680898.1
Complex I, Ndufb3	Mfi	Mycfi160307
Complex I, Ndufb3	Ssc	SS1G_12460.1
Complex I, Ndufb3	Dha	Q6BUH9
Complex I, Ndufb3	Ncr	(NCU09002.2)

Complex I, Ndufb3	Cim	CIMG_06110
Complex I, Ndufb3	Fox	FOXG_09326
Complex I, Ndufb3	Uma	UM05102.1
Complex I, Ndufb3	Ate	ATEG_08104.1
Complex I, Ndufb3	Hca	HCAG_02881.1
Complex I, Ndufb3	Pgr	PGTG_17172
Complex I, Ndufb3	Cdu	protCdu5747
Complex I, Ndufb3	Pst	Picst363166
Complex I, Ndufb3	Ror	RO3G_14590.1 || RO3G_14943.1
Complex I, Ndufb3	Cne	Q55N39
Complex I, Ndufb3	Fve	FVEG_06930
Complex I, Ndufb3	Nfi	NFIA_079880
Complex I, Ndufb3	Afu	Q4WU68
Complex I, Ndufb3	Lbi	Lacbi1313705
Complex I, Ndufb3	Bci	BC1G_03652.1
Complex I, Ndufb3	Tre	Trire234327
Complex I, Ndufb4	Pst	Picst337347 || Picst330771 || Picst332634 || Picst365867
Complex I, Ndufb4	Ani	XP_660841.1 || XP_680854.1 || XP_664716.1 || XP_681969.1 || XP_660868.1
Complex I, Ndufb4	Cim	CIMG_04893 || CIMG_05866 || CIMG_08770
Complex I, Ndufb4	Uma	UM05414.1
Complex I, Ndufb4	Sno	SNU03261.1 || SNU10149.1 || SNU06160.1 || SNU00428.1 || SNU00458.1 || SNU05545.1 || SNU08883.1 || SNU06625.1 || SNU13682.1 || SNU09619.1 || SNU15949.1 || SNU00314.1 || SNU10851.1
Complex I, Ndufb4	Ppl	Pospl139403
Complex I, Ndufb4	Cdu	protCdu6631 || protCdu5735
Complex I, Ndufb4	Mfi	Mycfi127220 || Mycfi138754 || Mycfi137273 || Mycfi180615 || Mycfi131103 || Mycfi129581 || Mycfi187113 || Mycfi154520 || Mycfi158758 || Mycfi126982 || Mycfi150120 || Mycfi181471
Complex I, Ndufb4	Hca	HCAG_04330.1
Complex I, Ndufb4	Ang	A2QTT1 || A2QG42 || A2QFT3 || A2QT27 || A2R949 || A2R0E7 || A2R2J6
Complex I, Ndufb4	Cal	orf19.7554 || orf19.7336 || orf19.4779
Complex I, Ndufb4	Gze	Q4IGW7 || Q4IHE8 || Q4IPZ7 || Q4IAL0
Complex I, Ndufb4	Ror	RO3G_16094.1 || RO3G_09431.1 || RO3G_11434.1 || RO3G_02798.1
Complex I, Ndufb4	Dha	Q6BQR3
Complex I, Ndufb4	Ncr	(NCU07736.2) || (NCU02023.2) || (NCU10910.2) || (NCU03789.2)
Complex I, Ndufb4	Mgr	MGG_10469.5
Complex I, Ndufb4	Lel	LELG_05322 || LELG_03789 || LELG_00428
Complex I, Ndufb4	Nfi	NFIA_062530 || NFIA_048660 || NFIA_049680 || NFIA_029480
Complex I, Ndufb4	Afl	AFL2G_04482 || AFL2G_08855 || AFL2G_08158 || AFL2G_05122
Complex I, Ndufb4	Ate	ATEG_07212.1 || ATEG_02223.1
Complex I, Ndufb4	Acl	ACLA_062390 || ACLA_048160 || ACLA_098070 || ACLA_054520
Complex I, Ndufb4	Cgu	PGUG_04556.1
Complex I, Ndufb4	Nha	Necha245051 || Necha214093 || Necha286247 || Necha256320 || Necha253897 || Necha245233 || Necha265858 || Necha296892 || Necha274179 || Necha236043 || Necha290686
Complex I, Ndufb4	Pan	protPan4164 || protPan6228 || protPan10050 || protPan7256 || protPan11070
Complex I, Ndufb4	Bci	BC1G_11205.1 || BC1G_07163.1 || BC1G_10168.1 || BC1G_11077.1 || BC1G_09467.1 || BC1G_00699.1 || BC1G_16101.1
Complex I, Ndufb4	Aor	Q2UGZ0 || Q2U3H2 || Q2U6R4 || Q2U851 || Q8J1K9 || Q2U0R4
Complex I, Ndufb4	Sro	Sporo112877 || Sporo11678
Complex I, Ndufb4	Fve	FVEG_03318 || FVEG_10806 || FVEG_13247 || FVEG_11059 || FVEG_05882
Complex I, Ndufb4	Pch	Phchr137620 || Phchr1136183 || Phchr1128220 || Phchr137613 || Phchr1122125 || Phchr1132234 || Phchr1132081
Complex I, Ndufb4	Cgo	CHG06798.1 || CHG06526.1 || CHG09967.1
Complex I, Ndufb4	Ure	UREG_03087.1 || UREG_02712.1
Complex I, Ndufb4	Fox	FOXG_04824 || FOXG_12319 || FOXG_13636 || FOXG_08788
Complex I, Ndufb4	Ssc	SS1G_05145.1 || SS1G_12128.1 || SS1G_05556.1
Complex I, Ndufb4	Afu	Q4WX79 || Q4WYY8 || Q4WCT9 || Q4WD32 || Q4W9S2
Complex I, Ndufb4	Ctr	CTRG_05923.3 || CTRG_03958.3 || CTRG_03960.3 || CTRG_04131.3
Complex I, Ndufb4	Pbl	Phybl111742 || Phybl160066
Complex I, Ndufb7	Cal	orf19.446.2
Complex I, Ndufb7	Dha	Q6BQ95
Complex I, Ndufb7	Pst	Picst369376
Complex I, Ndufb7	Cci	CC1G_07633.1
Complex I, Ndufb7	Lbi	Lacbi1174092
Complex I, Ndufb8	Mgr	MGG_02184.5
Complex I, Ndufb8	Cne	Q560D9
Complex I, Ndufb8	Sno	SNU11568.1
Complex I, Ndufb8	Afl	AFL2G_07938
Complex I, Ndufb8	Cgu	PGUG_00423.1
Complex I, Ndufb8	Aor	Q2U237
Complex I, Ndufb8	Lel	LELG_02479
Complex I, Ndufb8	Ssc	SS1G_14269.1
Complex I, Ndufb8	Gze	Q4IAK8
Complex I, Ndufb8	Ani	XP_682347.1
Complex I, Ndufb8	Fox	FOXG_08786
Complex I, Ndufb8	Pbl	Phybl136429
Complex I, Ndufb8	Ctr	CTRG_01700.3
Complex I, Ndufb8	Fve	FVEG_05884
Complex I, Ndufb8	Ang	A2QY72
Complex I, Ndufb8	Nha	Necha2102412
Complex I, Ndufb8	Pan	protPan3798
Complex I, Ndufb8	Acl	ACLA_065150

Complex I, Ndufb8	Nfi	NFIA_115110
Complex I, Ndufb8	Ure	UREG_02093.1
Complex I, Ndufb8	Afu	Q4WAS8
Complex I, Ndufb8	Cdu	protCdu5267
Complex I, Ndufb8	Mfi	Mycfi158202
Complex I, Ndufb8	Clu	CLUG_04613.1
Complex I, Ndufb8	Ncr	(NCU09460.2)
Complex I, Ndufb8	Bci	BC1G_12647.1
Complex I, Ndufb8	Cgo	CHG11060.1
Complex I, Ndufb8	Tre	Trire221606
Complex I, Ndufb8	Ror	RO3G_02334.1 || RO3G_14313.1
Complex I, Ndufb8	Ate	ATEG_01868.1
Complex I, Ndufb8	Cim	CIMG_07735
Complex I, Ndufb8	Pgr	PGTG_08821
Complex I, Ndufb8	Hca	HCAG_04394.1
Complex I, Ndufb9	Cim	CIMG_02131
Complex I, Ndufb9	Ath	O65692 || Q945M1
Complex I, Ndufb9	Afl	AFL2G_11261
Complex I, Ndufb9	Mgr	MGG_03576.5
Complex I, Ndufb9	Clu	CLUG_04957.1
Complex I, Ndufb9	Fve	FVEG_05101
Complex I, Ndufb9	Cdu	protCdu4361
Complex I, Ndufb9	Pst	Picst379236
Complex I, Ndufb9	Pch	Phchr11575
Complex I, Ndufb9	Lel	LELG_04969
Complex I, Ndufb9	Sro	Sporo111077
Complex I, Ndufb9	Cgu	PGUG_03432.1
Complex I, Ndufb9	Cgo	CHG00468.1
Complex I, Ndufb9	Uma	UM05625.1
Complex I, Ndufb9	Ror	RO3G_00955.1 || RO3G_07193.1
Complex I, Ndufb9	Nha	Necha29923
Complex I, Ndufb9	Bci	BC1G_11315.1
Complex I, Ndufb9	Ctr	CTRG_05748.3
Complex I, Ndufb9	Lbi	Lacbi1246507
Complex I, Ndufb9	Pan	protPan10796
Complex I, Ndufb9	Gze	Q4HTT0
Complex I, Ndufb9	Cal	orf19.5547
Complex I, Ndufb9	Ppl	Pospl142785
Complex I, Ndufb9	Mfi	Mycfi144927
Complex I, Ndufb9	Hca	HCAG_03523.1
Complex I, Ndufb9	Pbl	Phybl131750
Complex I, Ndufb9	Cci	CC1G_01387.1
Complex I, Ndufb9	Tre	Trire247510
Complex I, Ndufb9	Sno	SNU14610.1
Complex I, Ndufb9	Dha	Q6BNR1
Complex I, Ndufb9	Acl	ACLA_033380
Complex I, Ndufb9	Ate	ATEG_09001.1
Complex I, Ndufb9	Fox	FOXG_08174
Complex I, Ndufb9	Ssc	SS1G_10958.1
Complex I, Ndufb9	Cne	Q55SN6
Complex I, Ndufb9	Bde	BDEG_07764
Complex I, Ndufb9	Hsa	ENSP00000276689
Complex I, Ndufb9	Yli	Q6C9Z1
Complex I, Ndufb9	Ani	XP_661071.1
Complex I, Ndufb9	Ang	A2QY47
Complex I, Ndufs1	Tre	Trire244041
Complex I, Ndufs1	Bde	BDEG_06300
Complex I, Ndufs1	Afu	Q4WEV9
Complex I, Ndufs1	Ppl	Pospl1118959
Complex I, Ndufs1	Acl	ACLA_000760
Complex I, Ndufs1	Ath	Q9FGI6 || Q8L7R7
Complex I, Ndufs1	Aor	Q2U9W0
Complex I, Ndufs1	Ncr	(NCU01765.2)
Complex I, Ndufs1	Ssc	SS1G_01767.1
Complex I, Ndufs1	Cgu	PGUG_02889.1
Complex I, Ndufs1	Lel	LELG_05286
Complex I, Ndufs1	Fve	FVEG_04906
Complex I, Ndufs1	Pca	protPca3654
Complex I, Ndufs1	Sno	SNU01470.1
Complex I, Ndufs1	Pan	protPan2273
Complex I, Ndufs1	Pgr	PGTG_06894
Complex I, Ndufs1	Cgo	CHG10220.1
Complex I, Ndufs1	Ure	UREG_04070.1
Complex I, Ndufs1	Cdu	protCdu6647
Complex I, Ndufs1	Ctr	CTRG_05824.3
Complex I, Ndufs1	Gze	Q4IC60
Complex I, Ndufs1	Pbl	Phybl177432 || Phybl136090
Complex I, Ndufs1	Uma	UM04822.1
Complex I, Ndufs1	Hca	HCAG_06929.1

Complex I, Ndufs1	Fox	FOXG_07987
Complex I, Ndufs1	Dha	Q6BJ11
Complex I, Ndufs1	Ate	ATEG_07974.1
Complex I, Ndufs1	Lbi	Lacbi1192128
Complex I, Ndufs1	Ror	RO3G_13384.1 || RO3G_12339.1
Complex I, Ndufs1	Clu	CLUG_05783.1
Complex I, Ndufs1	Ang	A2QUU8
Complex I, Ndufs1	Nfi	NFIA_037700
Complex I, Ndufs1	Yli	Q9UUU3
Complex I, Ndufs1	Hsa	ENSP00000233190
Complex I, Ndufs1	Ani	XP_661892.1
Complex I, Ndufs1	Cne	Q55XC1
Complex I, Ndufs1	Mgr	MGG_03155.5
Complex I, Ndufs1	Pst	Picst385822
Complex I, Ndufs1	Afl	AFL2G_10016
Complex I, Ndufs1	Cci	CC1G_11265.1
Complex I, Ndufs1	Nha	Necha268136
Complex I, Ndufs1	Pch	Phchr1131257
Complex I, Ndufs1	Cim	CIMG_06326
Complex I, Ndufs1	Mfi	Mycfi149504
Complex I, Ndufs1	Sro	Sporo120832
Complex I, Ndufs1	Cal	orf19.7590
Complex I, Ndufs2	Ssc	SS1G_02958.1
Complex I, Ndufs2	Fve	FVEG_00831
Complex I, Ndufs2	Ror	RO3G_07618.1 || RO3G_06555.1
Complex I, Ndufs2	Ate	ATEG_01355.1
Complex I, Ndufs2	Afl	AFL2G_07022
Complex I, Ndufs2	Bde	BDEG_04061
Complex I, Ndufs2	Ang	A2QD14
Complex I, Ndufs2	Nfi	NFIA_088890
Complex I, Ndufs2	Pca	protPca1892
Complex I, Ndufs2	Gze	Q4IQY2
Complex I, Ndufs2	Cci	CC1G_00725.1
Complex I, Ndufs2	Cal	orf19.6531
Complex I, Ndufs2	Sno	SNU04659.1
Complex I, Ndufs2	Aor	Q2UFF4
Complex I, Ndufs2	Pgr	PGTG_03025
Complex I, Ndufs2	Ctr	CTRG_05173.3
Complex I, Ndufs2	Ure	UREG_01832.1
Complex I, Ndufs2	Dha	Q6BLW6
Complex I, Ndufs2	Cne	Q55IQ6
Complex I, Ndufs2	Tre	Trire276159
Complex I, Ndufs2	Cdu	protCdu3894
Complex I, Ndufs2	Pch	Phchr1126052
Complex I, Ndufs2	Sro	Sporo13376
Complex I, Ndufs2	Fox	FOXG_00680
Complex I, Ndufs2	Ncr	(NCU02534.2)
Complex I, Ndufs2	Afu	Q4X0E9
Complex I, Ndufs2	Mgr	MGG_09285.5
Complex I, Ndufs2	Nha	Necha290188
Complex I, Ndufs2	Ppl	Pospl1116553 || Pospl1117054
Complex I, Ndufs2	Mfi	Mycfi187355
Complex I, Ndufs2	Hca	HCAG_04623.1
Complex I, Ndufs2	Ani	XP_660018.1
Complex I, Ndufs2	Yli	Q9UUU1
Complex I, Ndufs2	Uma	UM04924.1
Complex I, Ndufs2	Clu	CLUG_01453.1
Complex I, Ndufs2	Hsa	ENSP00000289897
Complex I, Ndufs2	Lel	LELG_05138
Complex I, Ndufs2	Cgu	PGUG_04269.1
Complex I, Ndufs2	Pst	Picst368160
Complex I, Ndufs2	Cim	CIMG_04633
Complex I, Ndufs2	Acl	ACLA_073930
Complex I, Ndufs2	Cgo	CHG01092.1
Complex I, Ndufs2	Bci	BC1G_02234.1
Complex I, Ndufs2	Ath	P56753 || P93306
Complex I, Ndufs2	Pbl	Phybl157491
Complex I, Ndufs2	Lbi	Lacbi1186248 || Lacbi1303351 || Lacbi1144404 || Lacbi1144780
Complex I, Ndufs2	Pan	protPan12672
Complex I, Ndufs3	Ppl	Pospl1113660 || Pospl1111690
Complex I, Ndufs3	Ath	Q95748
Complex I, Ndufs3	Pgr	PGTG_06714
Complex I, Ndufs3	Yli	Q9UUU0
Complex I, Ndufs3	Bci	BC1G_04333.1
Complex I, Ndufs3	Gze	Q4I0M4
Complex I, Ndufs3	Lel	LELG_02077
Complex I, Ndufs3	Ncr	(NCU04074.2)
Complex I, Ndufs3	Nha	Necha266863
Complex I, Ndufs3	Afu	Q4WMT2

Complex I, Ndufs3	Cal	orf19.1710
Complex I, Ndufs3	Lbi	Lacbi1170574
Complex I, Ndufs3	Sro	Sporo117954
Complex I, Ndufs3	Pst	Picst374163
Complex I, Ndufs3	Acl	ACLA_083600
Complex I, Ndufs3	Cci	CC1G_04419.1
Complex I, Ndufs3	Sno	SNU06383.1
Complex I, Ndufs3	Ror	RO3G_06497.1
Complex I, Ndufs3	Mgr	MGG_01333.5
Complex I, Ndufs3	Pch	Phchr1124695
Complex I, Ndufs3	Pan	protPan1969
Complex I, Ndufs3	Ang	A2QWS1
Complex I, Ndufs3	Cim	CIMG_01359
Complex I, Ndufs3	Ssc	SS1G_02230.1
Complex I, Ndufs3	Mfi	Mycfi186282
Complex I, Ndufs3	Nfi	NFIA_054460
Complex I, Ndufs3	Ani	XP_659332.1
Complex I, Ndufs3	Cne	Q55R46
Complex I, Ndufs3	Ate	ATEG_06630.1
Complex I, Ndufs3	Ure	UREG_01282.1
Complex I, Ndufs3	Dha	Q6BY76
Complex I, Ndufs3	Clu	CLUG_04416.1
Complex I, Ndufs3	Uma	UM01681.1
Complex I, Ndufs3	Tre	Trire222816
Complex I, Ndufs3	Pbl	Phybl155767
Complex I, Ndufs3	Afl	AFL2G_09198
Complex I, Ndufs3	Cdu	protCdu4931
Complex I, Ndufs3	Ctr	CTRG_02172.3
Complex I, Ndufs3	Bde	BDEG_00201 || BDEG_00201
Complex I, Ndufs3	Fve	FVEG_03873
Complex I, Ndufs3	Aor	Q2UN03
Complex I, Ndufs3	Cgu	PGUG_01928.1
Complex I, Ndufs3	Hsa	ENSP00000263774
Complex I, Ndufs3	Cgo	CHG09244.1
Complex I, Ndufs3	Hca	HCAG_05764.1
Complex I, Ndufs3	Fox	FOXG_06006
Complex I, Ndufs4	Hca	HCAG_00972.1
Complex I, Ndufs4	Ncr	(NCU05221.2)
Complex I, Ndufs4	Cgu	PGUG_00320.1
Complex I, Ndufs4	Ssc	SS1G_13868.1
Complex I, Ndufs4	Yli	Q6CEK9
Complex I, Ndufs4	Lel	LELG_01368
Complex I, Ndufs4	Cal	orf19.3290
Complex I, Ndufs4	Dha	Q6BKL8
Complex I, Ndufs4	Ang	A2QXL4
Complex I, Ndufs4	Pbl	Phybl121604
Complex I, Ndufs4	Tre	Trire255088
Complex I, Ndufs4	Acl	ACLA_089200
Complex I, Ndufs4	Gze	Q4IJY4
Complex I, Ndufs4	Ror	RO3G_13870.1 || RO3G_13157.1
Complex I, Ndufs4	Bci	BC1G_13852.1
Complex I, Ndufs4	Ctr	CTRG_04402.3
Complex I, Ndufs4	Ate	ATEG_06737.1
Complex I, Ndufs4	Nfi	NFIA_082220
Complex I, Ndufs4	Bde	BDEG_02771
Complex I, Ndufs4	Cgo	CHG03791.1
Complex I, Ndufs4	Afu	Q4WHG4
Complex I, Ndufs4	Cne	Q55I49
Complex I, Ndufs4	Pch	Phchr14961
Complex I, Ndufs4	Afl	AFL2G_09298
Complex I, Ndufs4	Cci	CC1G_07164.1
Complex I, Ndufs4	Pan	protPan6477
Complex I, Ndufs4	Pgr	PGTG_10558
Complex I, Ndufs4	Fox	FOXG_10434
Complex I, Ndufs4	Ppl	Pospl1117483
Complex I, Ndufs4	Sro	Sporo16640
Complex I, Ndufs4	Sno	SNU08966.1
Complex I, Ndufs4	Cdu	protCdu262
Complex I, Ndufs4	Mfi	Mycfi156926
Complex I, Ndufs4	Nha	Necha270137
Complex I, Ndufs4	Ani	XP_680766.1
Complex I, Ndufs4	Hsa	ENSP00000296684
Complex I, Ndufs4	Fve	FVEG_09088
Complex I, Ndufs4	Mgr	MGG_04136.5
Complex I, Ndufs4	Pst	Picst362206
Complex I, Ndufs4	Cim	CIMG_00752
Complex I, Ndufs4	Aor	Q2UMQ7
Complex I, Ndufs4	Ath	Q9FJW4
Complex I, Ndufs4	Clu	CLUG_03938.1

Complex I, Ndufs4	Lbi	Lacbi1148506
Complex I, Ndufs4	Uma	UM00512.1
Complex I, Ndufs6	Nfi	NFIA_040040
Complex I, Ndufs6	Dha	Q6BWG5
Complex I, Ndufs6	Afu	Q4WE81
Complex I, Ndufs6	Cdu	protCdu2211
Complex I, Ndufs6	Hca	HCAG_00338.1
Complex I, Ndufs6	Sno	SNU11010.1
Complex I, Ndufs6	Fox	FOXG_01910
Complex I, Ndufs6	Pgr	PGTG_05065
Complex I, Ndufs6	Lbi	Lacbi1300649
Complex I, Ndufs6	Cal	orf19.3611
Complex I, Ndufs6	Bci	BC1G_10902.1
Complex I, Ndufs6	Mfi	Mycfi156511
Complex I, Ndufs6	Cgu	PGUG_01736.1
Complex I, Ndufs6	Aor	Q2UJ91
Complex I, Ndufs6	Yli	Q6C8J9
Complex I, Ndufs6	Ssc	SS1G_08945.1
Complex I, Ndufs6	Fve	FVEG_14032
Complex I, Ndufs6	Nha	Necha279136
Complex I, Ndufs6	Pst	Picst358506
Complex I, Ndufs6	Ath	Q9M9M6
Complex I, Ndufs6	Tre	Trire243884
Complex I, Ndufs6	Cci	CC1G_11299.1
Complex I, Ndufs6	Bde	BDEG_07619
Complex I, Ndufs6	Sro	Sporo110162
Complex I, Ndufs6	Cim	CIMG_06415
Complex I, Ndufs6	Pbl	Phybl135566
Complex I, Ndufs6	Ctr	CTRG_01863.3
Complex I, Ndufs6	Ure	UREG_03992.1
Complex I, Ndufs6	Lel	LELG_02763
Complex I, Ndufs6	Hsa	ENSP00000274137
Complex I, Ndufs6	Mgr	MGG_05668.5
Complex I, Ndufs6	Cne	Q55I93
Complex I, Ndufs6	Gze	Q4I799
Complex I, Ndufs6	Acl	ACLA_003190
Complex I, Ndufs6	Afl	AFL2G_01755
Complex I, Ndufs6	Pan	protPan11136
Complex I, Ndufs6	Ani	XP_681318.1
Complex I, Ndufs6	Cgo	CHG03143.1
Complex I, Ndufs6	Ate	ATEG_09826.1
Complex I, Ndufs6	Ror	RO3G_11801.1 || RO3G_08515.1
Complex I, Ndufs6	Uma	UM05775.1
Complex I, Ndufs6	Ncr	(NCU00484.2)
Complex I, Ndufs6	Pch	Phchr144349
Complex I, Ndufs6	Ppl	Pospl191879 || Pospl192045
Complex I, Ndufs6	Ang	A2QEI3
Complex I, Ndufs6	Clu	CLUG_00538.1
Complex I, Ndufs7	Dha	Q6BMU5
Complex I, Ndufs7	Pst	Picst375518
Complex I, Ndufs7	Bde	BDEG_06109
Complex I, Ndufs7	Ctr	CTRG_05643.3
Complex I, Ndufs7	Sro	Sporo118414
Complex I, Ndufs7	Lel	LELG_03030
Complex I, Ndufs7	Mfi	Mycfi153102
Complex I, Ndufs7	Ncr	(NCU03953.2)
Complex I, Ndufs7	Fox	FOXG_05891 || FOXG_05196
Complex I, Ndufs7	Cgu	PGUG_02259.1
Complex I, Ndufs7	Nha	Necha282494 || Necha273438 || Necha260986
Complex I, Ndufs7	Fve	FVEG_03763 || FVEG_02937
Complex I, Ndufs7	Ure	UREG_05671.1
Complex I, Ndufs7	Pbl	Phybl134382
Complex I, Ndufs7	Afl	AFL2G_12186 || AFL2G_12470
Complex I, Ndufs7	Cim	CIMG_10031 || CIMG_04248
Complex I, Ndufs7	Nfi	NFIA_023570 || NFIA_110300
Complex I, Ndufs7	Cne	Q55SA0
Complex I, Ndufs7	Lbi	Lacbi1183558
Complex I, Ndufs7	Ror	RO3G_14040.1 || RO3G_13183.1
Complex I, Ndufs7	Ssc	SS1G_08165.1
Complex I, Ndufs7	Ath	P56756 || Q42577
Complex I, Ndufs7	Ppl	Pospl1113096 || Pospl136241
Complex I, Ndufs7	Clu	CLUG_05257.1
Complex I, Ndufs7	Cci	CC1G_09331.1
Complex I, Ndufs7	Cal	orf19.6794
Complex I, Ndufs7	Hca	HCAG_03991.1
Complex I, Ndufs7	Hsa	ENSP00000233627
Complex I, Ndufs7	Afu	Q4WNI4
Complex I, Ndufs7	Ani	XP_661901.1
Complex I, Ndufs7	Mgr	MGG_10805.5 || MGG_12387.5

Complex I, Ndufs7	Cgo	CHG05446.1 || CHG08662.1
Complex I, Ndufs7	Ang	A2QXG1 || A2QHJ1
Complex I, Ndufs7	Pgr	PGTG_00582
Complex I, Ndufs7	Yli	Q6C2Q1
Complex I, Ndufs7	Sno	SNU07271.1 || SNU05340.1
Complex I, Ndufs7	Gze	Q4I0C6 || Q4HXN2
Complex I, Ndufs7	Pch	Phchr1123993
Complex I, Ndufs7	Cdu	protCdu4682
Complex I, Ndufs7	Acl	ACLA_017500 || ACLA_045130
Complex I, Ndufs7	Uma	UM05902.1
Complex I, Ndufs7	Aor	Q2U0B9 || Q2TYL4 || Q2UG17
Complex I, Ndufs7	Tre	Trire258201
Complex I, Ndufs7	Ate	ATEG_06833.1
Complex I, Ndufs8	Nfi	NFIA_018080
Complex I, Ndufs8	Ssc	SS1G_11900.1
Complex I, Ndufs8	Ath	Q9FX83 || Q42599 || P56755
Complex I, Ndufs8	Fox	FOXG_04085
Complex I, Ndufs8	Acl	ACLA_027960
Complex I, Ndufs8	Pgr	PGTG_10555
Complex I, Ndufs8	Cgu	PGUG_05115.1
Complex I, Ndufs8	Pan	protPan12416
Complex I, Ndufs8	Cal	orf19.4758
Complex I, Ndufs8	Ate	ATEG_02961.1
Complex I, Ndufs8	Ang	A2RB65
Complex I, Ndufs8	Bde	BDEG_01320
Complex I, Ndufs8	Ppl	Pospl1113268 || Pospl1123934
Complex I, Ndufs8	Cci	CC1G_01338.1
Complex I, Ndufs8	Nha	Necha273954
Complex I, Ndufs8	Pbl	Phybl126043
Complex I, Ndufs8	Bci	BC1G_15267.1
Complex I, Ndufs8	Fve	FVEG_07199
Complex I, Ndufs8	Tre	Trire262219
Complex I, Ndufs8	Pca	protPca3605
Complex I, Ndufs8	Cdu	protCdu6201
Complex I, Ndufs8	Mfi	Mycfi186428
Complex I, Ndufs8	Ctr	CTRG_03245.3
Complex I, Ndufs8	Hca	HCAG_05509.1
Complex I, Ndufs8	Pch	Phchr1129168
Complex I, Ndufs8	Dha	Q6BT75
Complex I, Ndufs8	Pst	Picst388630
Complex I, Ndufs8	Cim	CIMG_03996
Complex I, Ndufs8	Hsa	ENSP00000315774
Complex I, Ndufs8	Cne	Q55MA6
Complex I, Ndufs8	Cgo	CHG00047.1
Complex I, Ndufs8	Clu	CLUG_01879.1
Complex I, Ndufs8	Afl	AFL2G_09106
Complex I, Ndufs8	Lel	LELG_00319
Complex I, Ndufs8	Yli	Q9UUT8
Complex I, Ndufs8	Ure	UREG_01972.1
Complex I, Ndufs8	Sro	Sporo15762
Complex I, Ndufs8	Ncr	(NCU05009.2)
Complex I, Ndufs8	Afu	Q4WJB0
Complex I, Ndufs8	Ror	RO3G_05089.1 || RO3G_10997.1
Complex I, Ndufs8	Lbi	Lacbi1172170
Complex I, Ndufs8	Gze	Q4I4J1
Complex I, Ndufs8	Mgr	MGG_03483.5
Complex I, Ndufs8	Aor	Q2UN89
Complex I, Ndufs8	Sno	SNU02720.1
Complex I, Ndufv1	Sja	SJAG_04639
Complex I, Ndufv1	Acl	ACLA_050150
Complex I, Ndufv1	Afl	AFL2G_01924
Complex I, Ndufv1	Pbl	Phybl176222
Complex I, Ndufv1	Cgo	CHG09047.1
Complex I, Ndufv1	Yli	Q9UUU2
Complex I, Ndufv1	Mfi	Mycfi159995
Complex I, Ndufv1	Nha	Necha266841
Complex I, Ndufv1	Pst	Picst331688
Complex I, Ndufv1	Cne	Q55U82
Complex I, Ndufv1	Ror	RO3G_00533.1 || RO3G_16215.1
Complex I, Ndufv1	Ani	XP_663233.1
Complex I, Ndufv1	Gze	Q4I0K8
Complex I, Ndufv1	Ure	UREG_05077.1
Complex I, Ndufv1	Ang	A2QJ32
Complex I, Ndufv1	Sro	Sporo113825
Complex I, Ndufv1	Pch	Phchr14087
Complex I, Ndufv1	Bci	BC1G_11444.1
Complex I, Ndufv1	Lel	LELG_01962
Complex I, Ndufv1	Hca	HCAG_01603.1
Complex I, Ndufv1	Ate	ATEG_03973.1

Complex I, Ndufv1	Pgr	PGTG_07805
Complex I, Ndufv1	Clu	CLUG_02615.1
Complex I, Ndufv1	Pan	protPan8794
Complex I, Ndufv1	Spb	O94500
Complex I, Ndufv1	Tre	Trire265559
Complex I, Ndufv1	Ath	Q9FNN5
Complex I, Ndufv1	Fve	FVEG_03859
Complex I, Ndufv1	Sno	SNU06231.1
Complex I, Ndufv1	Afu	Q4WPZ9
Complex I, Ndufv1	Cal	orf19.4495
Complex I, Ndufv1	Ppl	Pospl1128103 || Pospl1121188
Complex I, Ndufv1	Ssc	SS1G_11651.1
Complex I, Ndufv1	Lbi	Lacbi1175119
Complex I, Ndufv1	Dha	Q6BM47
Complex I, Ndufv1	Fox	FOXG_05992
Complex I, Ndufv1	Cgu	PGUG_01472.1
Complex I, Ndufv1	Cim	CIMG_01512
Complex I, Ndufv1	Cci	CC1G_03104.1
Complex I, Ndufv1	Bde	BDEG_04083
Complex I, Ndufv1	Ctr	CTRG_01603.3
Complex I, Ndufv1	Ncr	(NCU04044.2)
Complex I, Ndufv1	Hsa	ENSP00000322450
Complex I, Ndufv1	Mgr	MGG_00647.5
Complex I, Ndufv1	Nfi	NFIA_104990
Complex I, Ndufv1	Cdu	protCdu1587
Complex I, Ndufv1	Uma	UM00718.1
Complex I, Ndufv1	Aor	Q2UJQ8
Complex I, Ndufv2	Cim	CIMG_01025
Complex I, Ndufv2	Hsa	ENSP00000343430 || ENSP00000327268
Complex I, Ndufv2	Afl	AFL2G_05502
Complex I, Ndufv2	Aor	Q2TZN8
Complex I, Ndufv2	Ppl	Pospl1111385 || Pospl1125235
Complex I, Ndufv2	Cal	orf19.2091
Complex I, Ndufv2	Fve	FVEG_02986
Complex I, Ndufv2	Pst	Picst376559
Complex I, Ndufv2	Dha	Q6BYU8
Complex I, Ndufv2	Pan	protPan7079
Complex I, Ndufv2	Gze	Q4HXS7
Complex I, Ndufv2	Pbl	Phybl139616
Complex I, Ndufv2	Sja	SJAG_00217
Complex I, Ndufv2	Ctr	CTRG_01110.3
Complex I, Ndufv2	Tre	Trire25387
Complex I, Ndufv2	Nfi	NFIA_084660
Complex I, Ndufv2	Cgo	CHG00684.1
Complex I, Ndufv2	Afu	Q4X1Q6
Complex I, Ndufv2	Ang	A2QZF9
Complex I, Ndufv2	Ror	RO3G_03903.1 || RO3G_09707.1
Complex I, Ndufv2	Lbi	Lacbi1306196
Complex I, Ndufv2	Yli	Q9UUT9
Complex I, Ndufv2	Mfi	Mycfi149175
Complex I, Ndufv2	Hca	HCAG_03687.1
Complex I, Ndufv2	Bci	BC1G_09132.1
Complex I, Ndufv2	Sro	Sporo116288
Complex I, Ndufv2	Cdu	protCdu1782
Complex I, Ndufv2	Ate	ATEG_09864.1
Complex I, Ndufv2	Pgr	PGTG_19779
Complex I, Ndufv2	Cgu	PGUG_02076.1
Complex I, Ndufv2	Fox	FOXG_05147
Complex I, Ndufv2	Ssc	SS1G_04889.1
Complex I, Ndufv2	Cci	CC1G_03815.1
Complex I, Ndufv2	Pch	Phchr1133066
Complex I, Ndufv2	Ath	O22769
Complex I, Ndufv2	Mgr	MGG_07301.5
Complex I, Ndufv2	Nha	Necha261324
Complex I, Ndufv2	Ani	XP_663681.1
Complex I, Ndufv2	Cne	Q55WY1
Complex I, Ndufv2	Uma	UM00634.1
Complex I, Ndufv2	Bde	BDEG_02641
Complex I, Ndufv2	Acl	ACLA_080940
Complex I, Ndufv2	Sno	SNU03113.1
Complex I, Ndufv2	Ncr	(NCU01169.2)
Complex I, Ndufv2	Spb	O13691
Complex I, Ndufv2	Ure	UREG_00971.1
Complex I, Ndufv2	Lel	LELG_01204
Complex I, Ndufv2	Clu	CLUG_00786.1
Complex I, NI9M	Pgr	PGTG_01821
Complex I, NI9M	Afu	Q4WUM5
Complex I, NI9M	Ssc	SS1G_03309.1
Complex I, NI9M	Fox	FOXG_01287

Complex I, NI9M	Bci	BC1G_09612.1
Complex I, NI9M	Mfi	Mycfi141375
Complex I, NI9M	Lel	LELG_04488
Complex I, NI9M	Ani	XP_662286.1
Complex I, NI9M	Ror	RO3G_14398.1
Complex I, NI9M	Ate	ATEG_06569.1
Complex I, NI9M	Lbi	Lacbi1297246
Complex I, NI9M	Ppl	Pospl147099 || Pospl156614
Complex I, NI9M	Hca	HCAG_00670.1
Complex I, NI9M	Uma	UM03545.1
Complex I, NI9M	Cim	CIMG_06717
Complex I, NI9M	Pch	Phchr1121802
Complex I, NI9M	Nha	Necha2102689
Complex I, NI9M	Sno	SNU14908.1
Complex I, NI9M	Ncr	(NCU00670.2)
Complex I, NI9M	Afl	AFL2G_10856
Complex I, NI9M	Tre	Trire266835
Complex I, NI9M	Gze	Q4IRG8
Complex I, NI9M	Fve	FVEG_00208
Complex I, NI9M	Nfi	NFIA_078320
Complex I, NI9M	Mgr	MGG_02822.5
Complex I, NI9M	Cal	orf19.3366.1
Complex I, NI9M	Acl	ACLA_011560
Complex I, NI9M	Ang	A2QN27
Complex I, NURM	Nha	Necha273392
Complex I, NURM	Fox	FOXG_00662
Complex I, NURM	Afu	Q4WCI3
Complex I, NURM	Ncr	(NCU00969.2)
Complex I, NURM	Mgr	MGG_10844.5
Complex I, NURM	Ure	UREG_01502.1
Complex I, NURM	Cgo	CHG05853.1
Complex I, NURM	Ani	XP_659048.1
Complex I, NURM	Cim	CIMG_02216
Complex I, NURM	Pan	protPan9107
Complex I, NURM	Mfi	Mycfi157017
Complex I, NURM	Tre	Trire2120836
Complex I, NURM	Gze	Q4IQW5
Complex I, NURM	Bci	BC1G_08025.1
Complex I, NURM	Ate	ATEG_00044.1
Complex I, NURM	Nfi	NFIA_097050
Complex I, NURM	Sno	SNU04115.1
Complex I, NURM	Ang	A2R8Y0
Complex I, NURM	Afl	AFL2G_12309
Complex I, NURM	Ssc	SS1G_01706.1
Complex I, NURM	Acl	ACLA_057670
Complex I, NURM	Hca	HCAG_02708.1
Complex I, NUVM	Sno	SNU01867.1
Complex I, NUVM	Bci	BC1G_15217.1
Complex I, NUVM	Ncr	(NCU00160.2)
Complex I, NUVM	Acl	ACLA_018500
Complex I, NUVM	Afl	AFL2G_00946
Complex I, NUVM	Nfi	NFIA_008370
Complex I, NUVM	Cgo	CHG02949.1
Complex I, NUVM	Mfi	Mycfi139441
Complex I, NUVM	Mgr	MGG_05679.5
Complex I, NUVM	Hca	HCAG_02130.1
Complex I, NUVM	Ani	XP_658234.1
Complex I, NUVM	Ang	A2QA15
Complex I, NUVM	Fve	FVEG_09211
Complex I, NUVM	Nha	Necha273768
Complex I, NUVM	Tre	Trire261577
Complex I, NUVM	Ate	ATEG_05094.1
Complex I, NUVM	Ssc	SS1G_07318.1
Complex I, NUVM	Gze	Q4IJS3
Complex I, NUWM	Cgu	PGUG_00736.1
Complex I, NUWM	Cdu	protCdu1688
Complex I, NUWM	Cal	orf19.1549
Complex I, NUWM	Ctr	CTRG_01380.3
Complex I, NUWM	Dha	Q6BWY4
Complex I, NUWM	Pst	Picst367718
Complex I, NUWM	Yli	Q6ZY23
Complex I, NUWM	Lel	LELG_00745
Complex I, NUWM	Clu	CLUG_00129.1
Complex I, NUXM	Uma	UM04606.1
Complex I, NUXM	Nha	Necha229145
Complex I, NUXM	Tre	Trire2121172
Complex I, NUXM	Pgr	PGTG_02978
Complex I, NUXM	Ppl	Pospl1120214
Complex I, NUXM	Cim	CIMG_08514

Complex I, NUXM	Cci	CC1G_00995.1
Complex I, NUXM	Lel	LELG_05306
Complex I, NUXM	Ncr	(NCU01859.2)
Complex I, NUXM	Ang	A2R2A5
Complex I, NUXM	Ure	UREG_02579.1
Complex I, NUXM	Pst	Picst375097
Complex I, NUXM	Lbi	Lacbi1171564
Complex I, NUXM	Bci	BC1G_08906.1
Complex I, NUXM	Sro	Sporo134391
Complex I, NUXM	Cal	orf19.6607
Complex I, NUXM	Ate	ATEG_02769.1
Complex I, NUXM	Cgu	PGUG_02853.1
Complex I, NUXM	Sno	SNU08252.1
Complex I, NUXM	Mgr	MGG_09006.5
Complex I, NUXM	Pca	protPca1030
Complex I, NUXM	Pch	Phchr1137777
Complex I, NUXM	Afl	AFL2G_12462
Complex I, NUXM	Pan	protPan2823
Complex I, NUXM	Dha	Q6BIW8
Complex I, NUXM	Fve	FVEG_13085
Complex I, NUXM	Ctr	CTRG_05898.3
Complex I, NUXM	Ani	XP_664580.1
Complex I, NUXM	Gze	Q4HTT9
Complex I, NUXM	Ssc	SS1G_11454.1
Complex I, NUXM	Acl	ACLA_053580
Complex I, NUXM	Hca	HCAG_07666.1
Complex I, NUXM	Cne	Q55NY7
Complex I, NUXM	Fox	FOXG_15623
Complex I, NUXM	Nfi	NFIA_028570
Complex I, NUXM	Aor	Q2PIY6
Complex I, NUXM	Cdu	protCdu6541
Complex I, NUXM	Mfi	Mycfi161110
Complex I, NUXM	Ror	RO3G_16026.1 || RO3G_15697.1
Complex I, NUXM	Pbl	Phybl140132
Complex I, NUXM	Cgo	CHG06701.1
Complex I, NUXM	Yli	Q6C4A6
Complex I, NUXM	Clu	CLUG_04147.1
Complex I, NUXM	Afu	Q4W9J0
Complex I, NUZM	Cci	CC1G_09310.1
Complex I, NUZM	Acl	ACLA_045180
Complex I, NUZM	Ppl	Pospl1114115 || Pospl1122846
Complex I, NUZM	Mgr	MGG_07504.5
Complex I, NUZM	Ani	XP_661904.1
Complex I, NUZM	Hca	HCAG_07459.1
Complex I, NUZM	Afl	AFL2G_04809 || AFL2G_12588
Complex I, NUZM	Yli	Q6CI10
Complex I, NUZM	Nha	Necha293762
Complex I, NUZM	Nfi	NFIA_110250
Complex I, NUZM	Ror	RO3G_13586.1
Complex I, NUZM	Sno	SNU05284.1
Complex I, NUZM	Lel	LELG_02113
Complex I, NUZM	Cim	CIMG_00967
Complex I, NUZM	Tre	Trire270439
Complex I, NUZM	Pca	protPca320
Complex I, NUZM	Fve	FVEG_03027
Complex I, NUZM	Cal	orf19.287
Complex I, NUZM	Lbi	Lacbi1324030
Complex I, NUZM	Aor	Q2UG23
Complex I, NUZM	Ncr	(NCU08930.2)
Complex I, NUZM	Cgo	CHG07000.1
Complex I, NUZM	Uma	UM06272.1
Complex I, NUZM	Clu	CLUG_03526.1
Complex I, NUZM	Ang	A2QHJ6
Complex I, NUZM	Ssc	SS1G_03728.1
Complex I, NUZM	Ate	ATEG_06829.1
Complex I, NUZM	Gze	Q4HXL1
Complex I, NUZM	Fox	FOXG_05109
Complex I, NUZM	Dha	Q6BJN9
Complex I, NUZM	Cne	Q55I84
Complex I, NUZM	Pbl	Phybl176616
Complex I, NUZM	Pan	protPan10611
Complex I, NUZM	Bci	BC1G_07041.1
Complex I, NUZM	Ctr	CTRG_02577.3
Complex I, NUZM	Pst	Picst372822
Complex I, NUZM	Cgu	PGUG_04641.1
Complex I, NUZM	Ure	UREG_00919.1
Complex I, NUZM	Cdu	protCdu4840
Complex I, NUZM	Sro	Sporo124329
Complex I, NUZM	Afu	A4D9N7

Complex II, SDHA	Ago	NP_983455.1 || NP_985914.1
Complex II, SDHA	Cne	Q55KJ7 || Q55MX1
Complex II, SDHA	Ath	Q94AY1 || Q9LER1 || O82663 || Q9ZPX5
Complex II, SDHA	Sca	Scas_678.4 || Scas_608.10 || Scas_709.19
Complex II, SDHA	Pbl	Phybl139766 || Phybl135969
Complex II, SDHA	Pch	Phchr1123932 || Phchr1132705
Complex II, SDHA	Sja	SJAG_00203 || SJAG_01671
Complex II, SDHA	Cci	CC1G_02036.1 || CC1G_06084.1
Complex II, SDHA	Cim	CIMG_08720 || CIMG_02339 || CIMG_06614
Complex II, SDHA	Aor	Q2US30 || Q2U4B1
Complex II, SDHA	Tre	Trire281303 || Trire2121019
Complex II, SDHA	Sku	protSku5931 || protSku584 || protSku3511 || protSku3605 || protSku1318
Complex II, SDHA	Lbi	Lacbi1317302 || Lacbi1184667 || Lacbi1188373 || Lacbi1317296 || Lacbi1247971 || Lacbi1248051 || Lacbi1142430
Complex II, SDHA	Afl	AFL2G_00589 || AFL2G_10906
Complex II, SDHA	Ani	XP_659147.1 || XP_660520.1
Complex II, SDHA	Fox	FOXG_05885 || FOXG_01544
Complex II, SDHA	Cdu	protCdu6423 || protCdu777
Complex II, SDHA	Sno	SNU04712.1 || SNU16546.1 || SNU07394.1
Complex II, SDHA	Spb	O13755 || Q9UTJ7
Complex II, SDHA	Sro	Sporo119407
Complex II, SDHA	Acl	ACLA_058950 || ACLA_035740
Complex II, SDHA	Nfi	NFIA_098260 || NFIA_069350
Complex II, SDHA	Skl	protSkl684 || protSkl2330 || protSkl4408
Complex II, SDHA	Spa	protSpa2880 || protSpa3460 || protSpa4622 || protSpa2579
Complex II, SDHA	Smi	protSmi4571 || protSmi2557 || protSmi4851
Complex II, SDHA	Ncr	(NCU02580.2) || (NCU08336.2)
Complex II, SDHA	Kpo	Kpol_543.66
Complex II, SDHA	Cgo	CHG01384.1 || CHG01641.1
Complex II, SDHA	Gze	Q4I0B7 || Q4I5Z7
Complex II, SDHA	Ctr	CTRG_01536.3 || CTRG_03629.3 || CTRG_04515.3 || CTRG_03795.3
Complex II, SDHA	Clu	CLUG_03242.1 || CLUG_00494.1
Complex II, SDHA	Pgr	PGTG_15327 || PGTG_17753 || PGTG_10848
Complex II, SDHA	Afu	Q4WC67 || Q4WX09
Complex II, SDHA	Bci	BC1G_16089.1 || BC1G_07795.1
Complex II, SDHA	Ror	RO3G_10913.1 || RO3G_02171.1 || RO3G_07288.1 || RO3G_13321.1
Complex II, SDHA	Ang	A2R8H3 || A2QEZ7 || A2QDM2
Complex II, SDHA	Uma	UM04971.1 || UM01172.1
Complex II, SDHA	Sce	YKL148C || YJL045W || YEL047C
Complex II, SDHA	Bde	BDEG_06945
Complex II, SDHA	Hca	HCAG_03323.1 || HCAG_06317.1
Complex II, SDHA	Mfi	Mycfi187391 || Mycfi154991 || Mycfi187948
Complex II, SDHA	Ppl	Pospl1112323 || Pospl1114981 || Pospl1118125 || Pospl1108338
Complex II, SDHA	Yli	Q6C9G6
Complex II, SDHA	Cgl	CAGL0L01177g || CAGL0J00847g || CAGL0I01320g
Complex II, SDHA	Kwa	Kwal_23.5461 || Kwal_27.10088
Complex II, SDHA	Hsa	ENSP00000264932 || ENSP00000347017 || ENSP00000336646
Complex II, SDHA	Cal	orf19.440 || orf19.5005 || orf19.2871
Complex II, SDHA	Lel	LELG_03552 || LELG_01770 || LELG_05728
Complex II, SDHA	Ate	ATEG_07743.1 || ATEG_01694.1
Complex II, SDHA	Ure	UREG_02695.1 || UREG_01411.1 || UREG_03831.1
Complex II, SDHA	Cgu	PGUG_05599.1 || PGUG_05465.1
Complex II, SDHA	Pst	Picst353948 || Picst366251
Complex II, SDHA	Fve	FVEG_03757 || FVEG_07903
Complex II, SDHA	Ssc	SS1G_06222.1 || SS1G_07864.1
Complex II, SDHA	Pan	protPan12747 || protPan6436
Complex II, SDHA	Mgr	MGG_03619.5 || MGG_00168.5
Complex II, SDHA	Dha	Q6BSW6 || Q6BQ26 || Q6BQ85
Complex II, SDHA	Kla	XP_453260.1
Complex II, SDHA	Sba	Sbay_53.69 || Sbay_51.139 || Sbay_22.34 || Sbay_51.53
Complex II, SDHA	Nha	Necha265946 || Necha268374
Complex II, SDHB	Ani	XP_659936.1
Complex II, SDHB	Ppl	Pospl1107653
Complex II, SDHB	Pst	Picst350416
Complex II, SDHB	Cgl	CAGL0C03223g || CAGL0E03850g
Complex II, SDHB	Lel	LELG_03274
Complex II, SDHB	Sca	Scas_622.3 || Scas_400.3
Complex II, SDHB	Pch	Phchr17113
Complex II, SDHB	Mgr	MGG_00167.5
Complex II, SDHB	Skl	protSkl3747
Complex II, SDHB	Kla	XP_453977.1
Complex II, SDHB	Ssc	SS1G_04384.1
Complex II, SDHB	Tre	Trire2123198
Complex II, SDHB	Mfi	Mycfi151773
Complex II, SDHB	Hca	HCAG_03263.1
Complex II, SDHB	Afu	Q4WV09
Complex II, SDHB	Sba	Sbay_56.14
Complex II, SDHB	Bci	BC1G_13286.1
Complex II, SDHB	Ath	Q9FM32 || Q9LTZ2 || Q9FJP9
Complex II, SDHB	Uma	UM00844.1

Complex II, SDHB	Ror	RO3G_03816.1 || RO3G_04752.1
Complex II, SDHB	Ate	ATEG_07592.1
Complex II, SDHB	Nfi	NFIA_076860
Complex II, SDHB	Sce	YLL041C
Complex II, SDHB	Ago	NP_983339.1
Complex II, SDHB	Kpo	Kpol_388.3 || Kpol_1070.4
Complex II, SDHB	Afl	AFL2G_11667
Complex II, SDHB	Sja	SJAG_00260
Complex II, SDHB	Pan	protPan6391
Complex II, SDHB	Sno	SNU03351.1
Complex II, SDHB	Aor	Q2TWM0
Complex II, SDHB	Cdu	protCdu2528
Complex II, SDHB	Yli	Q6C823
Complex II, SDHB	Fox	FOXG_09278
Complex II, SDHB	Cgo	CHG01392.1
Complex II, SDHB	Ncr	(NCU00959.2)
Complex II, SDHB	Gze	Q4IAZ8
Complex II, SDHB	Cim	CIMG_05754
Complex II, SDHB	Spb	P21911
Complex II, SDHB	Cgu	PGUG_03289.1
Complex II, SDHB	Nha	Necha295465
Complex II, SDHB	Dha	Q6BHD9
Complex II, SDHB	Acl	ACLA_013460
Complex II, SDHB	Pbl	Phybl121052
Complex II, SDHB	Clu	CLUG_04005.1
Complex II, SDHB	Sro	Sporo19115
Complex II, SDHB	Lbi	Lacbi1182791
Complex II, SDHB	Ang	A2R3I4
Complex II, SDHB	Fve	FVEG_06879
Complex II, SDHB	Cci	CC1G_09250.1
Complex II, SDHB	Ctr	CTRG_00535.3
Complex II, SDHB	Pca	protPca1660
Complex II, SDHB	Cne	Q55PT5
Complex II, SDHB	Cal	orf19.637
Complex II, SDHB	Ure	UREG_04544.1
Complex II, SDHB	Hsa	ENSP00000235768
Complex II, SDHB	Bde	BDEG_01197
Complex II, SDHB	Kwa	Kwal_55.20580
Complex II, SDHB	Smi	protSmi379
Complex II, SDHB	Spa	protSpa4910
Complex II, SDHC	Gze	Q4I196 || Q4ILC7
Complex II, SDHC	Pbl	Phybl169734
Complex II, SDHC	Ago	NP_985754.1
Complex II, SDHC	Cci	CC1G_06197.1
Complex II, SDHC	Nfi	NFIA_001520 || NFIA_077630
Complex II, SDHC	Kla	XP_454496.1
Complex II, SDHC	Fve	FVEG_06680 || FVEG_07431
Complex II, SDHC	Sno	SNU11157.1
Complex II, SDHC	Ncr	(NCU07756.2)
Complex II, SDHC	Lbi	Lacbi1189604 || Lacbi1295137
Complex II, SDHC	Spb	O74882
Complex II, SDHC	Cne	Q560A0
Complex II, SDHC	Kpo	Kpol_1018.147
Complex II, SDHC	Spa	protSpa783 || protSpa2872
Complex II, SDHC	Sro	Sporo110194
Complex II, SDHC	Yli	Q6C450
Complex II, SDHC	Bci	BC1G_14785.1
Complex II, SDHC	Aor	Q2U3V9
Complex II, SDHC	Ate	ATEG_06343.1 || ATEG_06480.1
Complex II, SDHC	Sja	SJAG_03920
Complex II, SDHC	Afu	Q4WUU3
Complex II, SDHC	Cgo	CHG07664.1
Complex II, SDHC	Fox	FOXG_09077 || FOXG_04307
Complex II, SDHC	Ani	XP_682062.1
Complex II, SDHC	Nha	Necha270858 || Necha250762
Complex II, SDHC	Mfi	Mycfi17820 || Mycfi172469
Complex II, SDHC	Sca	Scas_721.108
Complex II, SDHC	Ssc	SS1G_01661.1
Complex II, SDHC	Pch	Phchr143468
Complex II, SDHC	Kwa	Kwal_26.7549
Complex II, SDHC	Mgr	MGG_04876.5
Complex II, SDHC	Cgl	CAGL0D01958g
Complex II, SDHC	Afl	AFL2G_10745
Complex II, SDHC	Sku	protSku4742 || protSku2813
Complex II, SDHC	Skl	protSkl3946
Complex II, SDHC	Smi	protSmi39 || protSmi4580
Complex II, SDHC	Sba	Sbay_67.49
Complex II, SDHC	Hsa	ENSP00000271481
Complex II, SDHC	Dha	Q6BXB2

Complex II, SDHC	Ure	UREG_03828.1 || UREG_07057.1
Complex II, SDHC	Cim	CIMG_06617 || CIMG_03201
Complex II, SDHC	Pan	protPan5396
Complex II, SDHC	Pst	Picst335527
Complex II, SDHC	Uma	UM03999.1
Complex II, SDHC	Cal	orf19.1480
Complex II, SDHC	Ang	A2QMS7
Complex II, SDHC	Ppl	Pospl1116499 || Pospl1109390
Complex II, SDHC	Lel	LELG_00930
Complex II, SDHC	Acl	ACLA_010860
Complex II, SDHC	Ctr	CTRG_01263.3
Complex II, SDHC	Tre	Trire260089
Complex II, SDHC	Pgr	PGTG_02673
Complex II, SDHC	Sce	YMR118C || YKL141W
Complex II, SDHC	Hca	HCAG_00596.1 || HCAG_03426.1
Complex II, SDHC	Ror	RO3G_03148.1
Complex II, SDHC	Clu	CLUG_03097.1
Complex II, SDHC	Cdu	protCdu1889
Complex II, SDHD	Pan	protPan7496
Complex II, SDHD	Hca	HCAG_02344.1
Complex II, SDHD	Cgl	CAGL0A03784g || CAGL0F05863g
Complex II, SDHD	Pst	Picst335330 || Picst390476
Complex II, SDHD	Kwa	Kwal_55.20296 || Kwal_23.3343
Complex II, SDHD	Cne	Q55X71
Complex II, SDHD	Fve	FVEG_00440
Complex II, SDHD	Spb	Q9P7X0
Complex II, SDHD	Sku	protSku628 || protSku82
Complex II, SDHD	Cgu	PGUG_00504.1 || PGUG_04469.1
Complex II, SDHD	Pca	protPca544
Complex II, SDHD	Fox	FOXG_01074
Complex II, SDHD	Skl	protSkl5088
Complex II, SDHD	Cim	CIMG_03134
Complex II, SDHD	Bci	BC1G_01333.1
Complex II, SDHD	Cdu	protCdu1227 || protCdu394
Complex II, SDHD	Mfi	Mycfi150482
Complex II, SDHD	Clu	CLUG_00636.1 || CLUG_03577.1
Complex II, SDHD	Afl	AFL2G_01091
Complex II, SDHD	Sro	Sporo130628
Complex II, SDHD	Afu	Q4WRP4
Complex II, SDHD	Sno	SNU11620.1
Complex II, SDHD	Pgr	PGTG_10911
Complex II, SDHD	Sce	YOR297C || YLR164W || YDR178W
Complex II, SDHD	Nfi	NFIA_009730
Complex II, SDHD	Ror	RO3G_02004.1 || RO3G_05559.1
Complex II, SDHD	Gze	Q4IPW5
Complex II, SDHD	Cal	orf19.4022 || orf19.4468
Complex II, SDHD	Lel	LELG_04095 || LELG_01522
Complex II, SDHD	Ssc	SS1G_06173.1
Complex II, SDHD	Ang	A2QB53
Complex II, SDHD	Ani	XP_658500.1
Complex II, SDHD	Ppl	Pospl157627 || Pospl1128844 || Pospl1130336
Complex II, SDHD	Lbi	Lacbi1249103
Complex II, SDHD	Pch	Phchr12221
Complex II, SDHD	Bde	BDEG_01106
Complex II, SDHD	Sca	Scas_665.22 || Scas_682.9
Complex II, SDHD	Mgr	MGG_00666.5
Complex II, SDHD	Acl	ACLA_019770
Complex II, SDHD	Cgo	CHG01750.1
Complex II, SDHD	Pbl	Phybl122059 || Phybl117694
Complex II, SDHD	Uma	UM03845.1
Complex II, SDHD	Nha	Necha2102395
Complex II, SDHD	Yli	Q6CGZ0
Complex II, SDHD	Ncr	(NCU03031.2)
Complex II, SDHD	Ure	UREG_02833.1
Complex II, SDHD	Sba	Sbay_87.4 || Sbay_62.2 || Sbay_19.4
Complex II, SDHD	Tre	Trire222707
Complex II, SDHD	Ctr	CTRG_06134.3 || CTRG_04772.3
Complex II, SDHD	Kpo	Kpol_1030.24 || Kpol_1024.21
Complex II, SDHD	Ago	NP_986070.1 || NP_986530.1
Complex II, SDHD	Kla	XP_456198.1 || XP_453862.1
Complex II, SDHD	Cci	CC1G_02650.1
Complex II, SDHD	Ate	ATEG_05235.1
Complex II, SDHD	Sja	SJAG_02398
Complex II, SDHD	Dha	Q6BRT5 || Q6BHT6
Complex II, SDHD	Spa	protSpa1972 || protSpa371 || protSpa203
Complex II, SDHD	Smi	protSmi3366 || protSmi4919
Complex III, Cytb	Sja	SJAG_mit1
Complex III, Cytb	Yli	Q9B6C9 || Q9B6D0
Complex III, Cytb	Hsa	ENSP00000354554

Complex III, Cytb	Pan	protPan12890
Complex III, Cytb	Sce	Q0110 || Q0115 || Q0120 || Q0105
Complex III, Cytb	Ago	NP_987085.1
Complex III, Cytb	Spb	P05501
Complex III, Cytb	Uma	UM_mit21
Complex III, Cytb	Ncr	Neur.crassa.mt1
Complex III, Cytb	Ath	P42792 || P56773
Complex III, Cytb	Cgu	PGUG_05919.1
Complex III, Cytb	Kla	YP_054497.1
Complex III, Cytc1	Ang	A2Q903
Complex III, Cytc1	Sca	Scas_698.35 || Scas_126.1
Complex III, Cytc1	Ani	XP_657961.1
Complex III, Cytc1	Nfi	NFIA_022580
Complex III, Cytc1	Gze	Q4IQE7
Complex III, Cytc1	Ppl	Pospl1124230 || Pospl1108315
Complex III, Cytc1	Fox	FOXG_00494
Complex III, Cytc1	Nha	Necha298806
Complex III, Cytc1	Kwa	Kwal_55.21731
Complex III, Cytc1	Lel	LELG_00961
Complex III, Cytc1	Clu	CLUG_02783.1
Complex III, Cytc1	Pgr	PGTG_02872
Complex III, Cytc1	Dha	Q6BV04
Complex III, Cytc1	Lbi	Lacbi1329524
Complex III, Cytc1	Acl	ACLA_032440
Complex III, Cytc1	Smi	protSmi5056
Complex III, Cytc1	Yli	Q6CGP7
Complex III, Cytc1	Cal	orf19.3527
Complex III, Cytc1	Sba	Sbay_86.63
Complex III, Cytc1	Mgr	MGG_13170.5
Complex III, Cytc1	Cdu	protCdu1569
Complex III, Cytc1	Cne	Q55QD2
Complex III, Cytc1	Tre	Trire273571
Complex III, Cytc1	Mfi	Mycfi186820
Complex III, Cytc1	Bci	BC1G_05275.1
Complex III, Cytc1	Bde	BDEG_01572
Complex III, Cytc1	Ure	UREG_06035.1
Complex III, Cytc1	Sku	protSku3034
Complex III, Cytc1	Cgo	CHG00755.1
Complex III, Cytc1	Aor	Q2URC0
Complex III, Cytc1	Spa	protSpa4703
Complex III, Cytc1	Cim	CIMG_09976
Complex III, Cytc1	Sro	Sporo118496
Complex III, Cytc1	Skl	protSkl4791
Complex III, Cytc1	Ate	ATEG_04885.1
Complex III, Cytc1	Afu	Q4WKL3
Complex III, Cytc1	Ncr	(NCU09816.2)
Complex III, Cytc1	Cgl	CAGL0L10406g
Complex III, Cytc1	Kpo	Kpol_489.18
Complex III, Cytc1	Cci	CC1G_05743.1
Complex III, Cytc1	Ctr	CTRG_01636.3
Complex III, Cytc1	Sno	SNU03340.1
Complex III, Cytc1	Ror	RO3G_08415.1 || RO3G_07753.1 || RO3G_00332.1
Complex III, Cytc1	Sja	SJAG_02646
Complex III, Cytc1	Pbl	Phybl116568
Complex III, Cytc1	Ath	Q9FKS5 || Q9LK29
Complex III, Cytc1	Fve	FVEG_01019
Complex III, Cytc1	Pch	Phchr1137302
Complex III, Cytc1	Pst	Picst386147
Complex III, Cytc1	Ago	NP_984304.1
Complex III, Cytc1	Pan	protPan12108
Complex III, Cytc1	Uma	UM04631.1
Complex III, Cytc1	Spb	O59680
Complex III, Cytc1	Cgu	PGUG_05346.1
Complex III, Cytc1	Sce	YOR065W
Complex III, Cytc1	Kla	XP_455824.1
Complex III, Cytc1	Hsa	ENSP00000317159
Complex III, Cytc1	Afl	AFL2G_00866
Complex III, Cytc1	Hca	HCAG_03273.1
Complex III, Cytc1	Ssc	SS1G_12792.1
Complex III, ISP	Smi	protSmi2206
Complex III, ISP	Lbi	Lacbi1321219
Complex III, ISP	Pst	Picst339828
Complex III, ISP	Ani	XP_659910.1
Complex III, ISP	Cci	CC1G_10506.1
Complex III, ISP	Pgr	PGTG_17500
Complex III, ISP	Ago	NP_984794.1
Complex III, ISP	Ath	Q94JS0 || Q9LYR3 || Q9LYR2
Complex III, ISP	Sja	SJAG_00601
Complex III, ISP	Cdu	protCdu5199

Complex III, ISP	Yli	Q6CI02
Complex III, ISP	Sca	Scas_583.10
Complex III, ISP	Sro	Sporo19817
Complex III, ISP	Cgu	PGUG_00871.1
Complex III, ISP	Ang	A2R3F2
Complex III, ISP	Cal	orf19.5893
Complex III, ISP	Bde	BDEG_07697
Complex III, ISP	Clu	CLUG_05494.1
Complex III, ISP	Pbl	Phybl140975 || Phybl139558
Complex III, ISP	Dha	Q6BLP8
Complex III, ISP	Pca	protPca3669
Complex III, ISP	Cgl	CAGL0I03190g
Complex III, ISP	Mfi	Mycfi172052
Complex III, ISP	Sce	YEL024W
Complex III, ISP	Acl	ACLA_013620
Complex III, ISP	Gze	Q4IC07
Complex III, ISP	Spb	Q09154
Complex III, ISP	Afu	Q4WV31
Complex III, ISP	Nha	Necha2103366
Complex III, ISP	Hca	HCAG_00943.1
Complex III, ISP	Ctr	CTRG_02304.3
Complex III, ISP	Cgo	CHG04202.1
Complex III, ISP	Cim	CIMG_00699
Complex III, ISP	Ppl	Pospl163690 || Pospl1116810 || Pospl1116049
Complex III, ISP	Kpo	Kpol_1056.22
Complex III, ISP	Fve	FVEG_06221
Complex III, ISP	Pch	Phchr1124677
Complex III, ISP	Cne	Q55SF1
Complex III, ISP	Sku	protSku3059
Complex III, ISP	Ssc	SS1G_08646.1
Complex III, ISP	Hsa	ENSP00000339059 || ENSP00000306397 || ENSP00000303001
Complex III, ISP	Pan	protPan527
Complex III, ISP	Afl	AFL2G_11639
Complex III, ISP	Bci	BC1G_07481.1
Complex III, ISP	Aor	Q2TWP6
Complex III, ISP	Mgr	MGG_05093.5
Complex III, ISP	Kwa	Kwal_34.16074
Complex III, ISP	Sba	Sbay_22.55
Complex III, ISP	Ate	ATEG_07615.1
Complex III, ISP	Tre	Trire221609
Complex III, ISP	Sno	SNU08842.1
Complex III, ISP	Ncr	(NCU06606.2)
Complex III, ISP	Lel	LELG_02655
Complex III, ISP	Fox	FOXG_08455
Complex III, ISP	Spa	protSpa3431
Complex III, ISP	Ure	UREG_00690.1
Complex III, ISP	Nfi	NFIA_076600
Complex III, ISP	Skl	protSkl5531
Complex III, ISP	Ror	RO3G_01417.1 || RO3G_11123.1 || RO3G_13836.1
Complex III, ISP	Kla	XP_454973.1
Complex III, ISP	Uma	UM03825.1
Complex III, QCR1	Smi	protSmi2406 || protSmi974
Complex III, QCR1	Fox	FOXG_00825
Complex III, QCR1	Bde	BDEG_01030 || BDEG_04879 || BDEG_06151
Complex III, QCR1	Aor	Q2UNG4 || Q2UCV1
Complex III, QCR1	Uma	UM02600.1 || UM05993.1
Complex III, QCR1	Pca	protPca3459
Complex III, QCR1	Nha	Necha265828
Complex III, QCR1	Clu	CLUG_04927.1 || CLUG_00643.1 || CLUG_03588.1
Complex III, QCR1	Lbi	Lacbi1191797 || Lacbi1320873 || Lacbi1292439
Complex III, QCR1	Hca	HCAG_02342.1
Complex III, QCR1	Ath	O04308 || Q9ZU25 || Q9SGA7
Complex III, QCR1	Ncr	(NCU02549.2)
Complex III, QCR1	Ate	ATEG_00386.1 || ATEG_00623.1
Complex III, QCR1	Ssc	SS1G_06172.1
Complex III, QCR1	Pgr	PGTG_17063 || PGTG_00050
Complex III, QCR1	Sca	Scas_688.21 || Scas_340.1 || Scas_691.43 || Scas_679.2
Complex III, QCR1	Pch	Phchr138204 || Phchr18574
Complex III, QCR1	Dha	Q6BNT0 || Q6BRV0 || Q6BHS1
Complex III, QCR1	Pst	Picst381500 || Picst336688 || Picst349260
Complex III, QCR1	Cim	CIMG_09062 || CIMG_03137
Complex III, QCR1	Afu	Q6MYU8 || Q4WS30
Complex III, QCR1	Sku	protSku3011 || protSku5161
Complex III, QCR1	Afl	AFL2G_07594 || AFL2G_03343
Complex III, QCR1	Pbl	Phybl133521 || Phybl138384 || Phybl178454 || Phybl138782 || Phybl179049
Complex III, QCR1	Cal	orf19.3026 || orf19.6295 || orf19.4016
Complex III, QCR1	Ang	A2QAN9
Complex III, QCR1	Spb	O94745 || Q9P7X1
Complex III, QCR1	Cgu	PGUG_04481.1 || PGUG_03412.1 || PGUG_00501.1

Complex III, QCR1	Fve	FVEG_00620
Complex III, QCR1	Kwa	Kwal_55.19854 || Kwal_23.5543 || Kwal_23.3345
Complex III, QCR1	Cne	Q55RR9 || Q55P20
Complex III, QCR1	Bci	BC1G_01999.1 || BC1G_01334.1
Complex III, QCR1	Pan	protPan6075 || protPan10279
Complex III, QCR1	Cci	CC1G_06569.1 || CC1G_05009.1
Complex III, QCR1	Spa	protSpa4550 || protSpa3589 || protSpa390
Complex III, QCR1	Skl	protSkl4672 || protSkl5091 || protSkl1451
Complex III, QCR1	Tre	Trire2122703
Complex III, QCR1	Mfi	Mycfi187169
Complex III, QCR1	Sja	SJAG_04641 || SJAG_02397
Complex III, QCR1	Ror	RO3G_04355.1 || RO3G_03819.1 || RO3G_04336.1 || RO3G_01811.1 || RO3G_00800.1
Complex III, QCR1	Ctr	CTRG_04798.3 || CTRG_05290.3 || CTRG_06129.3
Complex III, QCR1	Sro	Sporo11735 || Sporo127102
Complex III, QCR1	Sba	Sbay_41.56 || Sbay_4.95 || Sbay_62.1
Complex III, QCR1	Cgo	CHG03089.1 || CHG01367.1
Complex III, QCR1	Ago	NP_983471.1 || NP_986529.1 || NP_983093.1
Complex III, QCR1	Yli	Q6CGY9 || Q6C1U0
Complex III, QCR1	Cdu	protCdu49 || protCdu3211 || protCdu1231
Complex III, QCR1	Ani	XP_658708.1 || XP_658351.1
Complex III, QCR1	Sce	YBL045C || YHR024C || YLR163C
Complex III, QCR1	Hsa	ENSP00000268379 || ENSP00000298536 || ENSP00000249269 || ENSP00000203407
Complex III, QCR1	Ppl	Pospl1125932 || Pospl1124638
Complex III, QCR1	Kla	XP_453861.1 || XP_454472.1 || XP_454203.1
Complex III, QCR1	Cgl	CAGL0F04565g || CAGL0J00671g || CAGL0H02739g
Complex III, QCR1	Kpo	Kpol_1032.95 || Kpol_1013.73 || Kpol_1024.23
Complex III, QCR1	Nfi	NFIA_013660 || NFIA_011220
Complex III, QCR1	Sno	SNU11621.1
Complex III, QCR1	Acl	ACLA_023740 || ACLA_021220
Complex III, QCR1	Gze	Q4IJP5 || Q4IPJ5
Complex III, QCR1	Mgr	MGG_03600.5
Complex III, QCR1	Lel	LELG_01543 || LELG_03744 || LELG_03988
Complex III, QCR1	Ure	UREG_03324.1 || UREG_02827.1
Complex III, QCR10	Cal	orf19.2439.1
Complex III, QCR10	Skl	protSkl906
Complex III, QCR10	Kpo	Kpol_1018.43
Complex III, QCR10	Cgl	CAGL0K02893g
Complex III, QCR10	Ago	NP_986607.1
Complex III, QCR10	Pst	Picst352082
Complex III, QCR10	Yli	Q6CC60
Complex III, QCR10	Kla	XP_454575.1
Complex III, QCR10	Ctr	CTRG_04268.3
Complex III, QCR10	Dha	Q6BUS4
Complex III, QCR10	Sce	YHR001W-A
Complex III, QCR2	Pan	protPan10974 || protPan6075 || protPan10279
Complex III, QCR2	Pst	Picst378316 || Picst349260 || Picst336688
Complex III, QCR2	Pca	protPca3459
Complex III, QCR2	Cgo	CHG08239.1 || CHG03089.1 || CHG01367.1
Complex III, QCR2	Kpo	Kpol_1050.115 || Kpol_1032.95 || Kpol_1024.23
Complex III, QCR2	Gze	Q4IQ64 || Q4IJP5 || Q4IPJ5
Complex III, QCR2	Hca	HCAG_06996.1 || HCAG_02342.1
Complex III, QCR2	Kwa	Kwal_23.5419 || Kwal_23.5543
Complex III, QCR2	Sca	Scas_631.2 || Scas_688.21 || Scas_679.2
Complex III, QCR2	Ang	A2QUS8 || A2QQX8 || A2QAN9
Complex III, QCR2	Cgl	CAGL0G10131g || CAGL0J00671g || CAGL0H02739g
Complex III, QCR2	Sja	SJAG_00755 || SJAG_02397 || SJAG_04641
Complex III, QCR2	Sce	YPR191W || YHR024C || YLR163C
Complex III, QCR2	Lel	LELG_03842 || LELG_03744
Complex III, QCR2	Uma	UM01478.1 || UM02600.1
Complex III, QCR2	Acl	ACLA_000950 || ACLA_023740 || ACLA_021220
Complex III, QCR2	Ncr	(NCU03559.2) || (NCU06270.2) || (NCU02549.2)
Complex III, QCR2	Tre	Trire248883 || Trire2121890 || Trire2122703
Complex III, QCR2	Clu	CLUG_05174.1 || CLUG_04927.1 || CLUG_03588.1
Complex III, QCR2	Aor	Q2U9X6 || Q2UNG4 || Q2UCV1
Complex III, QCR2	Nha	Necha265760 || Necha294534 || Necha265828
Complex III, QCR2	Ppl	Pospl1116029 || Pospl1120716 || Pospl1125932 || Pospl1116542 || Pospl1124638
Complex III, QCR2	Ath	Q9ZU25 || O04308 || Q9SGA7
Complex III, QCR2	Yli	Q6C2E3 || Q6C1U0 || Q6CGY9
Complex III, QCR2	Ate	ATEG_07992.1 || ATEG_00386.1 || ATEG_00623.1
Complex III, QCR2	Kla	XP_451693.1 || XP_454472.1 || XP_453861.1
Complex III, QCR2	Nfi	NFIA_037890 || NFIA_013660 || NFIA_011220
Complex III, QCR2	Cci	CC1G_01555.1 || CC1G_06569.1 || CC1G_05009.1
Complex III, QCR2	Bde	BDEG_01030 || BDEG_04879
Complex III, QCR2	Cne	Q55LX5 || Q55RR9 || Q55P20
Complex III, QCR2	Fox	FOXG_00961 || FOXG_10485 || FOXG_00825
Complex III, QCR2	Afl	AFL2G_09997 || AFL2G_07594 || AFL2G_03343
Complex III, QCR2	Sku	protSku892 || protSku5161
Complex III, QCR2	Mgr	MGG_03226.5 || MGG_05258.5 || MGG_03600.5
Complex III, QCR2	Dha	Q6BPY6

Complex III, QCR2	Smi	protSmi2406
Complex III, QCR2	Ure	UREG_04088.1 || UREG_03324.1 || UREG_02827.1
Complex III, QCR2	Ssc	SS1G_01597.1 || SS1G_00522.1 || SS1G_06172.1
Complex III, QCR2	Bci	BC1G_02472.1 || BC1G_01999.1 || BC1G_01334.1
Complex III, QCR2	Ago	NP_983205.1 || NP_983471.1 || NP_986529.1
Complex III, QCR2	Cgu	PGUG_05422.1 || PGUG_04481.1
Complex III, QCR2	Sno	SNU01693.1 || SNU10235.1 || SNU11621.1
Complex III, QCR2	Hsa	ENSP00000268379 || ENSP00000249269
Complex III, QCR2	Cal	orf19.2644
Complex III, QCR2	Pbl	Phybl133521 || Phybl138384 || Phybl178454
Complex III, QCR2	Cim	CIMG_06296 || CIMG_09062 || CIMG_03137
Complex III, QCR2	Sro	Sporo110336 || Sporo11735
Complex III, QCR2	Afu	Q4WEU3 || Q6MYU8 || Q4WS30
Complex III, QCR2	Ctr	CTRG_02693.3 || CTRG_05290.3
Complex III, QCR2	Skl	protSkl2187 || protSkl5091 || protSkl1451
Complex III, QCR2	Sba	Sbay_96.5 || Sbay_41.56 || Sbay_62.1
Complex III, QCR2	Cdu	protCdu1112
Complex III, QCR2	Lbi	Lacbi1191797 || Lacbi1292439
Complex III, QCR2	Pch	Phchr129242 || Phchr138204 || Phchr18574
Complex III, QCR2	Ani	XP_681542.1 || XP_658708.1 || XP_658351.1
Complex III, QCR2	Mfi	Mycfi172474 || Mycfi12187 || Mycfi187169
Complex III, QCR2	Ror	RO3G_09327.1 || RO3G_04355.1 || RO3G_03819.1 || RO3G_04336.1
Complex III, QCR2	Spb	P78761 || O94745
Complex III, QCR2	Pgr	PGTG_00730 || PGTG_00050
Complex III, QCR2	Spa	protSpa5007 || protSpa3589 || protSpa390
Complex III, QCR2	Fve	FVEG_00552 || FVEG_09138 || FVEG_00620
Complex III, QCR6	Smi	protSmi2004 || protSmi2005
Complex III, QCR6	Sku	protSku1213
Complex III, QCR6	Cgl	CAGL0D05192g
Complex III, QCR6	Spa	protSpa4375 || protSpa4376
Complex III, QCR6	Sca	Scas_565.10
Complex III, QCR6	Sce	YFR034C || YFR033C
Complex III, QCR6	Sba	Sbay_29.17
Complex III, QCR7	Clu	CLUG_04964.1
Complex III, QCR7	Uma	UM04237.1
Complex III, QCR7	Pca	protPca1497 || protPca1499
Complex III, QCR7	Ncr	(NCU08940.2)
Complex III, QCR7	Sku	protSku4257
Complex III, QCR7	Sno	SNU05323.1
Complex III, QCR7	Cgu	PGUG_03438.1
Complex III, QCR7	Ror	RO3G_03897.1 || RO3G_09716.1
Complex III, QCR7	Afl	AFL2G_04721
Complex III, QCR7	Cci	CC1G_03856.1
Complex III, QCR7	Mgr	MGG_06192.5
Complex III, QCR7	Pan	protPan10758
Complex III, QCR7	Cdu	protCdu4192
Complex III, QCR7	Kla	XP_452231.1
Complex III, QCR7	Ctr	CTRG_02121.3
Complex III, QCR7	Sja	SJAG_03490
Complex III, QCR7	Spb	O74533
Complex III, QCR7	Gze	Q4HXL5
Complex III, QCR7	Pgr	PGTG_01340
Complex III, QCR7	Smi	protSmi4252
Complex III, QCR7	Nfi	NFIA_109440
Complex III, QCR7	Cal	orf19.5629
Complex III, QCR7	Fve	FVEG_03024
Complex III, QCR7	Cne	Q55UB7
Complex III, QCR7	Acl	ACLA_046080
Complex III, QCR7	Ath	Q9SUU5
Complex III, QCR7	Kwa	Kwal_33.13064
Complex III, QCR7	Ssc	SS1G_10576.1
Complex III, QCR7	Ppl	Pospl1125759
Complex III, QCR7	Yli	Q6C3K7
Complex III, QCR7	Nha	Necha273713
Complex III, QCR7	Ate	ATEG_05551.1
Complex III, QCR7	Hsa	ENSP00000287022 || ENSP00000335476
Complex III, QCR7	Sce	YDR529C
Complex III, QCR7	Spa	protSpa3335
Complex III, QCR7	Afu	Q4WNS5
Complex III, QCR7	Ago	NP_986279.1
Complex III, QCR7	Pch	Phchr1133055
Complex III, QCR7	Bci	BC1G_10635.1
Complex III, QCR7	Kpo	Kpol_2000.108
Complex III, QCR7	Cgo	CHG06996.1
Complex III, QCR7	Sro	Sporo122649
Complex III, QCR7	Pst	Picst373333
Complex III, QCR7	Pbl	Phybl140848
Complex III, QCR7	Sba	Sbay_21.8
Complex III, QCR7	Ang	A2QHV7

Complex III, QCR7	Skl	protSkl4721
Complex III, QCR7	Lbi	Lacbi1293408
Complex III, QCR7	Hca	HCAG_05626.1
Complex III, QCR7	Fox	FOXG_05112
Complex III, QCR7	Cim	CIMG_04106
Complex III, QCR7	Dha	Q6BNQ5
Complex III, QCR7	Tre	Trire223431
Complex III, QCR7	Lel	LELG_04924
Complex III, QCR7	Ure	UREG_05547.1
Complex III, QCR7	Sca	Scas_685.31
Complex III, QCR7	Cgl	CAGL0G10153g
Complex III, QCR7	Ani	XP_661992.1
Complex III, QCR8	Sca	Scas_651.20
Complex III, QCR8	Acl	ACLA_021950
Complex III, QCR8	Fve	FVEG_03036
Complex III, QCR8	Yli	Q6C387
Complex III, QCR8	Sja	SJAG_00080
Complex III, QCR8	Fox	FOXG_05099
Complex III, QCR8	Cne	Q55VQ6
Complex III, QCR8	Tre	Trire2124148
Complex III, QCR8	Lbi	Lacbi1293619
Complex III, QCR8	Smi	protSmi5155
Complex III, QCR8	Ate	ATEG_00550.1 || ATEG_03455.1
Complex III, QCR8	Bci	BC1G_15917.1
Complex III, QCR8	Mfi	Mycfi155290
Complex III, QCR8	Spb	P50523
Complex III, QCR8	Pan	protPan10643
Complex III, QCR8	Ssc	SS1G_10520.1
Complex III, QCR8	Kpo	Kpol_2002.72
Complex III, QCR8	Gze	Q4HXK3
Complex III, QCR8	Ppl	Pospl1119459 || Pospl1128149
Complex III, QCR8	Cdu	protCdu1586
Complex III, QCR8	Uma	UM03913.1
Complex III, QCR8	Cal	orf19.4490.2
Complex III, QCR8	Hca	HCAG_05012.1
Complex III, QCR8	Nha	Necha273715
Complex III, QCR8	Sku	protSku2375
Complex III, QCR8	Pbl	Phybl134478
Complex III, QCR8	Kwa	Kwal_33.13823
Complex III, QCR8	Dha	Q6BM52
Complex III, QCR8	Cim	CIMG_05742
Complex III, QCR8	Mgr	MGG_06003.5
Complex III, QCR8	Ncr	(NCU08947.2)
Complex III, QCR8	Lel	LELG_01960
Complex III, QCR8	Pgr	PGTG_05581
Complex III, QCR8	Afl	AFL2G_03428 || AFL2G_04149
Complex III, QCR8	Pst	Picst377955
Complex III, QCR8	Pch	Phchr13771
Complex III, QCR8	Ctr	CTRG_01607.3
Complex III, QCR8	Sba	Sbay_48.28
Complex III, QCR8	Cgl	CAGL0I06270g
Complex III, QCR8	Sno	SNU14805.1
Complex III, QCR8	Cci	CC1G_10306.1
Complex III, QCR8	Kla	XP_451301.1
Complex III, QCR8	Nfi	NFIA_012020
Complex III, QCR8	Afu	Q4WSA2
Complex III, QCR8	Ror	RO3G_02864.1 || RO3G_11468.1
Complex III, QCR8	Ang	A2QRM4
Complex III, QCR8	Sce	YJL166W
Complex III, QCR8	Ago	NP_984740.1
Complex III, QCR8	Cgu	PGUG_01466.1
Complex III, QCR9	Sca	Scas_688.33
Complex III, QCR9	Aor	Q2UN56
Complex III, QCR9	Ani	XP_661526.1
Complex III, QCR9	Bde	BDEG_06631
Complex III, QCR9	Ago	NP_982397.1
Complex III, QCR9	Kpo	Kpol_2002.92
Complex III, QCR9	Cgl	CAGL0H09328g
Complex III, QCR9	Ure	UREG_01051.1
Complex III, QCR9	Ssc	SS1G_13290.1
Complex III, QCR9	Hca	HCAG_05921.1
Complex III, QCR9	Yli	Q6CG23
Complex III, QCR9	Cgo	CHG01888.1
Complex III, QCR9	Ncr	(NCU03233.2)
Complex III, QCR9	Bci	BC1G_06710.1
Complex III, QCR9	Mgr	MGG_10604.5
Complex III, QCR9	Pbl	Phybl135772
Complex III, QCR9	Lbi	Lacbi1305710
Complex III, QCR9	Sce	YGR183C

Complex III, QCR9	Cne	Q55IQ4
Complex III, QCR9	Cim	CIMG_01108
Complex III, QCR9	Nfi	NFIA_054070
Complex III, QCR9	Acl	ACLA_082250
Complex III, QCR9	Cal	orf19.2707.1
Complex III, QCR9	Afl	AFL2G_09144
Complex III, QCR9	Ctr	CTRG_00262.3
Complex III, QCR9	Ror	RO3G_06079.1 || RO3G_15029.1
Complex III, QCR9	Kla	XP_455767.1
Complex III, QCR9	Cci	CC1G_00077.1
Complex III, QCR9	Uma	UM06122.1
Complex IV, Cox1	Hsa	ENSP00000354499
Complex IV, Cox1	Pca	protPca3573 || protPca3574
Complex IV, Cox1	Kla	YP_054500.1
Complex IV, Cox1	Ncr	Neur.crassa.mt8 || Neur.crassa.mt6
Complex IV, Cox1	Ath	P60620
Complex IV, Cox1	Sja	SJAG_mit2
Complex IV, Cox1	Pan	protPan12894
Complex IV, Cox1	Yli	Q9B6E6 || Q9B6E5 || Q9B6E7
Complex IV, Cox1	Ago	NP_987079.1
Complex IV, Cox1	Cne	None
Complex IV, Cox1	Uma	UM_mit10
Complex IV, Cox1	Spb	P07657
Complex IV, Cox1	Sce	Q0065 || Q0070 || Q0045
Complex IV, Cox10	Lel	LELG_03973
Complex IV, Cox10	Tre	Trire228159
Complex IV, Cox10	Sro	Sporo14233
Complex IV, Cox10	Ctr	CTRG_05324.3
Complex IV, Cox10	Skl	protSkl3506
Complex IV, Cox10	Pan	protPan500
Complex IV, Cox10	Ago	NP_982427.1
Complex IV, Cox10	Aor	Q2UGS9
Complex IV, Cox10	Sno	SNU00816.1
Complex IV, Cox10	Smi	protSmi1469
Complex IV, Cox10	Fve	FVEG_12748
Complex IV, Cox10	Cgu	PGUG_02600.1
Complex IV, Cox10	Ssc	SS1G_04105.1
Complex IV, Cox10	Sca	Scas_653.23
Complex IV, Cox10	Kwa	Kwal_26.8920
Complex IV, Cox10	Ani	XP_659326.1
Complex IV, Cox10	Sku	protSku1917
Complex IV, Cox10	Nfi	NFIA_107820
Complex IV, Cox10	Yli	Q6C0L2
Complex IV, Cox10	Bci	BC1G_00294.1
Complex IV, Cox10	Sba	Sbay_92.10
Complex IV, Cox10	Spb	Q9Y7Y4
Complex IV, Cox10	Gze	Q4I5G1
Complex IV, Cox10	Kpo	Kpol_1064.24
Complex IV, Cox10	Ath	O64886
Complex IV, Cox10	Cdu	protCdu3391
Complex IV, Cox10	Acl	ACLA_047710
Complex IV, Cox10	Dha	Q6BKW6
Complex IV, Cox10	Lbi	Lacbi1246255 || Lacbi1144184
Complex IV, Cox10	Pst	Picst347531
Complex IV, Cox10	Cci	CC1G_12378.1
Complex IV, Cox10	Uma	UM05692.1
Complex IV, Cox10	Nha	Necha271542
Complex IV, Cox10	Ncr	(NCU06141.2)
Complex IV, Cox10	Ror	RO3G_06322.1 || RO3G_14182.1 || RO3G_08531.1
Complex IV, Cox10	Fox	FOXG_15419
Complex IV, Cox10	Pgr	PGTG_18956 || PGTG_20110
Complex IV, Cox10	Ppl	Pospl1129368 || Pospl1129369 || Pospl188872 || Pospl135007
Complex IV, Cox10	Sce	YPL172C
Complex IV, Cox10	Afu	Q4WP81
Complex IV, Cox10	Clu	CLUG_02873.1
Complex IV, Cox10	Kla	XP_452623.1
Complex IV, Cox10	Pch	Phchr140726 || Phchr1136372 || Phchr1136635
Complex IV, Cox10	Cgo	CHG09848.1
Complex IV, Cox10	Cne	Q55M01
Complex IV, Cox10	Pca	protPca2454 || protPca2458
Complex IV, Cox10	Cal	orf19.3167
Complex IV, Cox10	Ate	ATEG_05362.1
Complex IV, Cox10	Pbl	Phybl123488
Complex IV, Cox10	Hsa	ENSP00000310873 || ENSP00000261643
Complex IV, Cox10	Afl	AFL2G_04540
Complex IV, Cox10	Cgl	CAGL0F03685g
Complex IV, Cox10	Ang	A2QID3
Complex IV, Cox10	Bde	BDEG_06331
Complex IV, Cox10	Spa	protSpa3832

Complex IV, Cox10	Mfi	Mycfi113893
Complex IV, Cox10	Mgr	MGG_05944.5
Complex IV, Cox10	Ure	UREG_02775.1
Complex IV, Cox10	Sja	SJAG_03173
Complex IV, Cox11	Lbi	Lacbi1297009
Complex IV, Cox11	Pch	Phchr132205
Complex IV, Cox11	Bci	BC1G_15559.1
Complex IV, Cox11	Nfi	NFIA_087430
Complex IV, Cox11	Cim	CIMG_05229
Complex IV, Cox11	Sce	YPL132W
Complex IV, Cox11	Bde	BDEG_00527
Complex IV, Cox11	Dha	Q6BGU6
Complex IV, Cox11	Pst	Picst382827
Complex IV, Cox11	Ang	A2QCP9
Complex IV, Cox11	Fox	FOXG_12779
Complex IV, Cox11	Uma	UM00726.1
Complex IV, Cox11	Ure	UREG_06395.1
Complex IV, Cox11	Smi	protSmi1959
Complex IV, Cox11	Cne	Q55YC6
Complex IV, Cox11	Fve	FVEG_11520
Complex IV, Cox11	Sno	SNU07655.1
Complex IV, Cox11	Afu	Q4X0U5
Complex IV, Cox11	Tre	Trire280932
Complex IV, Cox11	Ncr	(NCU01657.2)
Complex IV, Cox11	Sba	Sbay_93.128
Complex IV, Cox11	Pan	protPan4711
Complex IV, Cox11	Ssc	SS1G_14353.1
Complex IV, Cox11	Pca	protPca3829
Complex IV, Cox11	Gze	Q4HW12
Complex IV, Cox11	Cgo	CHG10327.1
Complex IV, Cox11	Afl	AFL2G_06848
Complex IV, Cox11	Cci	CC1G_06294.1
Complex IV, Cox11	Lel	LELG_04397
Complex IV, Cox11	Kla	XP_453373.1
Complex IV, Cox11	Mgr	MGG_11431.5
Complex IV, Cox11	Ago	NP_983508.1
Complex IV, Cox11	Aor	Q2UEZ1
Complex IV, Cox11	Spa	protSpa3868
Complex IV, Cox11	Spb	Q9UTM2 || Q9C0Y0
Complex IV, Cox11	Yli	Q6C6F0
Complex IV, Cox11	Pbl	Phybl116836
Complex IV, Cox11	Cgl	CAGL0M02673g
Complex IV, Cox11	Sku	protSku4448
Complex IV, Cox11	Cdu	protCdu607
Complex IV, Cox11	Hsa	ENSP00000299335
Complex IV, Cox11	Skl	protSkl4536
Complex IV, Cox11	Sca	Scas_700.41
Complex IV, Cox11	Ath	Q9FZ20 || Q8GWR0
Complex IV, Cox11	Ror	RO3G_10029.1
Complex IV, Cox11	Mfi	Mycfi128394
Complex IV, Cox11	Sja	SJAG_03322
Complex IV, Cox11	Kwa	Kwal_26.8660
Complex IV, Cox11	Cal	orf19.1416
Complex IV, Cox11	Clu	CLUG_03159.1
Complex IV, Cox11	Kpo	Kpol_1059.27
Complex IV, Cox11	Nha	Necha239830
Complex IV, Cox11	Ppl	Pospl187655
Complex IV, Cox11	Cgu	PGUG_04070.1
Complex IV, Cox11	Pgr	PGTG_15452
Complex IV, Cox11	Ctr	CTRG_00269.3
Complex IV, Cox11	Sro	Sporo113086
Complex IV, Cox11	Acl	ACLA_071130
Complex IV, Cox11	Ate	ATEG_01162.1
Complex IV, Cox15	Hsa	ENSP00000016171
Complex IV, Cox15	Aor	Q2UM16
Complex IV, Cox15	Kpo	Kpol_526.22
Complex IV, Cox15	Ago	NP_985559.1
Complex IV, Cox15	Sku	protSku6025
Complex IV, Cox15	Cgu	PGUG_03805.1
Complex IV, Cox15	Cal	orf19.3656
Complex IV, Cox15	Cgl	CAGL0I06831g
Complex IV, Cox15	Clu	CLUG_03692.1
Complex IV, Cox15	Kla	XP_454418.1
Complex IV, Cox15	Ssc	SS1G_02124.1
Complex IV, Cox15	Pch	Phchr138951
Complex IV, Cox15	Cim	CIMG_01309
Complex IV, Cox15	Uma	UM06392.1
Complex IV, Cox15	Ppl	Pospl1111299
Complex IV, Cox15	Bci	BC1G_06258.1

Complex IV, Cox15	Lel	LELG_04708
Complex IV, Cox15	Hca	HCAG_05715.1
Complex IV, Cox15	Sno	SNU12377.1
Complex IV, Cox15	Gze	Q4I1Z8
Complex IV, Cox15	Pgr	PGTG_07491 || PGTG_12543
Complex IV, Cox15	Mfi	Mycfi125551
Complex IV, Cox15	Cdu	protCdu4475
Complex IV, Cox15	Ate	ATEG_05900.1
Complex IV, Cox15	Tre	Trire24668
Complex IV, Cox15	Pst	Picst384097
Complex IV, Cox15	Ncr	(NCU04817.2)
Complex IV, Cox15	Yli	Q6C0H5
Complex IV, Cox15	Smi	protSmi5202
Complex IV, Cox15	Cne	Q55IF8
Complex IV, Cox15	Mgr	MGG_04969.5
Complex IV, Cox15	Sro	Sporo13356
Complex IV, Cox15	Pbl	Phybl111849
Complex IV, Cox15	Ang	A2QVS0
Complex IV, Cox15	Ctr	CTRG_02934.3
Complex IV, Cox15	Nfi	NFIA_053340
Complex IV, Cox15	Sce	YER141W
Complex IV, Cox15	Spa	protSpa2999
Complex IV, Cox15	Pan	protPan11195
Complex IV, Cox15	Kwa	Kwal_14.2027
Complex IV, Cox15	Ath	Q9FKT8
Complex IV, Cox15	Spb	Q10361
Complex IV, Cox15	Sca	Scas_710.16
Complex IV, Cox15	Nha	Necha2103747
Complex IV, Cox15	Sja	SJAG_04397
Complex IV, Cox15	Fve	FVEG_01873
Complex IV, Cox15	Afl	AFL2G_02787
Complex IV, Cox15	Fox	FOXG_03017
Complex IV, Cox15	Bde	BDEG_08667
Complex IV, Cox15	Ror	RO3G_01030.1
Complex IV, Cox15	Cgo	CHG08919.1
Complex IV, Cox15	Ure	UREG_01235.1
Complex IV, Cox15	Afu	Q4WN44
Complex IV, Cox15	Ani	XP_659519.1
Complex IV, Cox15	Acl	ACLA_081820
Complex IV, Cox15	Cci	CC1G_00134.1
Complex IV, Cox15	Skl	protSkl2295
Complex IV, Cox15	Dha	Q6BL98
Complex IV, Cox15	Lbi	Lacbi1144021
Complex IV, Cox15	Sba	Sbay_24.22
Complex IV, Cox17	Smi	protSmi234
Complex IV, Cox17	Yli	Q6CBM0
Complex IV, Cox17	Cgl	CAGL0D05632g
Complex IV, Cox17	Ago	NP_982381.1
Complex IV, Cox17	Sce	YLL009C
Complex IV, Cox17	Kla	XP_455785.1
Complex IV, Cox17	Clu	CLUG_05856.1
Complex IV, Cox17	Kpo	Kpol_2002.103
Complex IV, Cox17	Pst	Picst370893
Complex IV, Cox17	Dha	Q6BZH7
Complex IV, Cox17	Kwa	Kwal_33.15148
Complex IV, Cox2	Hsa	ENSP00000354876
Complex IV, Cox2	Cdu	protCdu3763
Complex IV, Cox2	Kla	YP_054502.1
Complex IV, Cox2	Ago	NP_987078.1
Complex IV, Cox2	Yli	Q9B6D5
Complex IV, Cox2	Pan	protPan12920
Complex IV, Cox2	Cal	CaalfMp01
Complex IV, Cox2	Sja	SJAG_mit9
Complex IV, Cox2	Ath	P92559 || P93285
Complex IV, Cox2	Sno	SNU16563.1
Complex IV, Cox2	Ncr	Neur.crassa.mt11 || Neur.crassa.mt10
Complex IV, Cox2	Sce	Q0250
Complex IV, Cox2	Sro	Sporo18231
Complex IV, Cox2	Spb	P21534
Complex IV, Cox2	Pbl	Phybl127168
Complex IV, Cox2	Cne	None
Complex IV, Cox2	Uma	UM_mit7
Complex IV, Cox3	Ath	Q8S8B5 || P92514
Complex IV, Cox3	Pan	protPan12885
Complex IV, Cox3	Hsa	ENSP00000354982
Complex IV, Cox3	Sno	SNU16567.1
Complex IV, Cox3	Sce	Q0275
Complex IV, Cox3	Kla	YP_054504.1
Complex IV, Cox3	Uma	UM_mit20

Complex IV, Cox3	Cal	CaalfMp04 || CaalfMp15
Complex IV, Cox3	Ncr	Neur.crassa.mt12
Complex IV, Cox3	Pbl	Phybl127133
Complex IV, Cox3	Ago	NP_987080.1
Complex IV, Cox3	Spb	P14575
Complex IV, Cox3	Sja	SJAG_mit7
Complex IV, Cox3	Yli	Q9B6D8
Complex IV, Cox4	Cdu	protCdu1885
Complex IV, Cox4	Mfi	Mycfi189362
Complex IV, Cox4	Cgu	PGUG_03701.1
Complex IV, Cox4	Gze	Q4IJ96
Complex IV, Cox4	Nha	Necha270219
Complex IV, Cox4	Ang	A2QNX9
Complex IV, Cox4	Ure	UREG_01785.1
Complex IV, Cox4	Sja	SJAG_00976
Complex IV, Cox4	Hsa	ENSP00000258424
Complex IV, Cox4	Smi	protSmi4701
Complex IV, Cox4	Ssc	SS1G_03202.1
Complex IV, Cox4	Pgr	PGTG_14276
Complex IV, Cox4	Cim	CIMG_04576
Complex IV, Cox4	Nfi	NFIA_035360
Complex IV, Cox4	Tre	Trire258493
Complex IV, Cox4	Dha	Q6BXA4
Complex IV, Cox4	Sca	Scas_710.31
Complex IV, Cox4	Lbi	Lacbi1173909
Complex IV, Cox4	Mgr	MGG_01135.5
Complex IV, Cox4	Sba	Sbay_32.130
Complex IV, Cox4	Sro	Sporo17159
Complex IV, Cox4	Spa	protSpa2219
Complex IV, Cox4	Yli	Q6C5A3
Complex IV, Cox4	Ppl	Pospl1117401
Complex IV, Cox4	Afl	AFL2G_06652
Complex IV, Cox4	Spb	P79010
Complex IV, Cox4	Pst	Picst388762
Complex IV, Cox4	Afu	Q4WI57
Complex IV, Cox4	Ctr	CTRG_01253.3
Complex IV, Cox4	Ath	Q9LW15 || Q9SSB8
Complex IV, Cox4	Aor	Q2UEE5
Complex IV, Cox4	Fve	FVEG_08989
Complex IV, Cox4	Cal	orf19.1471
Complex IV, Cox4	Pbl	Phybl122205
Complex IV, Cox4	Bci	BC1G_06743.1
Complex IV, Cox4	Cgo	CHG05362.1
Complex IV, Cox4	Cne	Q55M18
Complex IV, Cox4	Ani	XP_662129.1
Complex IV, Cox4	Pan	protPan2011
Complex IV, Cox4	Pch	Phchr143711
Complex IV, Cox4	Sce	YGL187C
Complex IV, Cox4	Uma	UM04802.1
Complex IV, Cox4	Kwa	Kwal_47.17214
Complex IV, Cox4	Fox	FOXG_10335
Complex IV, Cox4	Hca	HCAG_04564.1
Complex IV, Cox4	Ror	RO3G_04949.1 || RO3G_11037.1
Complex IV, Cox4	Cgl	CAGL0L06160g
Complex IV, Cox4	Cci	CC1G_12418.1
Complex IV, Cox4	Sno	SNU09790.1
Complex IV, Cox4	Bde	BDEG_02098
Complex IV, Cox4	Sku	protSku2523
Complex IV, Cox4	Ago	NP_985740.1
Complex IV, Cox4	Ncr	(NCU05689.2)
Complex IV, Cox4	Acl	ACLA_091830
Complex IV, Cox4	Kla	XP_453286.1
Complex IV, Cox4	Lel	LELG_00922
Complex IV, Cox4	Ate	ATEG_09593.1
Complex IV, Cox5	Dha	Q6BT71
Complex IV, Cox5	Lbi	Lacbi1163585
Complex IV, Cox5	Pch	Phchr1132624
Complex IV, Cox5	Smi	protSmi2915 || protSmi2815
Complex IV, Cox5	Afu	Q4WV26
Complex IV, Cox5	Ctr	CTRG_03244.3
Complex IV, Cox5	Pbl	Phybl116001 || Phybl134311
Complex IV, Cox5	Cci	CC1G_08304.1
Complex IV, Cox5	Yli	Q6C309
Complex IV, Cox5	Mfi	Mycfi159363
Complex IV, Cox5	Ago	NP_983853.1
Complex IV, Cox5	Cim	CIMG_06276
Complex IV, Cox5	Bci	BC1G_11967.1
Complex IV, Cox5	Sja	SJAG_01077
Complex IV, Cox5	Uma	UM05465.1

Complex IV, Cox5	Kla	XP_451055.1 || XP_455245.1
Complex IV, Cox5	Pan	protPan2937
Complex IV, Cox5	Fve	FVEG_11349
Complex IV, Cox5	Sba	Sbay_44.124 || Sbay_73.46
Complex IV, Cox5	Tre	Trire280288
Complex IV, Cox5	Mgr	MGG_03188.5
Complex IV, Cox5	Cal	orf19.4759
Complex IV, Cox5	Sro	Sporo16861
Complex IV, Cox5	Cgl	CAGL0I04136g || CAGL0C01325g
Complex IV, Cox5	Ure	UREG_04111.1
Complex IV, Cox5	Spa	protSpa4426 || protSpa1812
Complex IV, Cox5	Lel	LELG_00320
Complex IV, Cox5	Sca	Scas_671.29
Complex IV, Cox5	Fox	FOXG_13925
Complex IV, Cox5	Cgo	CHG10265.1
Complex IV, Cox5	Spb	O74988
Complex IV, Cox5	Cne	Q55KG2
Complex IV, Cox5	Skl	protSkl1740 || protSkl1774
Complex IV, Cox5	Afl	AFL2G_11644
Complex IV, Cox5	Ssc	SS1G_07796.1
Complex IV, Cox5	Bde	BDEG_07683
Complex IV, Cox5	Sno	SNU01277.1
Complex IV, Cox5	Cdu	protCdu5743
Complex IV, Cox5	Aor	Q2TWP1
Complex IV, Cox5	Sce	YIL111W || YNL052W
Complex IV, Cox5	Sku	protSku5701 || protSku1448
Complex IV, Cox5	Nfi	NFIA_076650
Complex IV, Cox5	Nha	Necha247779
Complex IV, Cox5	Ppl	Pospl1114514 || Pospl1125855
Complex IV, Cox5	Ncr	(NCU05457.2)
Complex IV, Cox5	Cgu	PGUG_05111.1
Complex IV, Cox5	Clu	CLUG_03838.1
Complex IV, Cox5	Kpo	Kpol_1039.27 || Kpol_460.2
Complex IV, Cox5	Ror	RO3G_11057.1 || RO3G_05140.1
Complex IV, Cox5	Acl	ACLA_013570
Complex IV, Cox5	Gze	Q4IEP3
Complex IV, Cox5	Pgr	PGTG_06603
Complex IV, Cox5	Ani	XP_659920.1
Complex IV, Cox5	Pst	Picst377110
Complex IV, Cox6	Ror	RO3G_10189.1 || RO3G_15433.1
Complex IV, Cox6	Skl	protSkl453
Complex IV, Cox6	Pbl	Phybl140034 || Phybl18549
Complex IV, Cox6	Ppl	Pospl1121167 || Pospl1110120
Complex IV, Cox6	Nha	Necha2101622
Complex IV, Cox6	Kwa	Kwal_23.5604
Complex IV, Cox6	Sro	Sporo114974
Complex IV, Cox6	Hca	HCAG_00437.1
Complex IV, Cox6	Afu	Q4WEE8
Complex IV, Cox6	Ctr	CTRG_01195.3
Complex IV, Cox6	Cim	CIMG_06473
Complex IV, Cox6	Cne	Q55XZ4
Complex IV, Cox6	Fve	FVEG_07914
Complex IV, Cox6	Yli	Q6C6E6
Complex IV, Cox6	Kla	XP_456057.1
Complex IV, Cox6	Ani	XP_681387.1
Complex IV, Cox6	Nfi	NFIA_039350
Complex IV, Cox6	Acl	ACLA_002410
Complex IV, Cox6	Cgo	CHG07758.1
Complex IV, Cox6	Kpo	Kpol_1051.18
Complex IV, Cox6	Ate	ATEG_09242.1
Complex IV, Cox6	Cal	orf19.873.1
Complex IV, Cox6	Lel	LELG_01281
Complex IV, Cox6	Ncr	(NCU06695.2)
Complex IV, Cox6	Ago	NP_983294.1
Complex IV, Cox6	Ssc	SS1G_01396.1
Complex IV, Cox6	Sno	SNU10938.1
Complex IV, Cox6	Spa	protSpa3556
Complex IV, Cox6	Ang	A2QEA0
Complex IV, Cox6	Spb	Q9UTF6
Complex IV, Cox6	Lbi	Lacbi1230418 || Lacbi1163968
Complex IV, Cox6	Pan	protPan706
Complex IV, Cox6	Smi	protSmi2387
Complex IV, Cox6	Pgr	PGTG_19036
Complex IV, Cox6	Uma	UM02477.1
Complex IV, Cox6	Afl	AFL2G_09806
Complex IV, Cox6	Pst	Picst382299
Complex IV, Cox6	Cci	CC1G_10112.1
Complex IV, Cox6	Fox	FOXG_01556
Complex IV, Cox6	Tre	Trire253318

Complex IV, Cox6	Sce	YHR051W
Complex IV, Cox6	Ure	UREG_03945.1
Complex IV, Cox6	Sku	protSku709
Complex IV, Cox6	Cgl	CAGL0J00429g
Complex IV, Cox6	Mgr	MGG_07281.5
Complex IV, Cox6	Mfi	Mycfi170565
Complex IV, Cox6	Sca	Scas_688.8
Complex IV, Cox6	Hsa	ENSP00000317780
Complex IV, Cox6	Sja	SJAG_01355
Complex IV, Cox6	Dha	Q6BNM0
Complex IV, Cox6	Bde	BDEG_01161
Complex IV, Cox6	Pch	Phchr134038
Complex IV, Cox6a	Sce	YGL191W
Complex IV, Cox6a	Acl	ACLA_034140
Complex IV, Cox6a	Pbl	Phybl175519
Complex IV, Cox6a	Ror	RO3G_09053.1 || RO3G_14410.1
Complex IV, Cox6a	Clu	CLUG_03087.1
Complex IV, Cox6a	Cgo	CHG06582.1
Complex IV, Cox6a	Kpo	Kpol_246.3
Complex IV, Cox6a	Kla	XP_455493.1
Complex IV, Cox6a	Skl	protSkl799
Complex IV, Cox6a	Cne	Q55WG7
Complex IV, Cox6a	Ang	A5ABR9
Complex IV, Cox6a	Lbi	Lacbi1291424
Complex IV, Cox6a	Hsa	ENSP00000229379 || ENSP00000297147 || ENSP00000287490
Complex IV, Cox6a	Gze	Q4I1M0
Complex IV, Cox6a	Sba	Sbay_32.133
Complex IV, Cox6a	Ate	ATEG_08810.1
Complex IV, Cox6a	Ncr	(NCU01962.2)
Complex IV, Cox6a	Cgu	PGUG_03697.1
Complex IV, Cox6a	Spa	protSpa2210
Complex IV, Cox6a	Sca	Scas_710.34
Complex IV, Cox6a	Pst	Picst367314
Complex IV, Cox6a	Spb	O74471
Complex IV, Cox6a	Cci	CC1G_10278.1
Complex IV, Cox6a	Pgr	PGTG_00398
Complex IV, Cox6a	Cdu	protCdu1884
Complex IV, Cox6a	Ani	XP_662341.1
Complex IV, Cox6a	Cim	CIMG_00404
Complex IV, Cox6a	Afl	AFL2G_11187
Complex IV, Cox6a	Nha	Necha299757
Complex IV, Cox6a	Nfi	NFIA_071070
Complex IV, Cox6a	Tre	Trire2121275
Complex IV, Cox6a	Cal	orf19.1467
Complex IV, Cox6a	Smi	protSmi4710
Complex IV, Cox6a	Hca	HCAG_01172.1
Complex IV, Cox6a	Afu	Q4WWK4
Complex IV, Cox6a	Mfi	Mycfi149391
Complex IV, Cox6a	Sno	SNU00378.1
Complex IV, Cox6a	Kwa	Kwal_47.17203
Complex IV, Cox6a	Fox	FOXG_03613
Complex IV, Cox6a	Yli	Q6C325
Complex IV, Cox6a	Mgr	MGG_02983.5
Complex IV, Cox6a	Lel	LELG_00919
Complex IV, Cox6a	Fve	FVEG_02487
Complex IV, Cox6a	Cgl	CAGL0L06204g
Complex IV, Cox6a	Dha	Q6BXA1
Complex IV, Cox6a	Pch	Phchr16513
Complex IV, Cox6a	Ctr	CTRG_01250.3
Complex IV, Cox6a	Uma	UM01147.1
Complex IV, Cox6a	Bci	BC1G_15145.1
Complex IV, Cox6a	Ago	NP_985743.1
Complex IV, Cox6b	Tre	Trire271343
Complex IV, Cox6b	Sno	SNU07412.1
Complex IV, Cox6b	Acl	ACLA_071820
Complex IV, Cox6b	Pan	protPan8585
Complex IV, Cox6b	Mgr	MGG_01111.5
Complex IV, Cox6b	Sro	Sporo131125
Complex IV, Cox6b	Skl	protSkl5734
Complex IV, Cox6b	Kla	XP_455857.1
Complex IV, Cox6b	Hca	HCAG_03761.1
Complex IV, Cox6b	Cal	orf19.1082.1
Complex IV, Cox6b	Cgu	PGUG_04022.1
Complex IV, Cox6b	Mfi	Mycfi160691
Complex IV, Cox6b	Fve	FVEG_04937
Complex IV, Cox6b	Kwa	Kwal_47.17817
Complex IV, Cox6b	Ath	Q9SUD3 || Q945L0 || Q9LPJ2 || Q9S7L9
Complex IV, Cox6b	Cgo	CHG07919.1
Complex IV, Cox6b	Sce	YLR038C

Complex IV, Cox6b	Dha	Q6BQX0
Complex IV, Cox6b	Fox	FOXG_08017
Complex IV, Cox6b	Bci	BC1G_15347.1
Complex IV, Cox6b	Bde	BDEG_01082
Complex IV, Cox6b	Ang	A2QBZ3
Complex IV, Cox6b	Pch	Phchr1136744
Complex IV, Cox6b	Lbi	Lacbi1190553
Complex IV, Cox6b	Sca	Scas_623.16
Complex IV, Cox6b	Sja	SJAG_02504
Complex IV, Cox6b	Cgl	CAGL0A03542g
Complex IV, Cox6b	Ate	ATEG_01267.1
Complex IV, Cox6b	Nfi	NFIA_088180
Complex IV, Cox6b	Ago	NP_984336.1
Complex IV, Cox6b	Spb	O94581
Complex IV, Cox6b	Ure	UREG_06443.1
Complex IV, Cox6b	Kpo	Kpol_1046.4 || Kpol_1041.18
Complex IV, Cox6b	Afu	Q4X0M1
Complex IV, Cox6b	Ctr	CTRG_02762.3
Complex IV, Cox6b	Cci	CC1G_13336.1
Complex IV, Cox6b	Cne	Q55QK1
Complex IV, Cox6b	Lel	LELG_04963
Complex IV, Cox6b	Gze	Q4I940
Complex IV, Cox6b	Afl	AFL2G_06925
Complex IV, Cox6b	Ani	XP_663859.1
Complex IV, Cox6b	Ror	RO3G_00018.1 || RO3G_08272.1
Complex IV, Cox6b	Pbl	Phybl124717
Complex IV, Cox6b	Sba	Sbay_59.19
Complex IV, Cox6b	Hsa	ENSP00000310393 || ENSP00000246554
Complex IV, Cox6b	Uma	UM02643.1
Complex IV, Cox6b	Pst	Picst371543
Complex IV, Cox6b	Clu	CLUG_05213.1
Complex IV, Cox6b	Ssc	SS1G_10490.1
Complex IV, Cox6b	Cdu	protCdu4286
Complex IV, Cox6b	Yli	Q6C5M8
Complex IV, Cox6b	Pgr	PGTG_12722
Complex IV, Cox6b	Nha	Necha273563
Complex IV, Cox6b	Sku	protSku3738
Complex IV, Cox6b	Ppl	Pospl138552
Complex IV, Cox7a	Gze	Q4HYS9
Complex IV, Cox7a	Sno	SNU11671.1
Complex IV, Cox7a	Ago	NP_984923.1
Complex IV, Cox7a	Kla	XP_451680.1
Complex IV, Cox7a	Cal	orf19.5213.2
Complex IV, Cox7a	Pbl	Phybl123674 || Phybl19902
Complex IV, Cox7a	Nfi	NFIA_062820
Complex IV, Cox7a	Mfi	Mycfi135895
Complex IV, Cox7a	Ncr	(NCU05816.2)
Complex IV, Cox7a	Cdu	protCdu2080
Complex IV, Cox7a	Hca	HCAG_08885.1
Complex IV, Cox7a	Ctr	CTRG_01517.3
Complex IV, Cox7a	Ssc	SS1G_00902.1
Complex IV, Cox7a	Sja	SJAG_00873
Complex IV, Cox7a	Afu	Q4WYV9
Complex IV, Cox7a	Pgr	PGTG_02431
Complex IV, Cox7a	Acl	ACLA_041890
Complex IV, Cox7a	Clu	CLUG_02073.1
Complex IV, Cox7a	Cne	Q55PF8
Complex IV, Cox7a	Sca	Scas_513.5
Complex IV, Cox7a	Cim	CIMG_05645
Complex IV, Cox7a	Sce	YDL067C
Complex IV, Cox7a	Bci	BC1G_10051.1
Complex IV, Cox7a	Cgl	CAGL0D00748g
Complex IV, Cox7a	Lel	LELG_00980
Complex IV, Cox7a	Mgr	MGG_12467.5
Complex IV, Cox7a	Dha	Q6BPV1
Complex IV, Cox7a	Kpo	Kpol_1055.76
Complex IV, Cox7a	Kwa	Kwal_23.4201
Complex IV, Cox7a	Smi	protSmi1382
Complex IV, Cox7a	Cgo	CHG08412.1
Complex IV, Cox7a	Ang	A2QU13
Complex IV, Cox7a	Fox	FOXG_05547
Complex IV, Cox7a	Fve	FVEG_02593
Complex IV, Cox7a	Cgu	PGUG_03662.1
Complex IV, Cox7a	Afl	AFL2G_06203
Complex IV, Cox7a	Spb	O94705
Complex IV, Cox7a	Ror	RO3G_01074.1
Complex IV, Cox7a	Nha	Necha273669
Complex IV, Cox7a	Ure	UREG_06105.1
Complex IV, Cox8 / Cox7c	Cgl	CAGL0C02623g

Complex IV, Cox8 / Cox7c	Kpo	Kpol_1055.77
Complex IV, Cox8 / Cox7c	Ago	NP_984922.1
Complex IV, Cox8 / Cox7c	Cgu	PGUG_03661.1
Complex IV, Cox8 / Cox7c	Cal	orf19.5213.1
Complex IV, Cox8 / Cox7c	Dha	Q6BPV0
Complex IV, Cox8 / Cox7c	Cdu	protCdu1523
Complex IV, Cox8 / Cox7c	Kwa	Kwal_23.4203
Complex IV, Cox8 / Cox7c	Sba	Sbay_64.61
Complex IV, Cox8 / Cox7c	Sce	YLR395C
Complex IV, Cox8 / Cox7c	Sca	Scas_719.18
Complex IV, Cox8 / Cox7c	Yli	Q6C2Y3
Complex IV, Cox8 / Cox7c	Kla	XP_451681.1
Complex IV, Cox8 / Cox7c	Ctr	CTRG_01518.3
Complex V, F-type ATPase 8	Sce	Q0080
Complex V, F-type ATPase 8	Ago	NP_987082.1
Complex V, F-type ATPase 8	Kla	YP_054499.1
Complex V, F-type ATPase 8	Sno	SNU14769.1
Complex V, F-type ATPase A	Yli	Q36258
Complex V, F-type ATPase A	Ncr	Neur.crassa.mt3 || Neur.crassa.mt4
Complex V, F-type ATPase A	Hsa	ENSP00000354632
Complex V, F-type ATPase A	Cne	None
Complex V, F-type ATPase A	Spb	P21535
Complex V, F-type ATPase A	Sce	Q0085
Complex V, F-type ATPase A	Ath	P93298 || P92547
Complex V, F-type ATPase A	Sja	SJAG_mit8
Complex V, F-type ATPase A	Kla	YP_054498.1
Complex V, F-type ATPase A	Uma	UM_mit8
Complex V, F-type ATPase A	Ago	NP_987083.1
Complex V, F-type ATPase A	Nfi	NFIA_023760 || NFIA_042430
Complex V, F-type ATPase A	Pan	protPan12888
Complex V, F-type ATPase A	Cal	CaalfMp06
Complex V, F-type ATPase alpha	Cgu	PGUG_00565.1 || PGUG_01384.1 || PGUG_01133.1 || PGUG_01621.1
Complex V, F-type ATPase alpha	Ang	A5AAF1 || A2R8J9 || A2QC23
Complex V, F-type ATPase alpha	Sku	protSku4753 || protSku3659 || protSku3752 || protSku3376
Complex V, F-type ATPase alpha	Sca	Scas_77.1 || Scas_516.6 || Scas_445.2 || Scas_691.28
Complex V, F-type ATPase alpha	Clu	CLUG_01995.1 || CLUG_00278.1 || CLUG_01226.1 || CLUG_00735.1
Complex V, F-type ATPase alpha	Cgo	CHG01119.1 || CHG10169.1 || CHG10255.1
Complex V, F-type ATPase alpha	Dha	Q6BRW3 || Q6BNG9 || Q6BME0
Complex V, F-type ATPase alpha	Gze	Q4I704 || Q4INX9 || Q4IEP6 || Q4IQ71 || Q4INX8
Complex V, F-type ATPase alpha	Cim	CIMG_06447 || CIMG_04309 || CIMG_05077 || CIMG_06274
Complex V, F-type ATPase alpha	Cdu	protCdu5077 || protCdu6000 || protCdu2557 || protCdu416
Complex V, F-type ATPase alpha	Kpo	Kpol_538.15 || Kpol_1045.9 || Kpol_1018.115 || Kpol_1008.8 || Kpol_1013.57
Complex V, F-type ATPase alpha	Pst	Picst386570 || Picst348891 || Picst377492
Complex V, F-type ATPase alpha	Yli	Q6CHD8 || Q6C326 || Q6CFT7 || Q6C4E9
Complex V, F-type ATPase alpha	Cgl	CAGL0I03960g || CAGL0M09581g || CAGL0C00649g || CAGL0H00506g
Complex V, F-type ATPase alpha	Cne	Q55SG2 || Q55QU6 || Q55NH5 || Q55QC5
Complex V, F-type ATPase alpha	Ppl	Pospl1107265 || Pospl1124091 || Pospl1124052 || Pospl1111462 || Pospl143833 || Pospl1120163
Complex V, F-type ATPase alpha	Nha	Necha271913 || Necha230907 || Necha263013 || Necha298895
Complex V, F-type ATPase alpha	Smi	protSmi1528 || protSmi5413 || protSmi859 || protSmi4797
Complex V, F-type ATPase alpha	Sce	YBL099W || YBR127C || YJR121W
Complex V, F-type ATPase alpha	Ctr	CTRG_04698.3 || CTRG_00643.3 || CTRG_00125.3
Complex V, F-type ATPase alpha	Pbl	Phybl157524 || Phybl128680 || Phybl175344 || Phybl139702 || Phybl137056 || Phybl134978 || Phybl127345 || Phybl157829 || Phybl129846 || Phybl177568 || Phybl141013 || Phybl132013
Complex V, F-type ATPase alpha	Spa	protSpa4317 || protSpa1144 || protSpa4581 || protSpa2648
Complex V, F-type ATPase alpha	Lel	LELG_01813 || LELG_03166 || LELG_04299
Complex V, F-type ATPase alpha	Ecu	Q8SQU9 || Q8SR34
Complex V, F-type ATPase alpha	Afl	AFL2G_09767 || AFL2G_00612 || AFL2G_06969 || AFL2G_11645
Complex V, F-type ATPase alpha	Kwa	Kwal_56.24421 || Kwal_27.10636 || Kwal_55.21868 || Kwal_23.3884
Complex V, F-type ATPase alpha	Sba	Sbay_14.23 || Sbay_6.99 || Sbay_4.145 || Sbay_52.46
Complex V, F-type ATPase alpha	Ncr	(NCU01207.2) || (NCU08515.2) || (NCU02514.2) || Neur.crassa.mt5
Complex V, F-type ATPase alpha	Afu	Q4WEB0 || Q4WC88 || Q4X0J7 || Q4WV25
Complex V, F-type ATPase alpha	Ani	XP_681290.1 || XP_662688.1 || XP_659127.1 || XP_663836.1 || XP_659919.1
Complex V, F-type ATPase alpha	Ssc	SS1G_03787.1 || SS1G_10682.1 || SS1G_07793.1 || SS1G_07886.1
Complex V, F-type ATPase alpha	Sja	SJAG_01471 || SJAG_00136 || SJAG_02564 || SJAG_02598
Complex V, F-type ATPase alpha	Ror	RO3G_01898.1 || RO3G_03665.1 || RO3G_01285.1 || RO3G_09588.1 || RO3G_12684.1 || RO3G_08262.1 || RO3G_16396.1 || RO3G_05483.1 || RO3G_00738.1 || RO3G_17086.1
Complex V, F-type ATPase alpha	Fve	FVEG_08178 || FVEG_01210 || FVEG_00559 || FVEG_11352
Complex V, F-type ATPase alpha	Mfi	Mycfi157218 || Mycfi148116 || Mycfi171317 || Mycfi188739
Complex V, F-type ATPase alpha	Lbi	Lacbi1189496 || Lacbi1182825 || Lacbi1187973 || Lacbi1305432
Complex V, F-type ATPase alpha	Mgr	MGG_08087.5 || MGG_03244.5 || MGG_07752.5 || MGG_03185.5
Complex V, F-type ATPase alpha	Pch	Phchr1123500 || Phchr1123444 || Phchr1124432 || Phchr1137299
Complex V, F-type ATPase alpha	Sno	SNU11021.1 || SNU01298.1 || SNU07651.1 || SNU04211.1 || SNU04213.1
Complex V, F-type ATPase alpha	Cci	CC1G_05193.1 || CC1G_12318.1 || CC1G_01082.1 || CC1G_09000.1
Complex V, F-type ATPase alpha	Aor	Q2UAK5 || Q2US06 || Q2UFA6 || Q2TWP0
Complex V, F-type ATPase alpha	Pca	protPca3436 || protPca1016
Complex V, F-type ATPase alpha	Pan	protPan5315 || protPan12731 || protPan5100 || protPan2953 || protPan12726
Complex V, F-type ATPase alpha	Bde	BDEG_07758 || BDEG_05613 || BDEG_05283 || BDEG_02989
Complex V, F-type ATPase alpha	Hsa	ENSP00000273398 || ENSP00000262030 || ENSP00000234396 || ENSP00000276390 || ENSP00000282050 || ENSP00000322305

Complex V, F-type ATPase alpha	Cal	orf19.1680 || orf19.6854 || orf19.6634 || orf19.5653
Complex V, F-type ATPase alpha	Pgr	PGTG_15754 || PGTG_04166 || PGTG_04870 || PGTG_15605 || PGTG_14303
Complex V, F-type ATPase alpha	Hca	HCAG_00404.1 || HCAG_02813.1 || HCAG_06944.1 || HCAG_05951.1
Complex V, F-type ATPase alpha	Ure	UREG_03969.1 || UREG_01564.1 || UREG_06480.1 || UREG_04109.1
Complex V, F-type ATPase alpha	Fox	FOXG_00954 || FOXG_13928
Complex V, F-type ATPase alpha	Spb	P31406 || P31411 || P24487 || P22068
Complex V, F-type ATPase alpha	Kla	XP_454248.1 || XP_453470.1 || XP_453538.1
Complex V, F-type ATPase alpha	Ago	NP_984198.1 || NP_983716.1 || NP_986395.1 || NP_983117.1
Complex V, F-type ATPase alpha	Uma	UM00621.1 || UM05379.1 || UM01618.1 || UM03191.1
Complex V, F-type ATPase alpha	Tre	Trire2121367 || Trire276057 || Trire221673 || Trire2123071
Complex V, F-type ATPase alpha	Bci	BC1G_04232.1 || BC1G_07780.1
Complex V, F-type ATPase alpha	Acl	ACLA_002890 || ACLA_072040 || ACLA_013560 || ACLA_058750
Complex V, F-type ATPase alpha	Sro	Sporo118641 || Sporo12639 || Sporo122520 || Sporo115231
Complex V, F-type ATPase alpha	Ate	ATEG_09203.1 || ATEG_09616.1 || ATEG_01300.1 || ATEG_04767.1 || ATEG_07609.1
Complex V, F-type ATPase alpha	Nfi	NFIA_039710 || NFIA_098070 || NFIA_088410 || NFIA_076660
Complex V, F-type ATPase alpha	Ath	O23654 || P19366 || P56757 || Q9SZN1 || Q9C5A9 || P92549 || P11574 || P83483 || P83484 || Q3ED91 || Q9LN19
Complex V, F-type ATPase alpha	Skl	protSkl3820 || protSkl5227 || protSkl3689
Complex V, F-type ATPase B	Spb	O94373
Complex V, F-type ATPase B	Hca	HCAG_02828.1
Complex V, F-type ATPase B	Ago	NP_984610.1
Complex V, F-type ATPase B	Hsa	ENSP00000235094
Complex V, F-type ATPase B	Tre	Trire276166
Complex V, F-type ATPase B	Mgr	MGG_04752.5
Complex V, F-type ATPase B	Lbi	Lacbi1249479
Complex V, F-type ATPase B	Cne	Q55V56
Complex V, F-type ATPase B	Sro	Sporo128628
Complex V, F-type ATPase B	Aor	Q2US19
Complex V, F-type ATPase B	Fve	FVEG_00828
Complex V, F-type ATPase B	Ssc	SS1G_01407.1
Complex V, F-type ATPase B	Kpo	Kpol_1024.38
Complex V, F-type ATPase B	Skl	protSkl4830
Complex V, F-type ATPase B	Cdu	protCdu2062
Complex V, F-type ATPase B	Cgu	PGUG_01452.1
Complex V, F-type ATPase B	Afu	Q4WC76
Complex V, F-type ATPase B	Mfi	Mycfi185960
Complex V, F-type ATPase B	Kla	XP_455015.1
Complex V, F-type ATPase B	Kwa	Kwal_27.12075
Complex V, F-type ATPase B	Cal	orf19.3579
Complex V, F-type ATPase B	Pgr	PGTG_15044
Complex V, F-type ATPase B	Ate	ATEG_04777.1
Complex V, F-type ATPase B	Gze	Q4IQY5
Complex V, F-type ATPase B	Fox	FOXG_00683
Complex V, F-type ATPase B	Ang	A2R8I7
Complex V, F-type ATPase B	Cgl	CAGL0H05489g
Complex V, F-type ATPase B	Smi	protSmi2641
Complex V, F-type ATPase B	Sba	Sbay_93.75
Complex V, F-type ATPase B	Afl	AFL2G_00599
Complex V, F-type ATPase B	Ncr	(NCU00502.2)
Complex V, F-type ATPase B	Sca	Scas_664.12
Complex V, F-type ATPase B	Acl	ACLA_058860
Complex V, F-type ATPase B	Ppl	Pospl1108477 || Pospl1120453
Complex V, F-type ATPase B	Ctr	CTRG_01562.3
Complex V, F-type ATPase B	Cgo	CHG05945.1
Complex V, F-type ATPase B	Bde	BDEG_03102
Complex V, F-type ATPase B	Nha	Necha2102380
Complex V, F-type ATPase B	Cci	CC1G_09897.1
Complex V, F-type ATPase B	Sce	YPL078C
Complex V, F-type ATPase B	Ani	XP_659138.1
Complex V, F-type ATPase B	Nfi	NFIA_098180
Complex V, F-type ATPase B	Bci	BC1G_07931.1
Complex V, F-type ATPase B	Sku	protSku3515
Complex V, F-type ATPase B	Sja	SJAG_00836
Complex V, F-type ATPase B	Pst	Picst378422
Complex V, F-type ATPase B	Pan	protPan2616
Complex V, F-type ATPase B	Dha	Q6BM66
Complex V, F-type ATPase B	Spa	protSpa3914
Complex V, F-type ATPase B	Ure	UREG_01575.1
Complex V, F-type ATPase B	Sno	SNU04313.1
Complex V, F-type ATPase B	Ror	RO3G_17073.1 || RO3G_13208.1 || RO3G_12242.1
Complex V, F-type ATPase B	Pch	Phchr1137780
Complex V, F-type ATPase B	Pbl	Phybl178520 || Phybl135889
Complex V, F-type ATPase B	Cim	CIMG_04320
Complex V, F-type ATPase B	Yli	Q6C105
Complex V, F-type ATPase B	Uma	UM05277.1
Complex V, F-type ATPase beta	Pan	protPan5315 || protPan5100 || protPan12726 || protPan2953
Complex V, F-type ATPase beta	Ang	A5AAF1 || A2QC23 || A2R8J9
Complex V, F-type ATPase beta	Aor	Q2UAK5 || Q2UFA6 || Q2US06 || Q2TWP0
Complex V, F-type ATPase beta	Mgr	MGG_08087.5 || MGG_03244.5 || MGG_07752.5 || MGG_03185.5

Complex V, F-type ATPase beta	Afu	Q4WEB0 || Q4WC88 || Q4X0J7 || Q4WV25
Complex V, F-type ATPase beta	Spa	protSpa4317 || protSpa4581 || protSpa1144 || protSpa2648
Complex V, F-type ATPase beta	Ure	UREG_03969.1 || UREG_06480.1 || UREG_04109.1
Complex V, F-type ATPase beta	Spb	P31406 || P31411 || P24487 || P22068
Complex V, F-type ATPase beta	Lbi	Lacbi1189496 || Lacbi1187973 || Lacbi1182825 || Lacbi1305432
Complex V, F-type ATPase beta	Hca	HCAG_00404.1 || HCAG_05951.1 || HCAG_06944.1
Complex V, F-type ATPase beta	Clu	CLUG_01995.1 || CLUG_01226.1 || CLUG_00735.1 || CLUG_00278.1
Complex V, F-type ATPase beta	Sro	Sporo118641 || Sporo122520 || Sporo12639 || Sporo115231
Complex V, F-type ATPase beta	Pgr	PGTG_15754 || PGTG_04870 || PGTG_04166 || PGTG_15605
Complex V, F-type ATPase beta	Cne	Q55SG2 || Q55QU6 || Q55NH5 || Q55QC5
Complex V, F-type ATPase beta	Ncr	(NCU01207.2) || (NCU08515.2) || Neur.crassa.mt5 || (NCU02514.2) || (NCU05430.2)
Complex V, F-type ATPase beta	Pst	Picst375615 || Picst386570 || Picst348891 || Picst377492
Complex V, F-type ATPase beta	Ecu	Q8SQU9 || Q8SR34
Complex V, F-type ATPase beta	Sba	Sbay_14.23 || Sbay_6.99 || Sbay_4.145 || Sbay_52.46
Complex V, F-type ATPase beta	Ath	O23654 || P19366 || P83484 || P92549 || P56757 || P83483 || Q9C5A9 || P11574 || Q9SZN1 || Q9LN19 || Q3ED91
Complex V, F-type ATPase beta	Afl	AFL2G_09767 || AFL2G_06969 || AFL2G_00612 || AFL2G_11645
Complex V, F-type ATPase beta	Lel	LELG_02227 || LELG_03166 || LELG_01813 || LELG_04299
Complex V, F-type ATPase beta	Tre	Trire2121367 || Trire276057 || Trire221673 || Trire2123071
Complex V, F-type ATPase beta	Kla	XP_455325.1 || XP_454248.1 || XP_453538.1 || XP_453470.1
Complex V, F-type ATPase beta	Skl	protSkl3820 || protSkl5227 || protSkl3689
Complex V, F-type ATPase beta	Ssc	SS1G_03787.1 || SS1G_10682.1 || SS1G_07886.1 || SS1G_07793.1
Complex V, F-type ATPase beta	Ago	NP_984198.1 || NP_983716.1 || NP_986395.1 || NP_983117.1
Complex V, F-type ATPase beta	Sno	SNU11021.1 || SNU07651.1 || SNU01298.1
Complex V, F-type ATPase beta	Ppl	Pospl1107265 || Pospl1124091 || Pospl1124052 || Pospl1111462 || Pospl1120163 || Pospl143833
Complex V, F-type ATPase beta	Kpo	Kpol_1020.3 || Kpol_538.15 || Kpol_1045.9 || Kpol_1018.115 || Kpol_1008.8 || Kpol_1013.57
Complex V, F-type ATPase beta	Cdu	protCdu5077 || protCdu2557 || protCdu6000 || protCdu416
Complex V, F-type ATPase beta	Ctr	CTRG_02148.3 || CTRG_00643.3 || CTRG_04698.3 || CTRG_00125.3
Complex V, F-type ATPase beta	Ani	XP_681290.1 || XP_662688.1 || XP_659127.1 || XP_663836.1 || XP_659919.1
Complex V, F-type ATPase beta	Sca	Scas_624.1 || Scas_77.1 || Scas_445.2 || Scas_516.6 || Scas_691.28
Complex V, F-type ATPase beta	Gze	Q4I704 || Q4IQ71 || Q4INX8 || Q4IEP6
Complex V, F-type ATPase beta	Pca	protPca3335 || protPca3436 || protPca630
Complex V, F-type ATPase beta	Sce	YDL185W || YBL099W || YBR127C || YJR121W
Complex V, F-type ATPase beta	Ror	RO3G_01898.1 || RO3G_03665.1 || RO3G_12684.1 || RO3G_09588.1 || RO3G_16396.1 || RO3G_08262.1 || RO3G_10980.1 || RO3G_01285.1 || RO3G_00738.1 || RO3G_05483.1 || RO3G_17086.1
Complex V, F-type ATPase beta	Nha	Necha271913 || Necha298895 || Necha230907 || Necha263013
Complex V, F-type ATPase beta	Ate	ATEG_09203.1 || ATEG_09616.1 || ATEG_04767.1 || ATEG_01300.1 || ATEG_07609.1
Complex V, F-type ATPase beta	Cgu	PGUG_00565.1 || PGUG_01133.1 || PGUG_01384.1 || PGUG_01621.1
Complex V, F-type ATPase beta	Smi	protSmi1528 || protSmi859 || protSmi5413 || protSmi4797
Complex V, F-type ATPase beta	Dha	Q6BRM0 || Q6BRW3 || Q6BNG9 || Q6BME0
Complex V, F-type ATPase beta	Fve	FVEG_08178 || FVEG_01210 || FVEG_00559 || FVEG_11352
Complex V, F-type ATPase beta	Sja	SJAG_01471 || SJAG_00136 || SJAG_02564 || SJAG_02598
Complex V, F-type ATPase beta	Pch	Phchr1123500 || Phchr1123444 || Phchr1124432 || Phchr1137299
Complex V, F-type ATPase beta	Cci	CC1G_05193.1 || CC1G_01082.1 || CC1G_12318.1 || CC1G_09000.1
Complex V, F-type ATPase beta	Nfi	NFIA_039710 || NFIA_098070 || NFIA_088410 || NFIA_076660
Complex V, F-type ATPase beta	Uma	UM00621.1 || UM03191.1 || UM05379.1 || UM01618.1
Complex V, F-type ATPase beta	Bde	BDEG_07758 || BDEG_05613 || BDEG_05283 || BDEG_02989
Complex V, F-type ATPase beta	Cgl	CAGL0I03960g || CAGL0M09581g || CAGL0C00649g || CAGL0H00506g
Complex V, F-type ATPase beta	Pbl	Phybl157524 || Phybl128680 || Phybl137056 || Phybl139702 || Phybl175344 || Phybl127345 || Phybl134978 || Phybl129846 || Phybl177568 || Phybl157829 || Phybl141013 || Phybl132013
Complex V, F-type ATPase beta	Cgo	CHG07700.1 || CHG10169.1 || CHG10255.1
Complex V, F-type ATPase beta	Cim	CIMG_06447 || CIMG_04309 || CIMG_05077 || CIMG_06274
Complex V, F-type ATPase beta	Fox	FOXG_00954 || FOXG_13928
Complex V, F-type ATPase beta	Acl	ACLA_002890 || ACLA_058750 || ACLA_072040 || ACLA_013560
Complex V, F-type ATPase beta	Bci	BC1G_04232.1 || BC1G_07780.1 || BC1G_11962.1
Complex V, F-type ATPase beta	Yli	Q6CHD8 || Q6C326 || Q6C4E9 || Q6CFT7
Complex V, F-type ATPase beta	Kwa	Kwal_56.24421 || Kwal_27.10636 || Kwal_23.3884 || Kwal_55.21868
Complex V, F-type ATPase beta	Sku	protSku4753 || protSku3752 || protSku3659 || protSku3376
Complex V, F-type ATPase beta	Hsa	ENSP00000273398 || ENSP00000262030 || ENSP00000282050 || ENSP00000322305 || ENSP00000276390 || ENSP00000234396
Complex V, F-type ATPase beta	Cal	orf19.1680 || orf19.6634 || orf19.6854 || orf19.5653
Complex V, F-type ATPase beta	Mfi	Mycfi157218 || Mycfi148116 || Mycfi171317 || Mycfi188739
Complex V, F-type ATPase C (ATP9)	Sno	SNU13334.1
Complex V, F-type ATPase C (ATP9)	Kwa	Kwal_6.519
Complex V, F-type ATPase C (ATP9)	Cne	None
Complex V, F-type ATPase C (ATP9)	Uma	UM_mit25
Complex V, F-type ATPase C (ATP9)	Sja	SJAG_mit6
Complex V, F-type ATPase C (ATP9)	Cdu	protCdu3748
Complex V, F-type ATPase C (ATP9)	Hsa	ENSP00000340315 || ENSP00000284727 || ENSP00000290313
Complex V, F-type ATPase C (ATP9)	Nfi	NFIA_046330
Complex V, F-type ATPase C (ATP9)	Pan	protPan11327 || protPan2758
Complex V, F-type ATPase C (ATP9)	Yli	Q37695
Complex V, F-type ATPase C (ATP9)	Cal	CaalfMp05
Complex V, F-type ATPase C (ATP9)	Ncr	(NCU02250.2)
Complex V, F-type ATPase C (ATP9)	Ago	NP_987084.1
Complex V, F-type ATPase C (ATP9)	Sce	Q0130
Complex V, F-type ATPase C (ATP9)	Cgo	CHG02320.1
Complex V, F-type ATPase C (ATP9)	Mgr	MGG_13815.5
Complex V, F-type ATPase C (ATP9)	Gze	Q4I9R0

Complex V, F-type ATPase C (ATP9)	Spb	P21537
Complex V, F-type ATPase D	Sno	SNU07800.1
Complex V, F-type ATPase D	Cim	CIMG_10149
Complex V, F-type ATPase D	Afl	AFL2G_05815
Complex V, F-type ATPase D	Pst	Picst379036
Complex V, F-type ATPase D	Cgl	CAGL0L09713g
Complex V, F-type ATPase D	Ssc	SS1G_05285.1
Complex V, F-type ATPase D	Sja	SJAG_03925
Complex V, F-type ATPase D	Dha	Q6BK20
Complex V, F-type ATPase D	Cci	CC1G_00461.1
Complex V, F-type ATPase D	Cgo	CHG06274.1
Complex V, F-type ATPase D	Ppl	Pospl1126327 || Pospl1126669
Complex V, F-type ATPase D	Cal	orf19.2785
Complex V, F-type ATPase D	Nfi	NFIA_050330
Complex V, F-type ATPase D	Clu	CLUG_02592.1
Complex V, F-type ATPase D	Spb	O94390
Complex V, F-type ATPase D	Pgr	PGTG_20020
Complex V, F-type ATPase D	Tre	Trire280216
Complex V, F-type ATPase D	Sca	Scas_542.7
Complex V, F-type ATPase D	Sba	Sbay_53.195
Complex V, F-type ATPase D	Cne	Q55Y96
Complex V, F-type ATPase D	Ctr	CTRG_04170.3
Complex V, F-type ATPase D	Cgu	PGUG_03092.1
Complex V, F-type ATPase D	Nha	Necha267487
Complex V, F-type ATPase D	Ate	ATEG_07080.1
Complex V, F-type ATPase D	Kla	XP_454767.1
Complex V, F-type ATPase D	Mgr	MGG_02661.5
Complex V, F-type ATPase D	Ang	A2R4V2
Complex V, F-type ATPase D	Ani	XP_664235.1
Complex V, F-type ATPase D	Ago	NP_983307.1
Complex V, F-type ATPase D	Ure	UREG_03565.1
Complex V, F-type ATPase D	Sku	protSku2158
Complex V, F-type ATPase D	Uma	UM06449.1
Complex V, F-type ATPase D	Ror	RO3G_11396.1 || RO3G_01495.1
Complex V, F-type ATPase D	Bde	BDEG_08285
Complex V, F-type ATPase D	Sce	YKL016C
Complex V, F-type ATPase D	Lel	LELG_00827
Complex V, F-type ATPase D	Pbl	Phybl142055
Complex V, F-type ATPase D	Mfi	Mycfi155574
Complex V, F-type ATPase D	Hca	HCAG_05098.1
Complex V, F-type ATPase D	Cdu	protCdu6130
Complex V, F-type ATPase D	Pch	Phchr1139401
Complex V, F-type ATPase D	Pan	protPan8941
Complex V, F-type ATPase D	Lbi	Lacbi1312017
Complex V, F-type ATPase D	Sro	Sporo17010
Complex V, F-type ATPase D	Smi	protSmi285
Complex V, F-type ATPase D	Afu	Q4WD80
Complex V, F-type ATPase D	Acl	ACLA_096800
Complex V, F-type ATPase D	Aor	Q2U954
Complex V, F-type ATPase D	Kwa	Kwal_14.913
Complex V, F-type ATPase D	Fox	FOXG_03373
Complex V, F-type ATPase D	Bci	BC1G_09982.1
Complex V, F-type ATPase D	Spa	protSpa4063
Complex V, F-type ATPase D	Kpo	Kpol_1057.5
Complex V, F-type ATPase D	Yli	Q6CFH9
Complex V, F-type ATPase D	Fve	FVEG_02245
Complex V, F-type ATPase D	Skl	protSkl4319
Complex V, F-type ATPase D	Pca	protPca654
Complex V, F-type ATPase delta	Gze	Q4I810
Complex V, F-type ATPase delta	Ror	RO3G_08261.1 || RO3G_16397.1
Complex V, F-type ATPase delta	Ani	XP_657881.1
Complex V, F-type ATPase delta	Mfi	Mycfi154841
Complex V, F-type ATPase delta	Sca	Scas_460.1
Complex V, F-type ATPase delta	Afl	AFL2G_00755
Complex V, F-type ATPase delta	Nha	Necha260460
Complex V, F-type ATPase delta	Sce	YDL004W
Complex V, F-type ATPase delta	Fox	FOXG_02293
Complex V, F-type ATPase delta	Sku	protSku1936
Complex V, F-type ATPase delta	Fve	FVEG_05469
Complex V, F-type ATPase delta	Ang	A2Q8N0
Complex V, F-type ATPase delta	Dha	Q6BXY0
Complex V, F-type ATPase delta	Kla	XP_455744.1
Complex V, F-type ATPase delta	Yli	Q6C877
Complex V, F-type ATPase delta	Pgr	PGTG_09048
Complex V, F-type ATPase delta	Mgr	MGG_05742.5
Complex V, F-type ATPase delta	Nfi	NFIA_021490
Complex V, F-type ATPase delta	Cgl	CAGL0C04455g
Complex V, F-type ATPase delta	Pst	Picst369779
Complex V, F-type ATPase delta	Sro	Sporo19237

Complex V, F-type ATPase delta Cne		Q55X25	
Complex V, F-type ATPase delta Sja		SJAG_03114	
Complex V, F-type ATPase delta Cim		CIMG_04799	
Complex V, F-type ATPase delta Ppl		Pospl1116143 || Pospl162368	
Complex V, F-type ATPase delta Acl		ACLA_031300	
Complex V, F-type ATPase delta Spa		protSpa5580	
Complex V, F-type ATPase delta Lel		LELG_05257	
Complex V, F-type ATPase delta Skl		protSkl1124	
Complex V, F-type ATPase delta Hsa		ENSP00000215375	
Complex V, F-type ATPase delta Ago		NP_984853.1	
Complex V, F-type ATPase delta Cdu		protCdu6481	
Complex V, F-type ATPase delta Cgu		PGUG_05725.1	
Complex V, F-type ATPase delta Bde		BDEG_05614	
Complex V, F-type ATPase delta Cgo		CHG02897.1	
Complex V, F-type ATPase delta Ctr		CTRG_05797.3	
Complex V, F-type ATPase delta Afu		Q4WKB0	
Complex V, F-type ATPase delta Uma		UM01103.1	
Complex V, F-type ATPase delta Spb		Q9P6R6	
Complex V, F-type ATPase delta Ath		Q96252	
Complex V, F-type ATPase delta Pbl		Phybl158473 || Phybl140164	
Complex V, F-type ATPase delta Cci		CC1G_01940.1	
Complex V, F-type ATPase delta Clu		CLUG_03476.1	
Complex V, F-type ATPase delta Pan		protPan2908	
Complex V, F-type ATPase delta Smi		protSmi4647	
Complex V, F-type ATPase delta Cal		orf19.7678	
Complex V, F-type ATPase delta Tre		Trire233586	
Complex V, F-type ATPase delta Pch		Phchr1134058	
Complex V, F-type ATPase delta Ssc		SS1G_07055.1	
Complex V, F-type ATPase delta Kpo		Kpol_1053.1	
Complex V, F-type ATPase delta Kwa		Kwal_27.11023	
Complex V, F-type ATPase delta Sno		SNU00319.1	
Complex V, F-type ATPase delta Sba		Sbay_4.5	
Complex V, F-type ATPase delta Ncr		(NCU00385.2)	
Complex V, F-type ATPase delta Ure		UREG_06717.1	
Complex V, F-type ATPase delta Ate		ATEG_02525.1	
Complex V, F-type ATPase delta Lbi		Lacbi1178018	
Complex V, F-type ATPase epsilon		Sba	Sbay_90.37
Complex V, F-type ATPase epsilon		Cgl	CAGL0A01111g
Complex V, F-type ATPase epsilon		Ago	NP_983424.1
Complex V, F-type ATPase epsilon		Kwa	Kwal_56.22333
Complex V, F-type ATPase epsilon		Sce	YPL271W
Complex V, F-type ATPase epsilon		Skl	protSkl3981
Complex V, F-type ATPase epsilon		Kla	XP_453106.1
Complex V, F-type ATPase epsilon		Kpo	Kpol_1045.82
Complex V, F-type ATPase f	Sca	Scas_632.3	
Complex V, F-type ATPase f	Cgo	CHG03792.1	
Complex V, F-type ATPase f	Nfi	NFIA_082230	
Complex V, F-type ATPase f	Fox	FOXG_10433	
Complex V, F-type ATPase f	Uma	UM05140.1	
Complex V, F-type ATPase f	Mfi	Mycfi150112	
Complex V, F-type ATPase f	Acl	ACLA_089190	
Complex V, F-type ATPase f	Ure	UREG_00730.1	
Complex V, F-type ATPase f	Spa	protSpa1529	
Complex V, F-type ATPase f	Nha	Necha261885	
Complex V, F-type ATPase f	Tre	Trire245153	
Complex V, F-type ATPase f	Hca	HCAG_00973.1	
Complex V, F-type ATPase f	Cgl	CAGL0A03036g	
Complex V, F-type ATPase f	Afl	AFL2G_09299	
Complex V, F-type ATPase f	Spb	O94377	
Complex V, F-type ATPase f	Skl	protSkl3236	
Complex V, F-type ATPase f	Smi	protSmi2669	
Complex V, F-type ATPase f	Ago	NP_983605.1	
Complex V, F-type ATPase f	Pbl	Phybl135541 || Phybl167941	
Complex V, F-type ATPase f	Lbi	Lacbi1295540	
Complex V, F-type ATPase f	Ang	A2QXL5	
Complex V, F-type ATPase f	Ssc	SS1G_13869.1	
Complex V, F-type ATPase f	Fve	FVEG_09087	
Complex V, F-type ATPase f	Sce	YDR377W	
Complex V, F-type ATPase f	Cne	Q55WE6	
Complex V, F-type ATPase f	Lel	LELG_00041	
Complex V, F-type ATPase f	Ppl	Pospl138386	
Complex V, F-type ATPase f	Kpo	Kpol_440.1	
Complex V, F-type ATPase f	Afu	Q4WHG3	
Complex V, F-type ATPase f	Sro	Sporo126958	
Complex V, F-type ATPase f	Cci	CC1G_03110.1	
Complex V, F-type ATPase f	Mgr	MGG_04135.5	
Complex V, F-type ATPase f	Sja	SJAG_00842	
Complex V, F-type ATPase f	Ate	ATEG_06738.1	
Complex V, F-type ATPase f	Ror	RO3G_01118.1 || RO3G_09471.1	

Complex V, F-type ATPase f	Pch	Phchr18514
Complex V, F-type ATPase f	Pst	Picst330875
Complex V, F-type ATPase f	Ncr	(NCU05220.2)
Complex V, F-type ATPase f	Ctr	CTRG_01055.3
Complex V, F-type ATPase f	Sku	protSku884
Complex V, F-type ATPase f	Yli	Q6C9E6
Complex V, F-type ATPase f	Gze	Q4IJY5
Complex V, F-type ATPase f	Sba	Sbay_20.63
Complex V, F-type ATPase f	Bci	BC1G_13851.1
Complex V, F-type ATPase f	Cdu	protCdu2311
Complex V, F-type ATPase f	Sno	SNU08965.1
Complex V, F-type ATPase f	Cim	CIMG_00753
Complex V, F-type ATPase f	Cal	orf19.7509.1
Complex V, F-type ATPase f	Dha	Q6BR89
Complex V, F-type ATPase f	Clu	CLUG_02642.1
Complex V, F-type ATPase G	Ago	NP_982466.1
Complex V, F-type ATPase G	Cgu	PGUG_00420.1
Complex V, F-type ATPase G	Clu	CLUG_04608.1
Complex V, F-type ATPase G	Smi	protSmi2398
Complex V, F-type ATPase G	Sku	protSku643
Complex V, F-type ATPase G	Skl	protSkl2223
Complex V, F-type ATPase G	Sca	Scas_690.27
Complex V, F-type ATPase G	Ror	RO3G_05370.1
Complex V, F-type ATPase G	Kpo	Kpol_1023.52
Complex V, F-type ATPase G	Cgl	CAGL0F08987g
Complex V, F-type ATPase G	Ncr	(NCU05118.2)
Complex V, F-type ATPase G	Dha	Q6BKC3
Complex V, F-type ATPase G	Ctr	CTRG_01696.3
Complex V, F-type ATPase G	Cci	CC1G_11571.1
Complex V, F-type ATPase G	Cim	CIMG_07768
Complex V, F-type ATPase G	Lbi	Lacbi1149931
Complex V, F-type ATPase G	Cne	Q55QQ8
Complex V, F-type ATPase G	Afl	AFL2G_07907
Complex V, F-type ATPase G	Lel	LELG_02481
Complex V, F-type ATPase G	Cdu	protCdu3734
Complex V, F-type ATPase G	Uma	UM04345.1
Complex V, F-type ATPase G	Spa	protSpa3580
Complex V, F-type ATPase G	Bde	BDEG_00535
Complex V, F-type ATPase G	Pst	Picst361950
Complex V, F-type ATPase G	Cal	orf19.1866
Complex V, F-type ATPase G	Mgr	MGG_04716.5
Complex V, F-type ATPase G	Kla	XP_452674.1
Complex V, F-type ATPase G	Sce	YHR039C-A
Complex V, F-type ATPase G	Hsa	ENSP00000259415 || ENSP00000281087
Complex V, F-type ATPase gamma	Pst	Picst391482
Complex V, F-type ATPase gamma	Ate	ATEG_02551.1
Complex V, F-type ATPase gamma	Aor	Q2URN9
Complex V, F-type ATPase gamma	Cal	orf19.3223
Complex V, F-type ATPase gamma	Cgu	PGUG_00316.1
Complex V, F-type ATPase gamma	Ctr	CTRG_00957.3
Complex V, F-type ATPase gamma	Pan	protPan6453
Complex V, F-type ATPase gamma	Hca	HCAG_04799.1
Complex V, F-type ATPase gamma	Uma	UM05090.1
Complex V, F-type ATPase gamma	Ure	UREG_06695.1
Complex V, F-type ATPase gamma	Ncr	(NCU09119.2)
Complex V, F-type ATPase gamma	Spa	protSpa1040
Complex V, F-type ATPase gamma	Lbi	Lacbi1178493
Complex V, F-type ATPase gamma	Hsa	ENSP00000349142
Complex V, F-type ATPase gamma	Pch	Phchr1128301
Complex V, F-type ATPase gamma	Fve	FVEG_05112
Complex V, F-type ATPase gamma	Nha	Necha260177
Complex V, F-type ATPase gamma	Cgo	CHG01616.1
Complex V, F-type ATPase gamma	Ang	A2Q8K1
Complex V, F-type ATPase gamma	Gze	Q4I7D3
Complex V, F-type ATPase gamma	Kwa	Kwal_56.22929
Complex V, F-type ATPase gamma	Sku	protSku2366
Complex V, F-type ATPase gamma	Afl	AFL2G_00733
Complex V, F-type ATPase gamma	Clu	CLUG_03945.1
Complex V, F-type ATPase gamma	Kla	XP_451839.1
Complex V, F-type ATPase gamma	Bde	BDEG_07357
Complex V, F-type ATPase gamma	Fox	FOXG_01951
Complex V, F-type ATPase gamma	Nfi	NFIA_021150
Complex V, F-type ATPase gamma	Ror	RO3G_06170.1 || RO3G_00424.1
Complex V, F-type ATPase gamma	Sca	Scas_718.64
Complex V, F-type ATPase gamma	Pgr	PGTG_10707
Complex V, F-type ATPase gamma	Acl	ACLA_031110
Complex V, F-type ATPase gamma	Sro	Sporo15332
Complex V, F-type ATPase gamma	Yli	Q6C338
Complex V, F-type ATPase gamma	Kpo	Kpol_1036.8

Complex V, F-type ATPase gamma	Ath	Q01909 || Q01908 || Q96250
Complex V, F-type ATPase gamma	Cdu	protCdu2370
Complex V, F-type ATPase gamma	Tre	Trire2105623
Complex V, F-type ATPase gamma	Spb	O74754
Complex V, F-type ATPase gamma	Pca	protPca1969 || protPca1389
Complex V, F-type ATPase gamma	Pbl	Phybl136806 || Phybl179462
Complex V, F-type ATPase gamma	Ppl	Pospl1112306
Complex V, F-type ATPase gamma	Cim	CIMG_04823
Complex V, F-type ATPase gamma	Mfi	Mycfi187963
Complex V, F-type ATPase gamma	Afu	Q4WK69
Complex V, F-type ATPase gamma	Cgl	CAGL0I04686g
Complex V, F-type ATPase gamma	Ani	XP_657856.1
Complex V, F-type ATPase gamma	Smi	protSmi1167
Complex V, F-type ATPase gamma	Dha	Q6BKM5
Complex V, F-type ATPase gamma	Sja	SJAG_00438
Complex V, F-type ATPase gamma	Ssc	SS1G_10016.1
Complex V, F-type ATPase gamma	Sba	Sbay_4.28
Complex V, F-type ATPase gamma	Cci	CC1G_09510.1
Complex V, F-type ATPase gamma	Lel	LELG_00176
Complex V, F-type ATPase gamma	Cne	Q55SW7
Complex V, F-type ATPase gamma	Bci	BC1G_07233.1
Complex V, F-type ATPase gamma	Skl	protSkl2995
Complex V, F-type ATPase gamma	Sno	SNU04656.1
Complex V, F-type ATPase gamma	Mgr	MGG_09916.5
Complex V, F-type ATPase gamma	Ago	NP_983228.1
Complex V, F-type ATPase gamma	Sce	YBR039W
Complex V, F-type ATPase H	Spa	protSpa488
Complex V, F-type ATPase H	Sca	Scas_586.7
Complex V, F-type ATPase H	Mfi	Mycfi158041
Complex V, F-type ATPase H	Hca	HCAG_08801.1
Complex V, F-type ATPase H	Pst	Picst332314
Complex V, F-type ATPase H	Kla	XP_452431.1 || XP_451199.1
Complex V, F-type ATPase H	Sba	Sbay_63.80
Complex V, F-type ATPase H	Sku	protSku4955
Complex V, F-type ATPase H	Ssc	SS1G_01127.1
Complex V, F-type ATPase H	Ure	UREG_07397.1
Complex V, F-type ATPase H	Cgl	CAGL0I00572g
Complex V, F-type ATPase H	Fox	FOXG_00583
Complex V, F-type ATPase H	Gze	Q4IQN7
Complex V, F-type ATPase H	Cim	CIMG_03626
Complex V, F-type ATPase H	Smi	protSmi768
Complex V, F-type ATPase H	Yli	Q6C2V6
Complex V, F-type ATPase H	Sno	SNU07915.1
Complex V, F-type ATPase H	Cal	orf19.5491.1
Complex V, F-type ATPase H	Sce	YLR295C
Complex V, F-type ATPase H	Bci	BC1G_14868.1
Complex V, F-type ATPase H	Ago	NP_986961.2
Complex V, F-type ATPase H	Fve	FVEG_00930
Complex V, F-type ATPase H	Skl	protSkl3556
Complex V, F-type ATPase H	Kwa	Kwal_56.23371
Complex V, F-type ATPase H	Lel	LELG_01883
Complex V, F-type ATPase H	Mgr	MGG_01250.5
Complex V, F-type ATPase H	Kpo	Kpol_1009.10
Complex V, F-type ATPase H	Dha	Q6BMW8
Complex V, F-type ATPase J	Ang	A2QNQ0
Complex V, F-type ATPase J	Cgl	CAGL0H04191g
Complex V, F-type ATPase J	Sno	SNU00321.1
Complex V, F-type ATPase J	Dha	Q6BWY1
Complex V, F-type ATPase J	Uma	UM05451.1
Complex V, F-type ATPase J	Yli	Q6C8S0
Complex V, F-type ATPase J	Kpo	Kpol_1000.27
Complex V, F-type ATPase J	Nfi	NFIA_034650
Complex V, F-type ATPase J	Ago	NP_985021.1
Complex V, F-type ATPase J	Fox	FOXG_06116
Complex V, F-type ATPase J	Cci	CC1G_10200.1
Complex V, F-type ATPase J	Fve	FVEG_03980
Complex V, F-type ATPase J	Cal	orf19.2066.1
Complex V, F-type ATPase J	Bci	BC1G_01321.1
Complex V, F-type ATPase J	Pbl	Phybl139414
Complex V, F-type ATPase J	Ssc	SS1G_09444.1
Complex V, F-type ATPase J	Tre	Trire2111027
Complex V, F-type ATPase J	Sba	Sbay_65.122
Complex V, F-type ATPase J	Kwa	Kwal_47.16642
Complex V, F-type ATPase J	Mfi	Mycfi129423
Complex V, F-type ATPase J	Acl	ACLA_092540
Complex V, F-type ATPase J	Sce	YML081C-A
Complex V, F-type ATPase J	Afu	A4D9U8
Complex V, F-type ATPase J	Kla	XP_451728.1
Complex V, F-type ATPase J	Afl	AFL2G_05268

Complex V, F-type ATPase J	Pst	Picst381568
Complex V, F-type ATPase J	Mgr	MGG_02606.5
Complex V, F-type ATPase J	Sca	Scas_703.22
Complex V, F-type ATPase J	Spb	O13931
Complex V, F-type ATPase J	Gze	Q4I028
Complex V, F-type ATPase J	Lbi	Lacbi1294671
Complex V, F-type ATPase J	Skl	protSkl5043
Complex V, F-type ATPase K	Sku	protSku2169
Complex V, F-type ATPase K	Ctr	CTRG_03516.3
Complex V, F-type ATPase K	Cal	orf19.5231.2
Complex V, F-type ATPase K	Sca	Scas_710.48d
Complex V, F-type ATPase K	Cgl	CAGL0I07969g
Complex V, F-type ATPase K	Cgu	PGUG_02752.1
Complex V, F-type ATPase K	Smi	protSmi4884
Complex V, F-type ATPase K	Cdu	protCdu5589
Complex V, F-type ATPase K	Skl	protSkl2890
Complex V, F-type ATPase K	Kpo	Kpol_1036.13
Complex V, F-type ATPase K	Ago	NP_984881.1
Complex V, F-type ATPase K	Lel	LELG_03606
Complex V, F-type ATPase K	Sba	Sbay_83.4
Complex V, F-type ATPase K	Sce	YOL077W-A
Complex V, F-type ATPase K	Kwa	Kwal_56.23129
Complex V, F-type ATPase K	Dha	Q6BIK4
Complex V, F-type ATPase OSCP	Acl	ACLA_071250
Complex V, F-type ATPase OSCP	Sja	SJAG_00133
Complex V, F-type ATPase OSCP	Sro	Sporo16470
Complex V, F-type ATPase OSCP	Pan	protPan12824
Complex V, F-type ATPase OSCP	Smi	protSmi2763
Complex V, F-type ATPase OSCP	Spb	O74479
Complex V, F-type ATPase OSCP	Mgr	MGG_03152.5
Complex V, F-type ATPase OSCP	Ang	A2QCR9
Complex V, F-type ATPase OSCP	Pbl	Phybl116397
Complex V, F-type ATPase OSCP	Ctr	CTRG_06190.3
Complex V, F-type ATPase OSCP	Ate	ATEG_01173.1
Complex V, F-type ATPase OSCP	Ncr	(NCU01606.2)
Complex V, F-type ATPase OSCP	Kwa	Kwal_23.5747
Complex V, F-type ATPase OSCP	Nha	Necha2102383
Complex V, F-type ATPase OSCP	Bci	BC1G_07106.1
Complex V, F-type ATPase OSCP	Cgo	CHG10215.1
Complex V, F-type ATPase OSCP	Gze	Q4IR58
Complex V, F-type ATPase OSCP	Sba	Sbay_19.126
Complex V, F-type ATPase OSCP	Ani	XP_663891.1
Complex V, F-type ATPase OSCP	Mfi	Mycfi170321
Complex V, F-type ATPase OSCP	Cim	CIMG_05216
Complex V, F-type ATPase OSCP	Dha	Q6BQG2
Complex V, F-type ATPase OSCP	Yli	Q6C9B1
Complex V, F-type ATPase OSCP	Hca	HCAG_03815.1
Complex V, F-type ATPase OSCP	Clu	CLUG_00602.1
Complex V, F-type ATPase OSCP	Kpo	Kpol_1004.74
Complex V, F-type ATPase OSCP	Ath	Q96251
Complex V, F-type ATPase OSCP	Sku	protSku4279
Complex V, F-type ATPase OSCP	Uma	UM06324.1
Complex V, F-type ATPase OSCP	Kla	XP_452662.1
Complex V, F-type ATPase OSCP	Cgu	PGUG_02610.1
Complex V, F-type ATPase OSCP	Sca	Scas_588.18
Complex V, F-type ATPase OSCP	Lel	LELG_02257
Complex V, F-type ATPase OSCP	Ure	UREG_06408.1
Complex V, F-type ATPase OSCP	Skl	protSkl886
Complex V, F-type ATPase OSCP	Sce	YDR298C
Complex V, F-type ATPase OSCP	Hsa	ENSP00000290299
Complex V, F-type ATPase OSCP	Pca	protPca3703
Complex V, F-type ATPase OSCP	Afu	Q4X0T1
Complex V, F-type ATPase OSCP	Ago	NP_982477.1
Complex V, F-type ATPase OSCP	Fox	FOXG_00768
Complex V, F-type ATPase OSCP	Pch	Phchr132922
Complex V, F-type ATPase OSCP	Pst	Picst381978
Complex V, F-type ATPase OSCP	Fve	FVEG_00747
Complex V, F-type ATPase OSCP	Spa	protSpa94
Complex V, F-type ATPase OSCP	Sno	SNU15659.1
Complex V, F-type ATPase OSCP	Cne	Q55TZ7
Complex V, F-type ATPase OSCP	Nfi	NFIA_087540
Complex V, F-type ATPase OSCP	Cdu	protCdu5012
Complex V, F-type ATPase OSCP	Aor	Q2UF30
Complex V, F-type ATPase OSCP	Bde	BDEG_03163
Complex V, F-type ATPase OSCP	Ssc	SS1G_05365.1
Complex V, F-type ATPase OSCP	Ror	RO3G_09398.1 || RO3G_14729.1
Complex V, F-type ATPase OSCP	Tre	Trire221535
Complex V, F-type ATPase OSCP	Pgr	PGTG_12123
Complex V, F-type ATPase OSCP	Afl	AFL2G_06887

Complex V, F-type ATPase OSCP	Lbi	Lacbi1313948
Complex V, F-type ATPase OSCP	Cci	CC1G_03278.1
Complex V, F-type ATPase OSCP	Cgl	CAGL0H01397g
Complex V, F-type ATPase OSCP	Cal	orf19.5419

Additional figures 1, 2 and 3

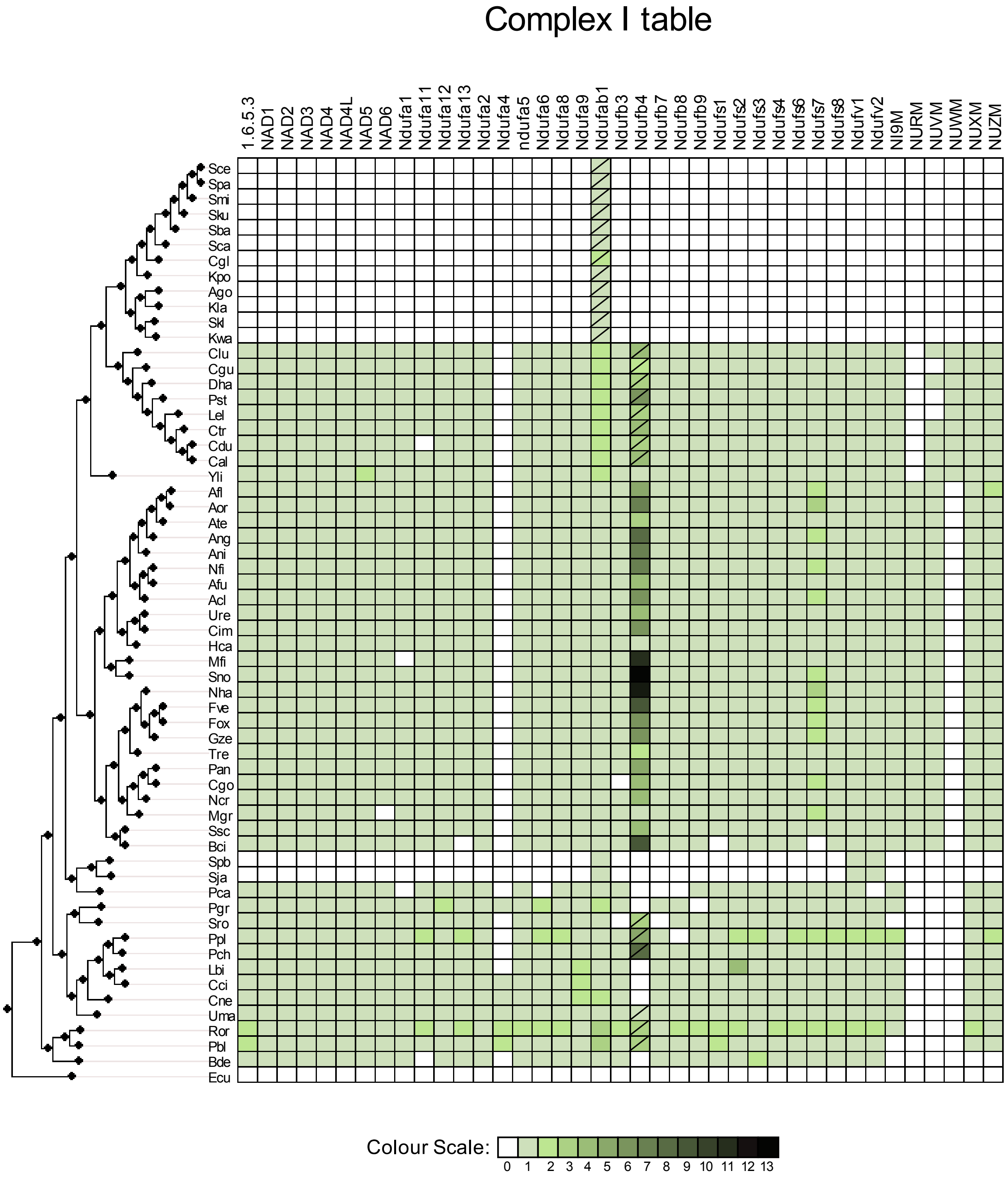
Complex I table
1.6.5.3
NAD1
NAD2
NAD3
NAD4
NAD4L
NAD5
NAD6
Ndufa1
Ndufa11
Ndufa12
Ndufa13
Ndufa2
Ndufa4
ndufa5
Ndufa6
Ndufa8
Ndufa9
Ndufab1
Ndufb3
Ndufb4
Ndufb7
Ndufb8
Ndufb9
Ndufs1
Ndufs2
Ndufs3
Ndufs4
Ndufs6
Ndufs7
Ndufs8
Ndufv1
Ndufv2
NI9M
NURM
NUVM
NUWM
NUXM
NUZM
Sce
Spa
Smi
Sku
Sba
Sca
Cgl
Kpo
Ago
Kla
Skl
Kwa
Clu
Cgu
Dha
Pst
Lel
Ctr
Cdu
Cal
Yli
Afl
Aor
Ate
Ang
Ani
Nfi
Afu
Acl
Ure
Cim
Hca
Mfi
Sno
Nha
Fve
Fox
Gze
Tre
Pan
Cgo
Ncr
Mgr
Ssc
Bci
Spb
Sja
Pca
Pgr
Sro
Ppl
Pch
Lbi
Cci
Cne
Uma
Ror
Pbl
Bde
Ecu
Colour Scale:
0 1 2 3 4 5 6 7 8 9 10 11 12 13

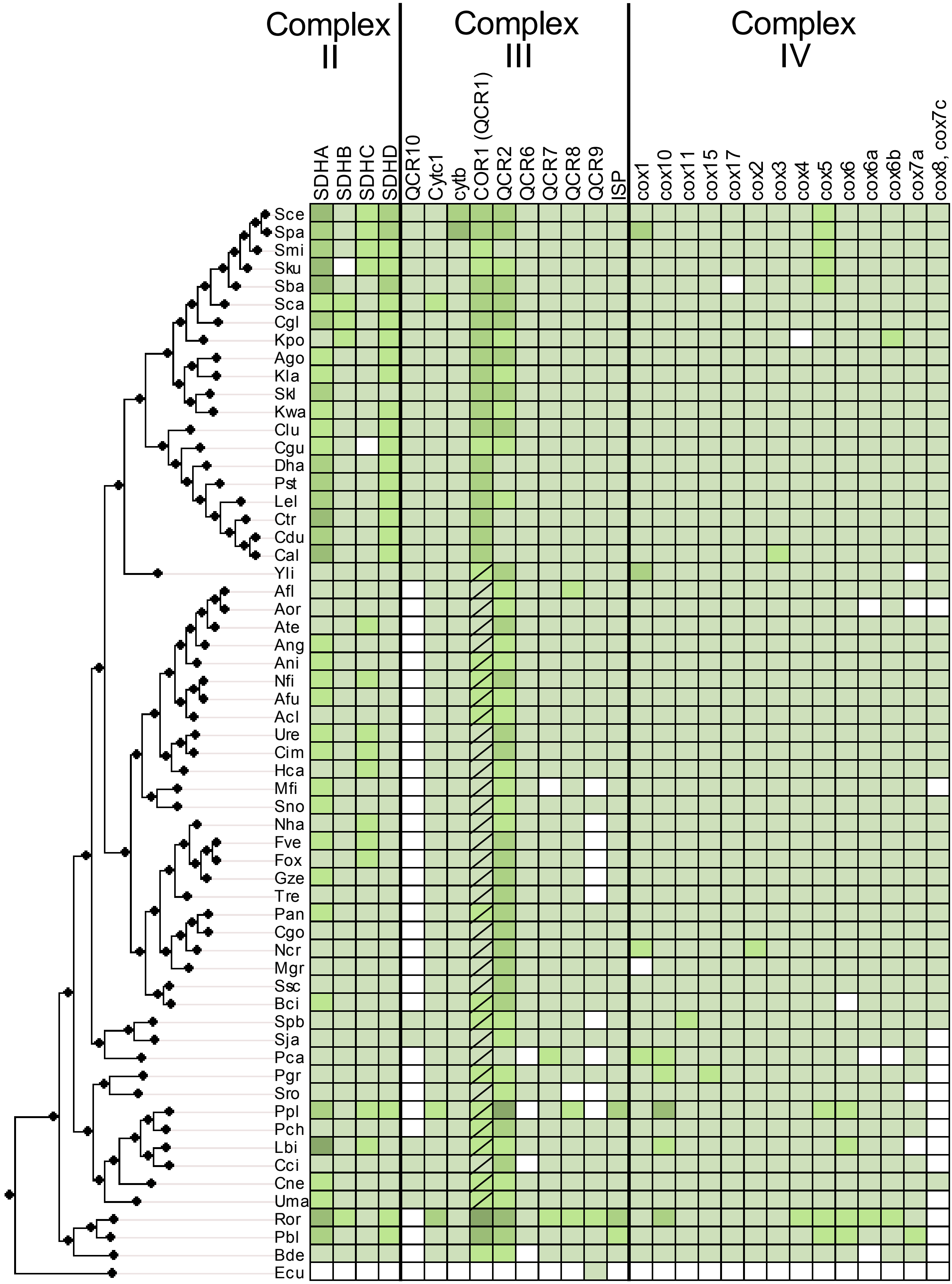
Complex II
Complex III
Complex IV
SDHA
SDHB
SDHC
SDHD
QCR10
Cytc1
cytb
COR1 (QCR1)
QCR2
QCR6
QCR7
QCR8
QCR9
ISP
cox1
cox10
cox11
cox15
cox17
cox2
cox3
cox4
cox5
cox6
cox6a
cox6b
cox7a
cox8, cox7c
Sce
Spa
Smi
Sku
Sba
Sca
Cgl
Kpo
Ago
Kla
Skl
Kwa
Clu
Cgu
Dha
Pst
Lel
Ctr
Cdu
Cal
Yli
Afl
Aor
Ate
Ang
Ani
Nfi
Afu
Acl
Ure
Cim
Hca
Mfi
Sno
Nha
Fve
Fox
Gze
Tre
Pan
Cgo
Ncr
Mgr
Ssc
Bci
Spb
Sja
Pca
Pgr
Sro
Ppl
Pch
Lbi
Cci
Cne
Uma
Ror
Pbl
Bde
Ecu

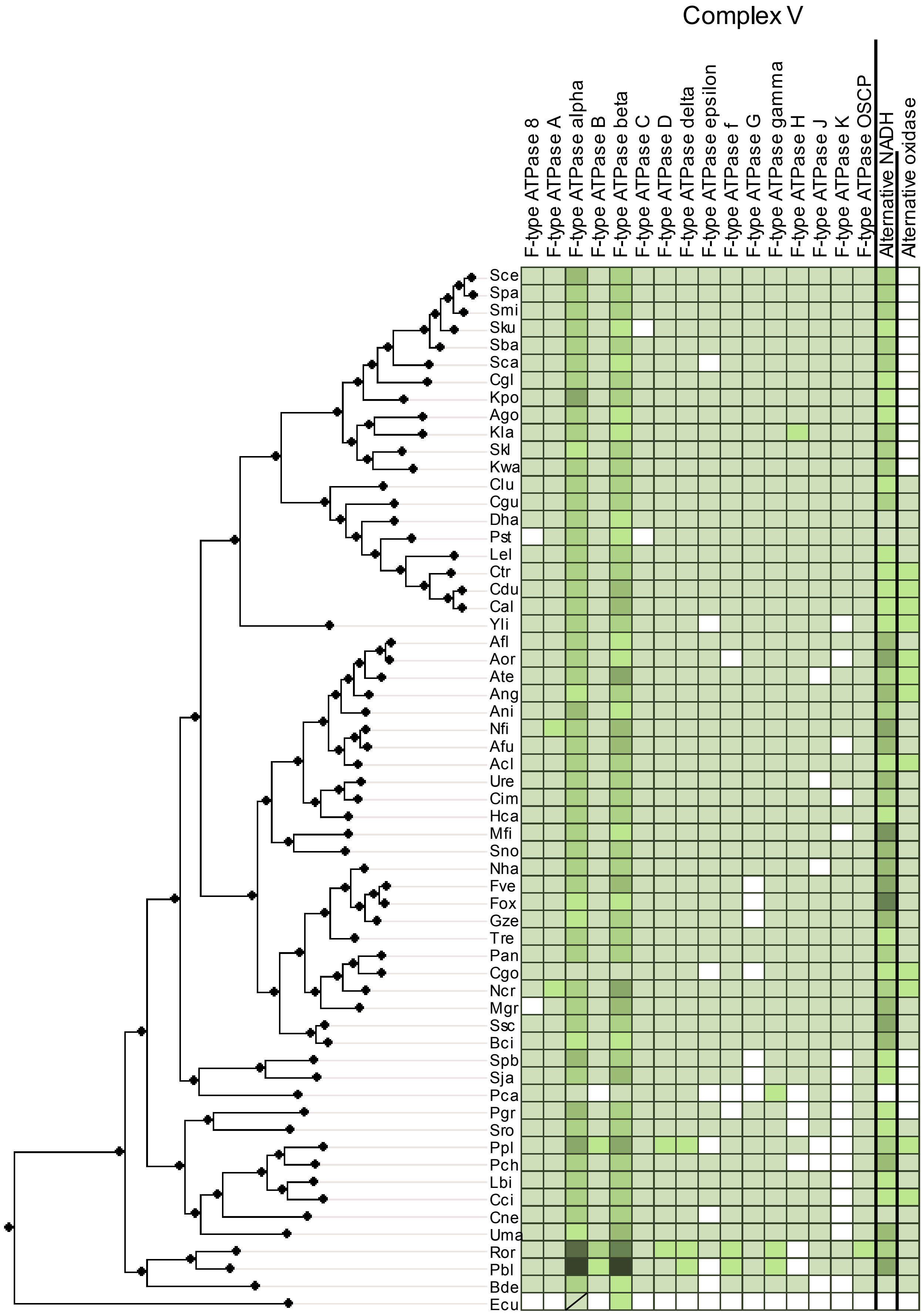
Complex V
F-type ATPase 8
F-type ATPase A
F-type ATPase alpha
F-type ATPase B
F-type ATPase beta
F-type ATPase C
F-type ATPase D
F-type ATPase delta
F-type ATPase epsilon
F-type ATPase f
F-type ATPase G
F-type ATPase gamma
F-type ATPase H
F-type ATPase J
F-type ATPase K
F-type ATPase OSCP
Alternative NADH
Alternative oxidase
Sce
Spa
Smi
Sku
Sba
Sca
Cgl
Kpo
Ago
Kla
Skl
Kwa
Clu
Cgu
Dha
Pst
Lel
Ctr
Cdu
Cal
Yli
Afl
Aor
Ate
Ang
Ani
Nfi
Afu
Acl
Ure
Cim
Hca
Mfi
Sno
Nha
Fve
Fox
Gze
Tre
Pan
Cgo
Ncr
Mgr
Ssc
Bci
Spb
Sja
Pca
Pgr
Sro
Ppl
Pch
Lbi
Cci
Cne
Uma
Ror
Pbl
Bde
Ecu

Additional figure 4

Complex I, Ndufa1

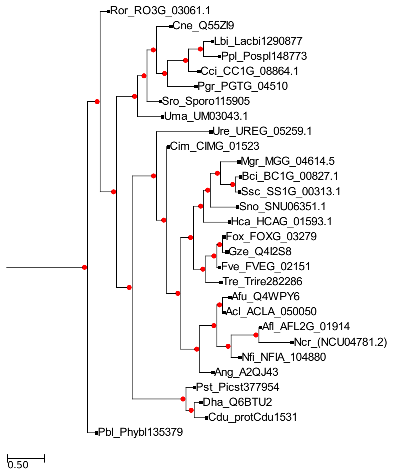

Complex I, Ndufa2

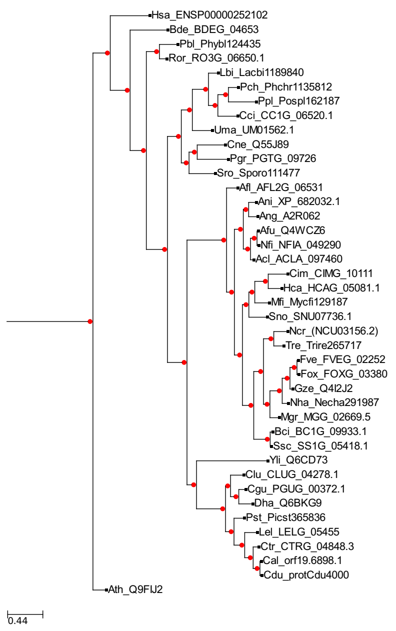

Complex I, Ndufa4

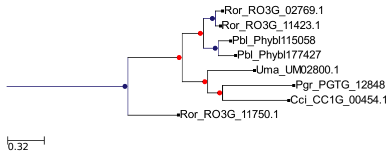

Complex I, Ndufa5

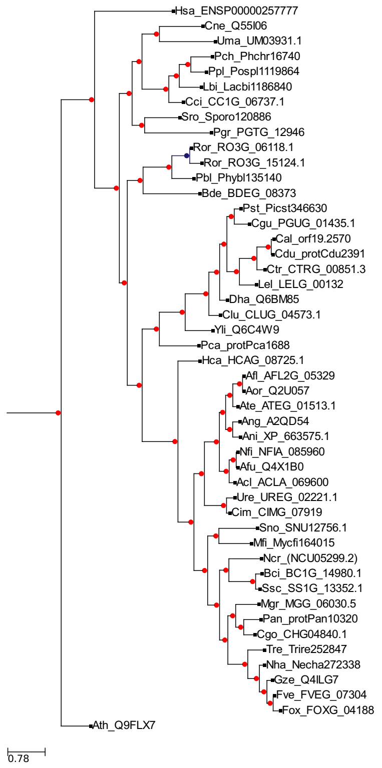

Complex I, Ndufa6

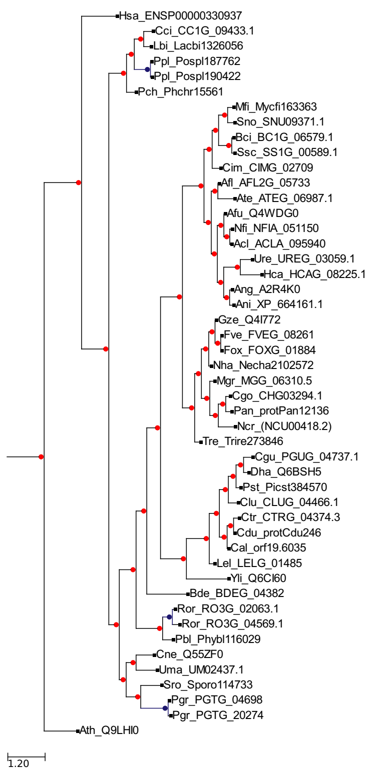

Complex I, Ndufa8

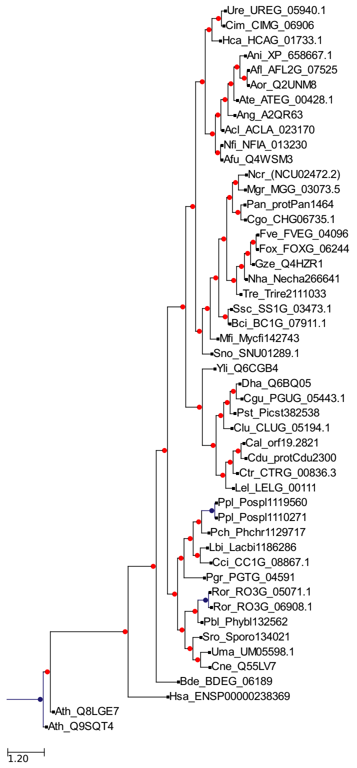

Complex I, Ndufa9

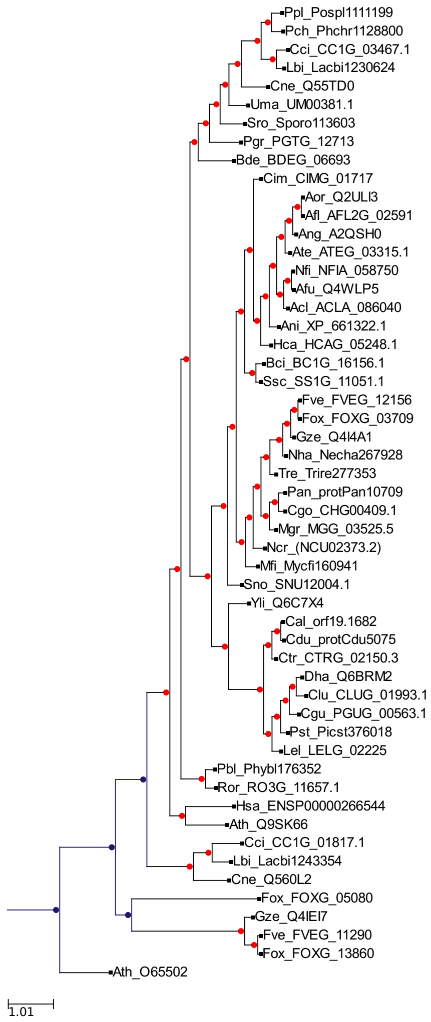

Complex I, Ndufa11

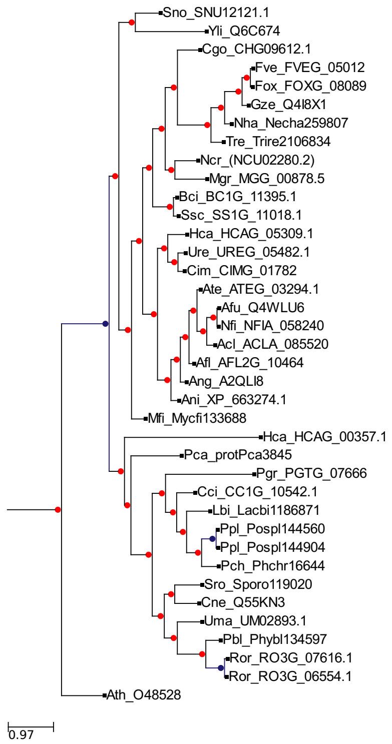

Complex I, Ndufa12

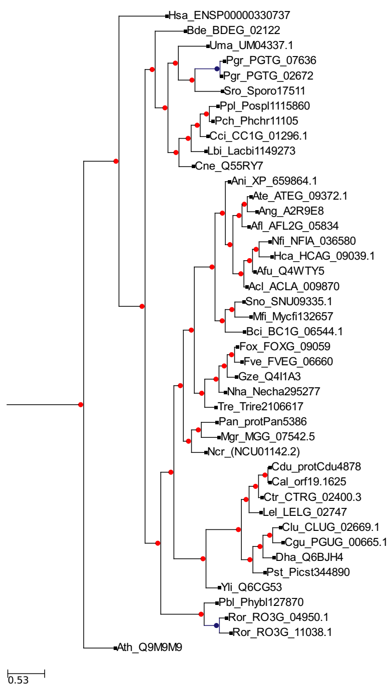

Complex I, Ndufa13

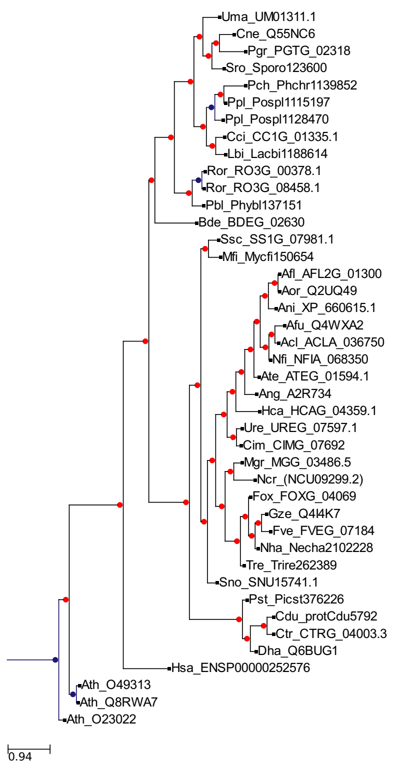

Complex I, Ndufab1

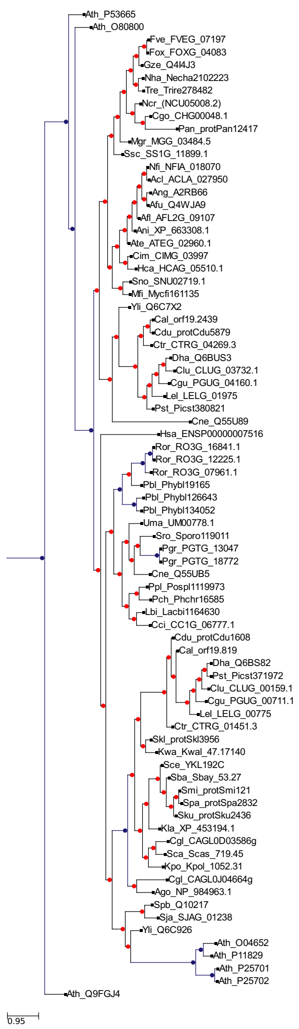

Complex I, Ndufb3

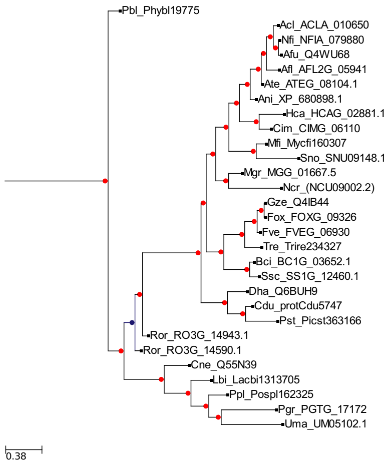

Complex I, Ndufb4

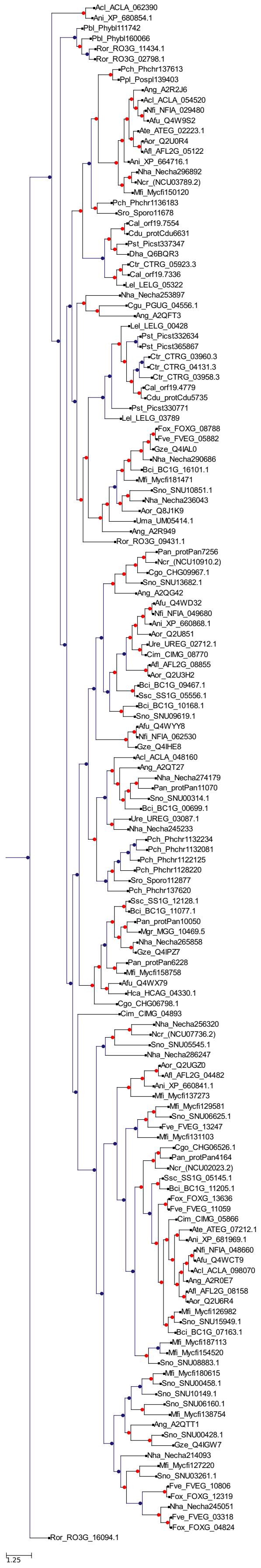

Complex I, Ndufb7

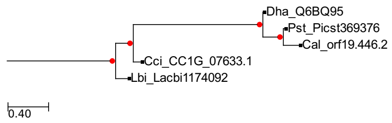

Complex I, Ndufb8

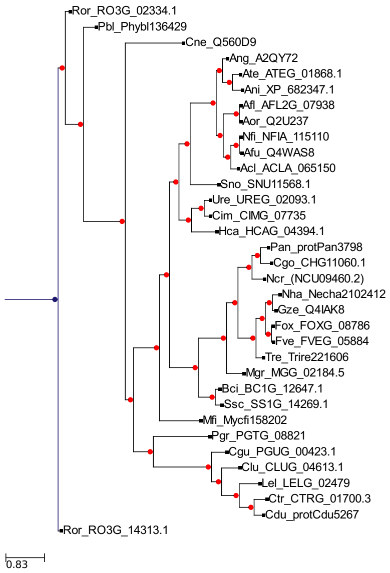

Complex I, Ndufb9

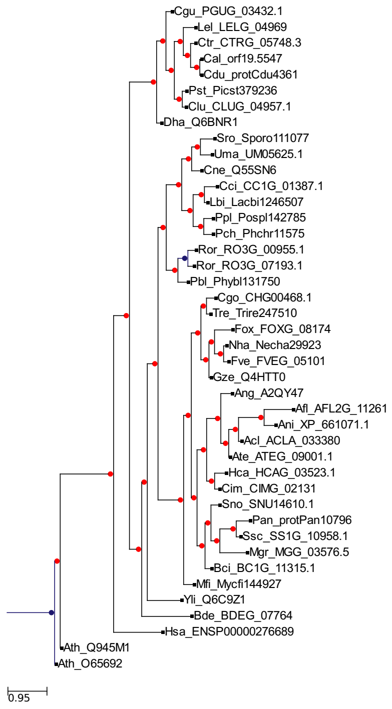

Complex I, Ndufs1

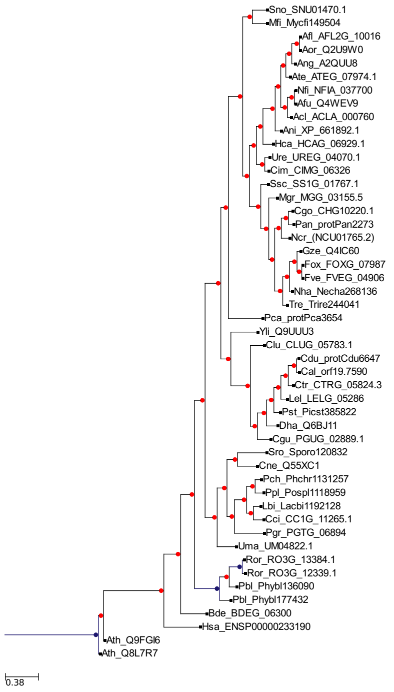

Complex I, Ndufs2

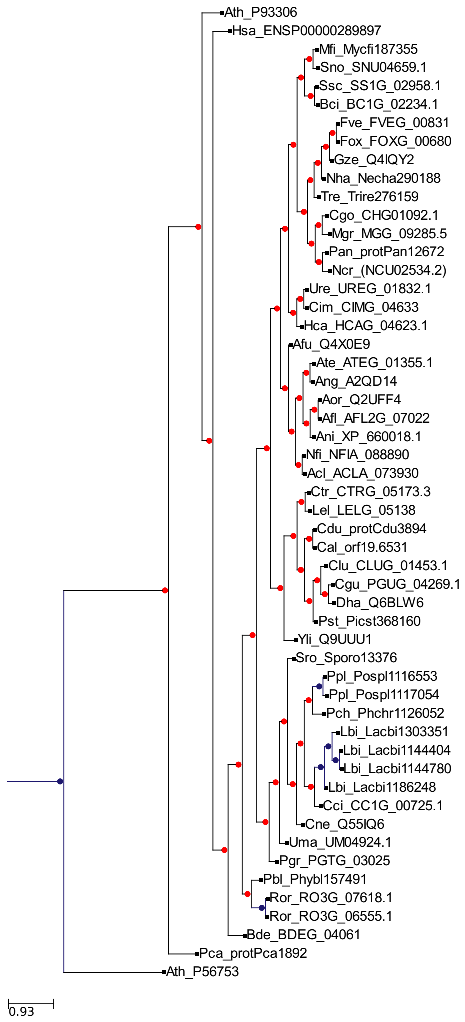

Complex I, Ndufs3

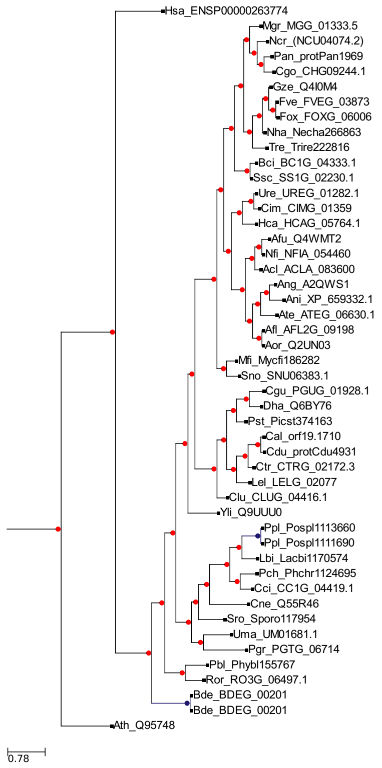

Complex I, Ndufs4

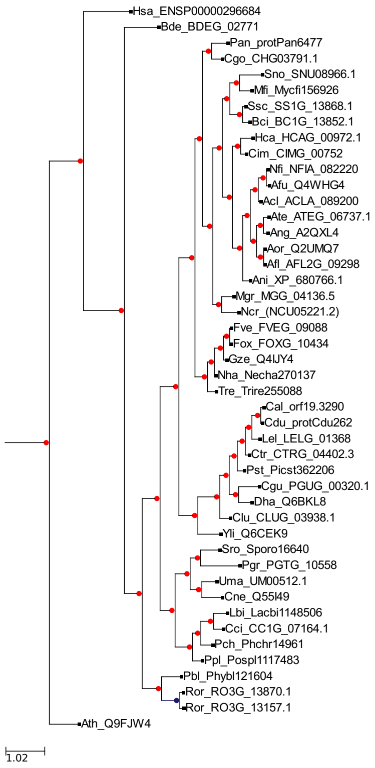

Complex I, Ndufs6

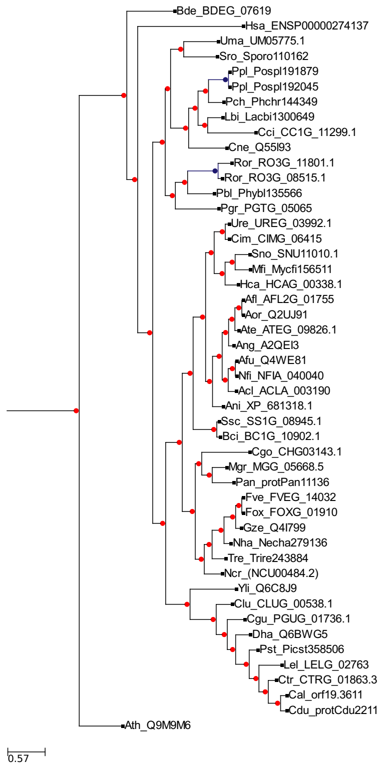

Complex I, Ndufs7

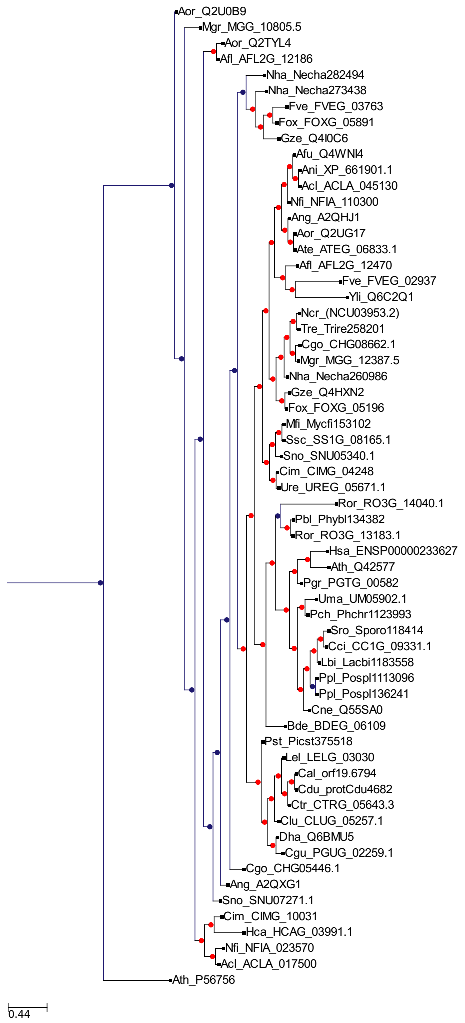

Complex I, Ndufs8

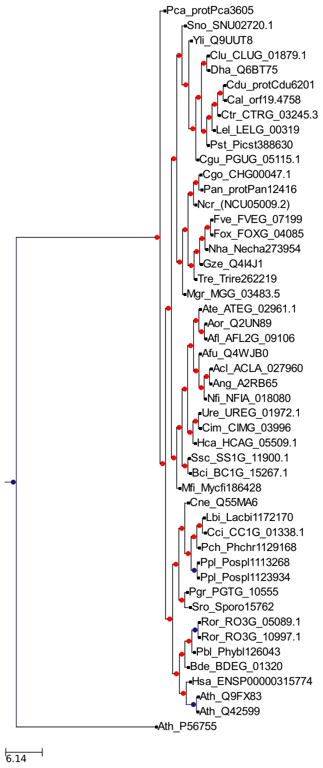

Complex I, Ndufv1

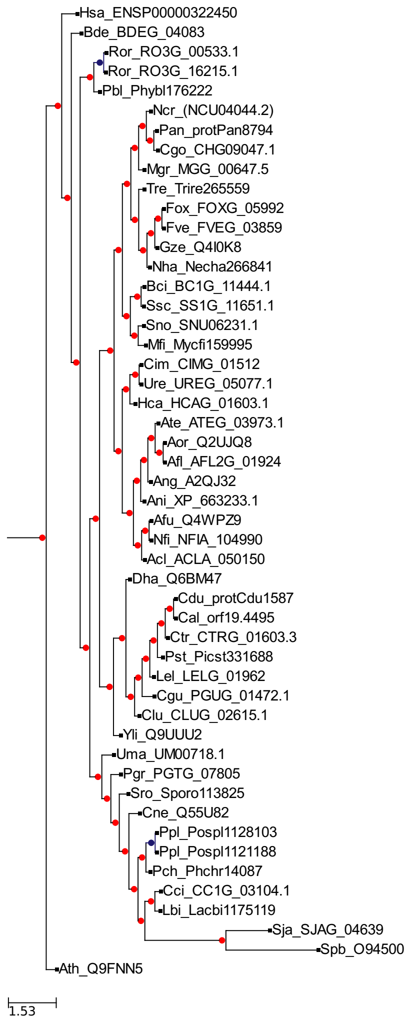

Complex I, Ndufv2

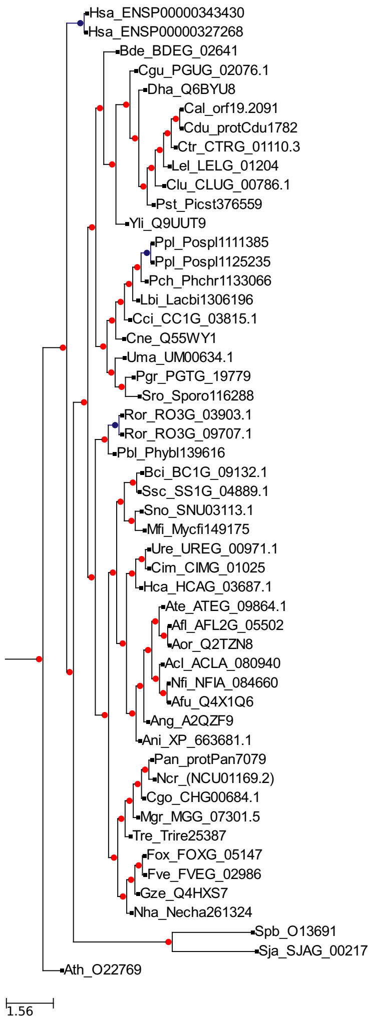

Complex I, 1.6.5.3

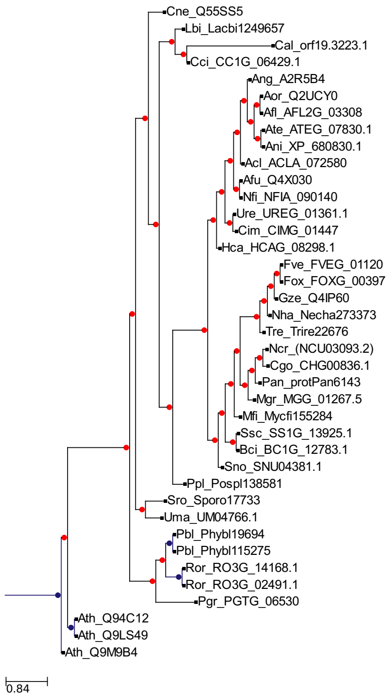

Complex I, NAD1

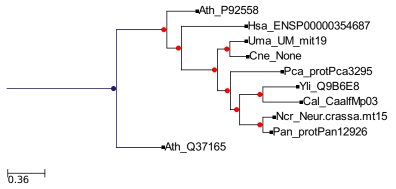

Complex I, NAD2

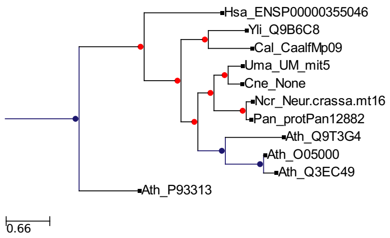

Complex I, NAD3

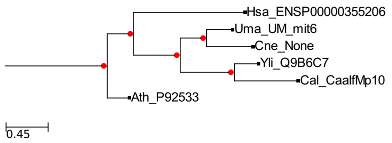

Complex I, NAD4

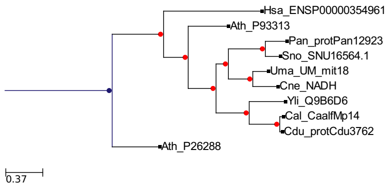

Complex I, NAD4L

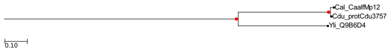

Complex I, NAD5

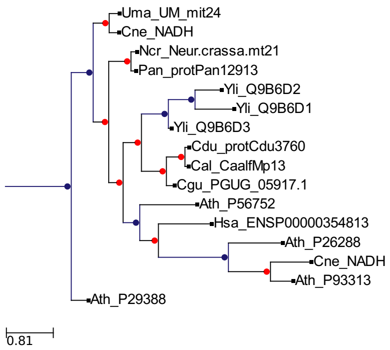

Complex I, NAD6

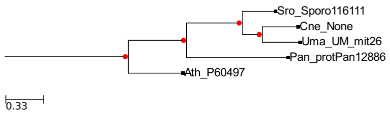

Complex I, NI9M

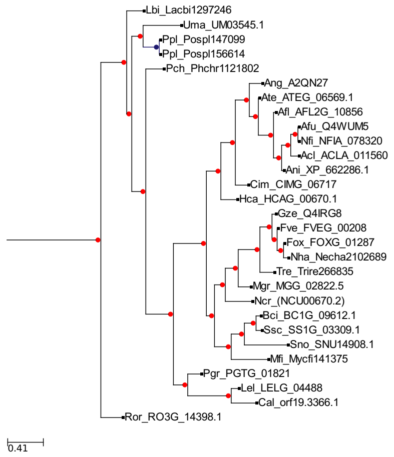

Complex I, NURM

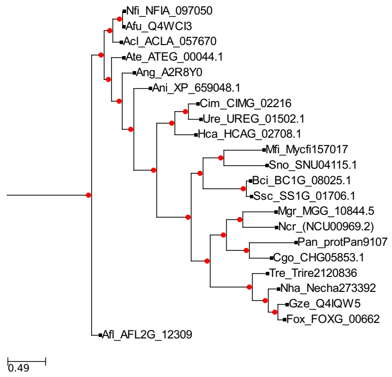

Complex I, NUVM

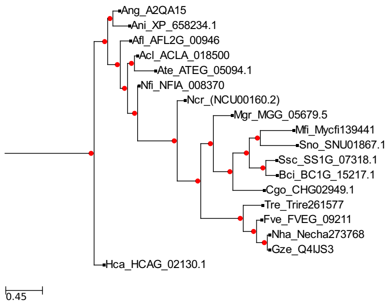

Complex I, NUWM

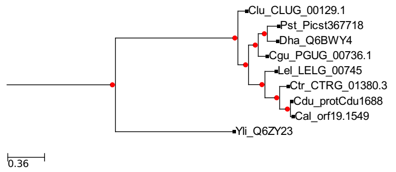

Complex I, NUXM

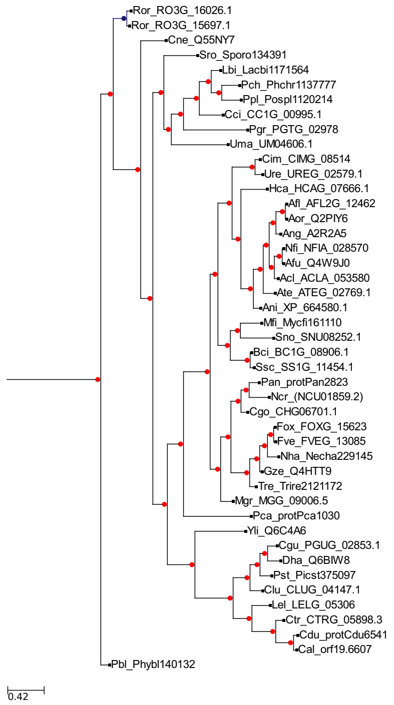

Complex I, NUZM

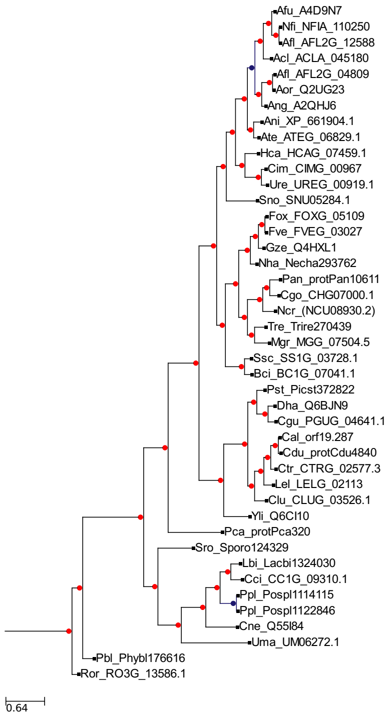

Complex II, SDHA

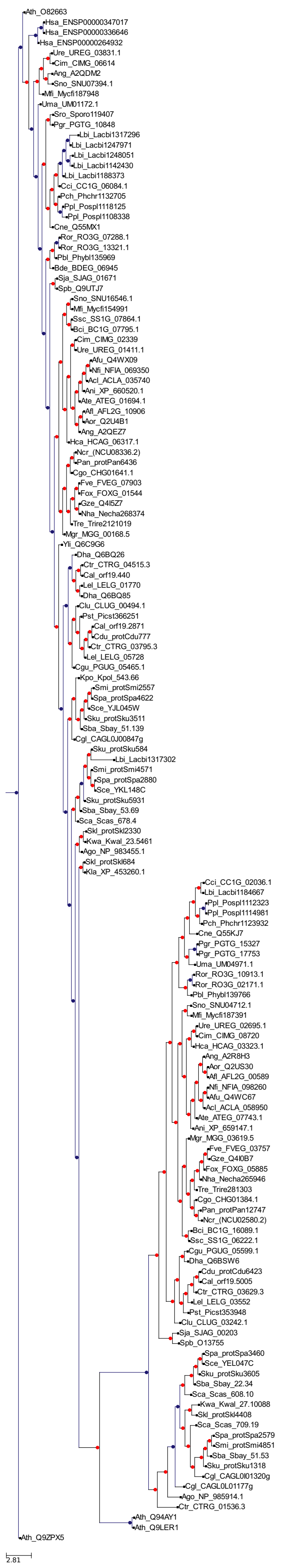

Complex II, SDHB

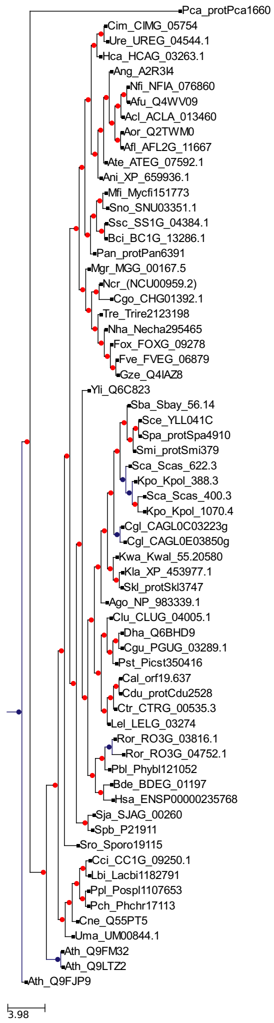

Complex II, SDHC

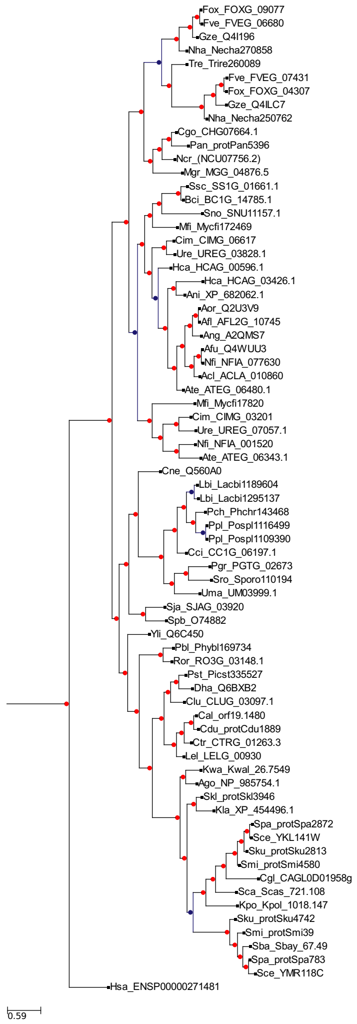

Complex II, SDHD

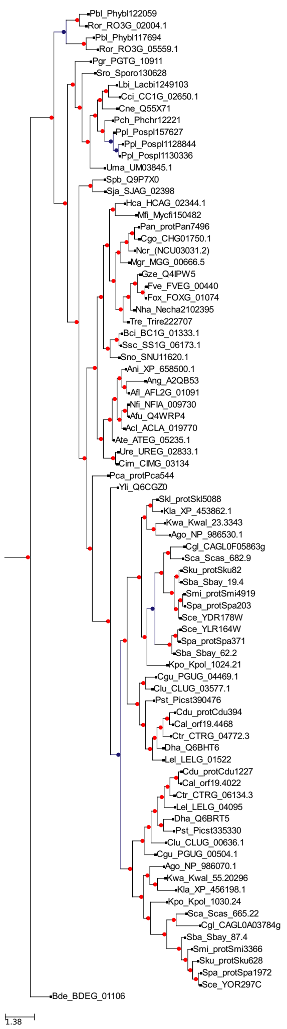

Complex III, Cytb

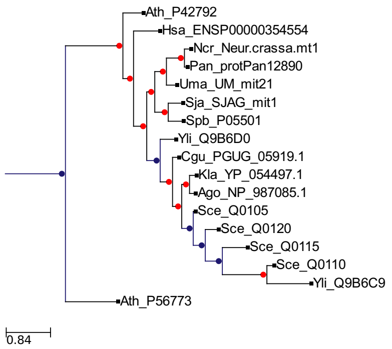

Complex III, Cytc1

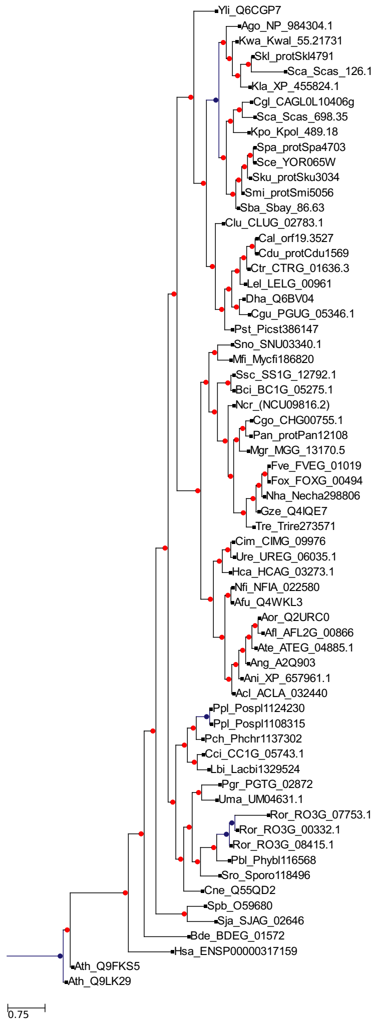

Complex III, ISP

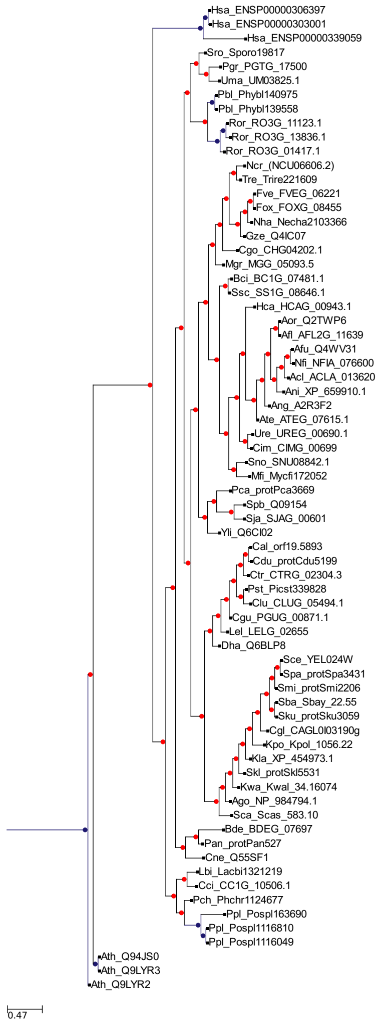

Complex III, QCR1

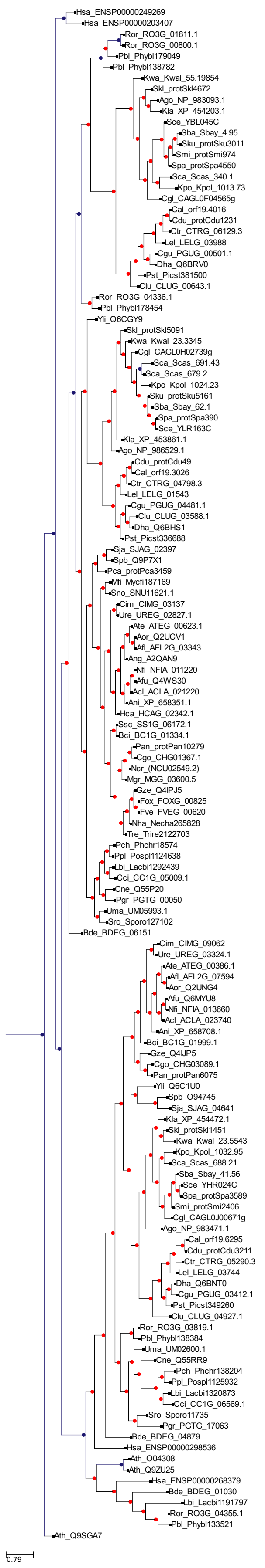

Complex III, QCR2

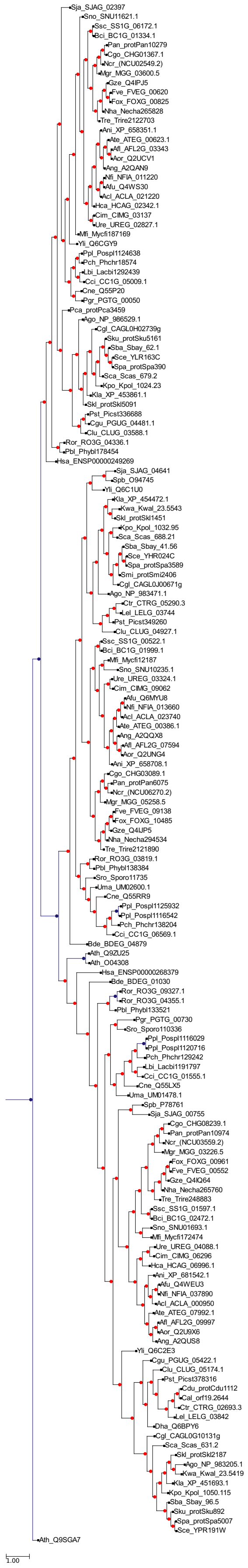

Complex III, QCR6

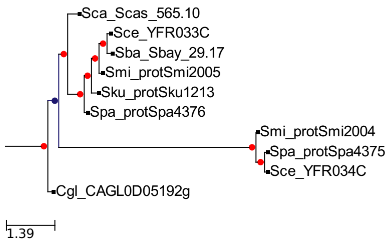

Complex III, QCR7

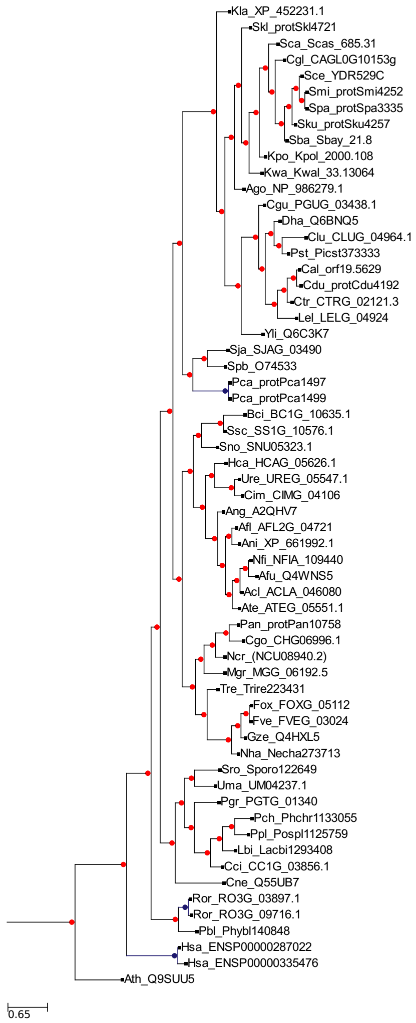

Complex III, QCR8

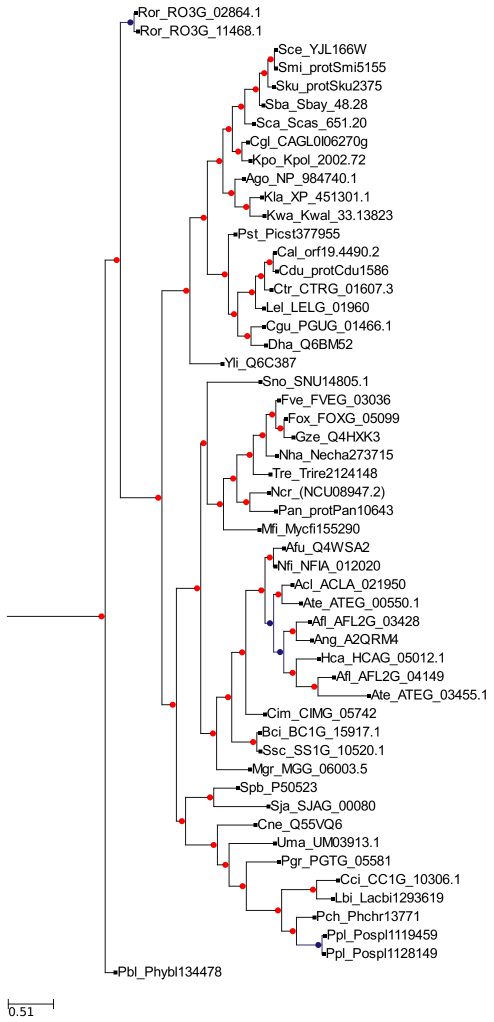

Complex III, QCR9

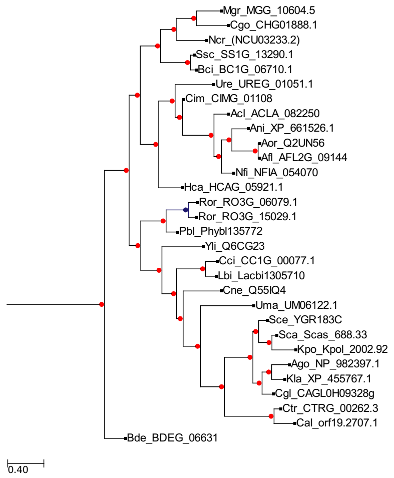

Complex III, QCR10

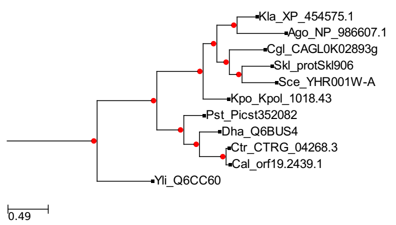

Complex IV, Cox1

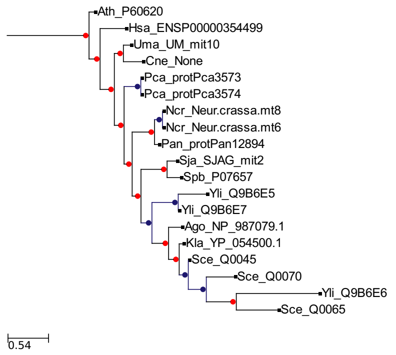

Complex IV, Cox2

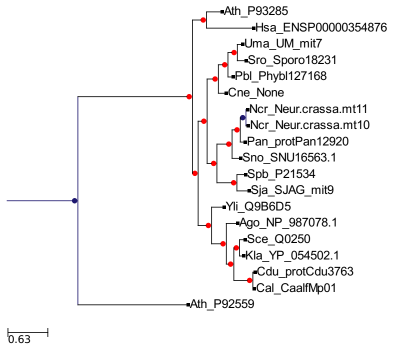

Complex IV, Cox3

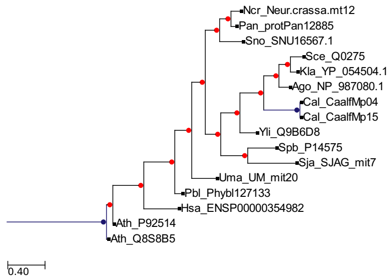

Complex IV, Cox4

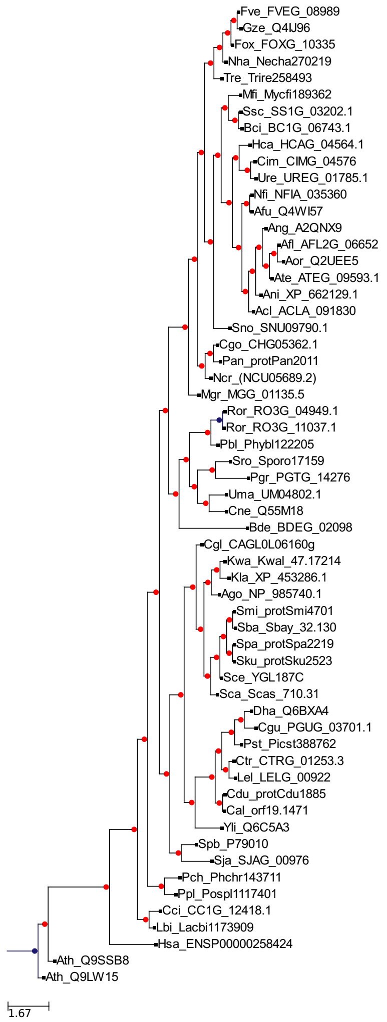

Complex IV, Cox5

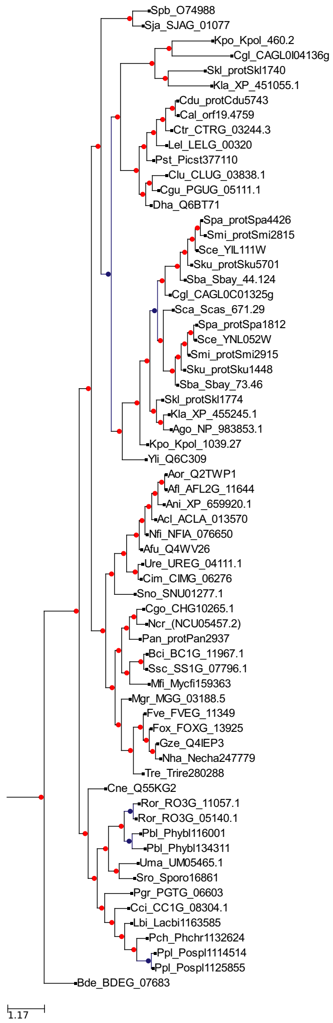

Complex IV, Cox6

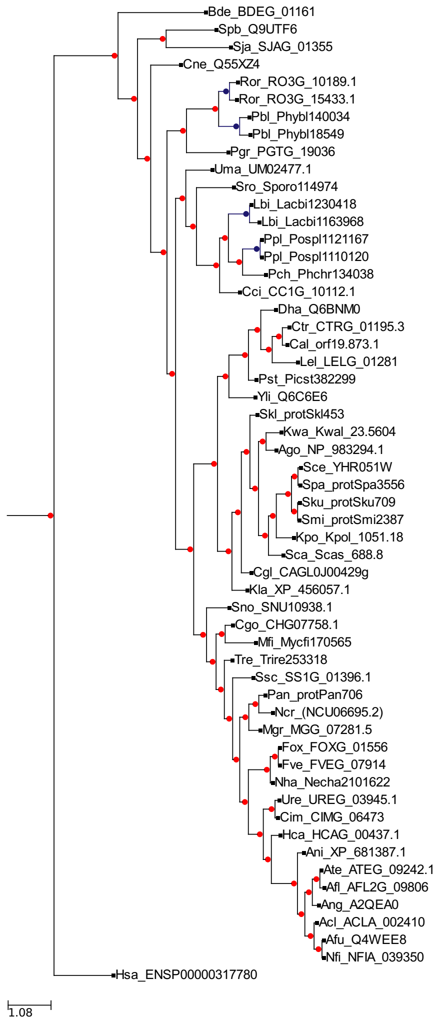

Complex IV, Cox6a

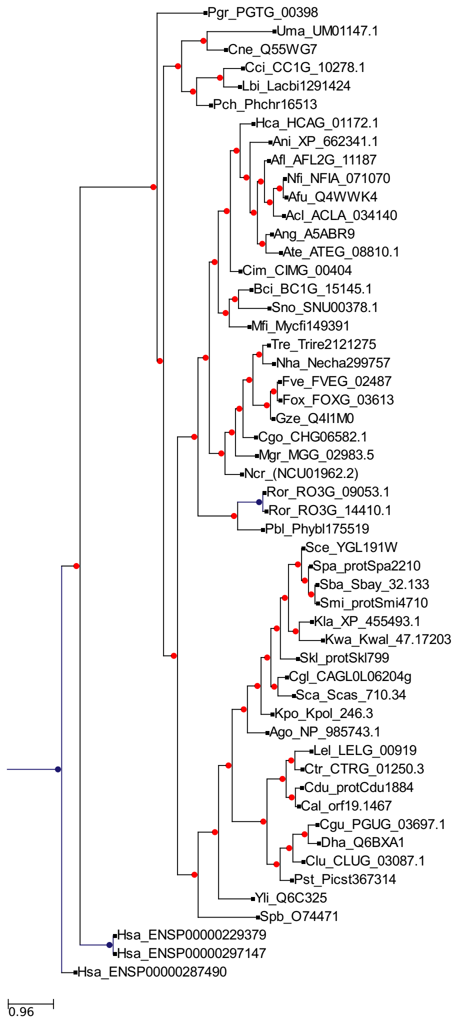

Complex IV, Cox6b

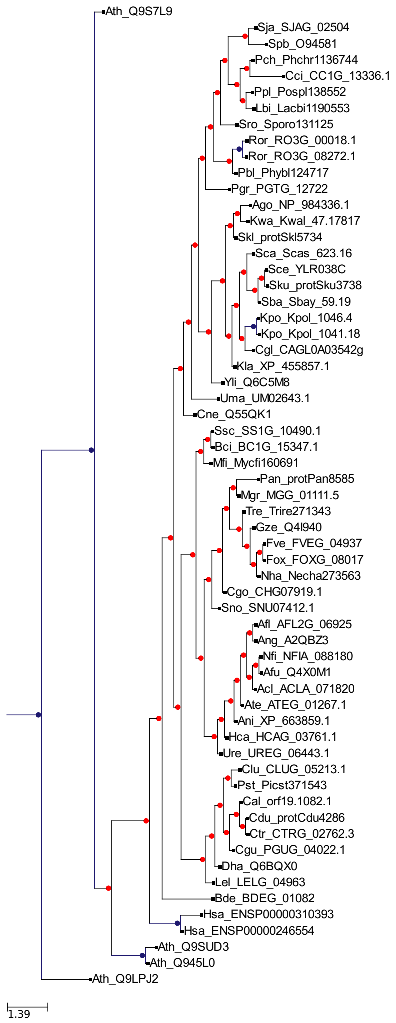

Complex IV, Cox7a

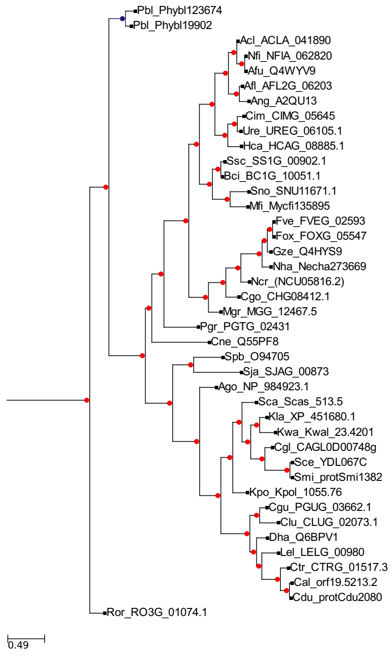

Complex IV, Cox8 / Cox7c

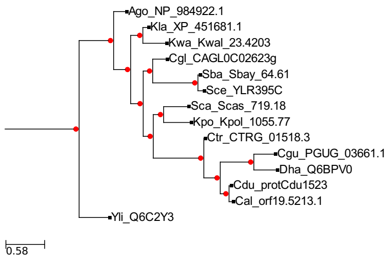

Complex IV, Cox10

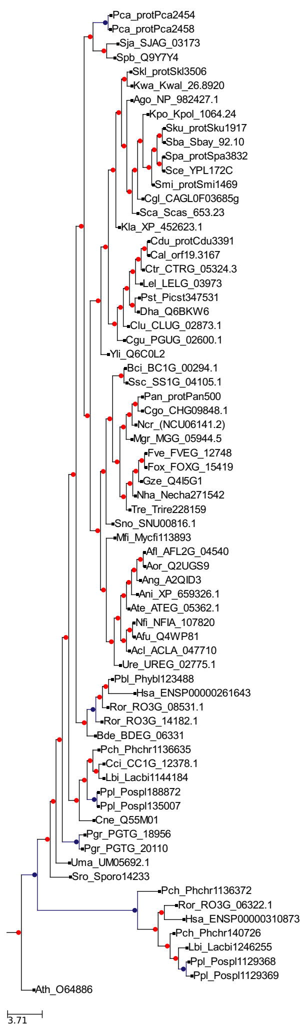

Complex IV, Cox11

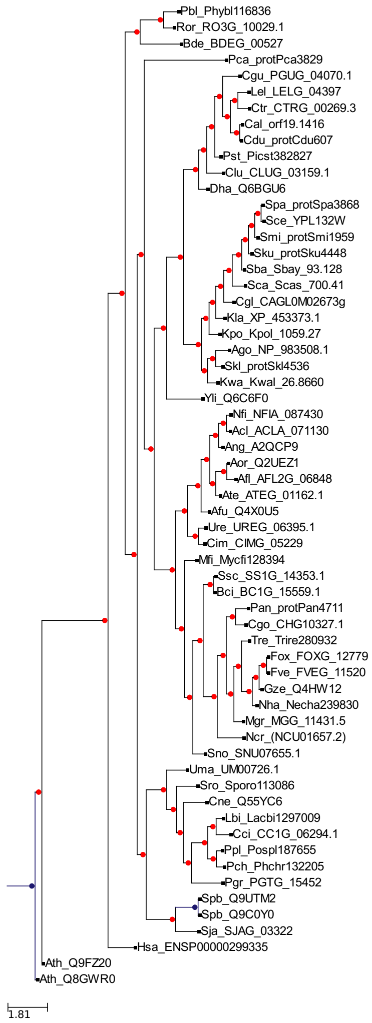

Complex IV, Cox15

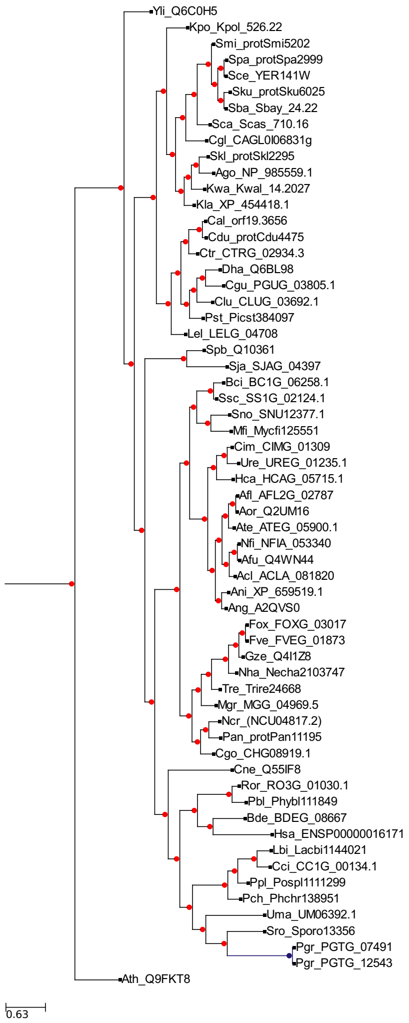

Complex IV, Cox17

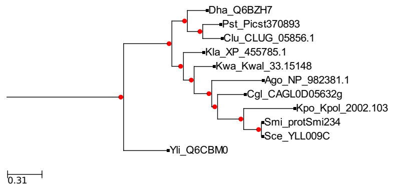

Complex V, F-type ATPase 8

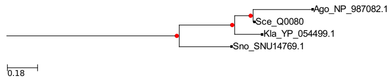

Complex V, F-type ATPase A

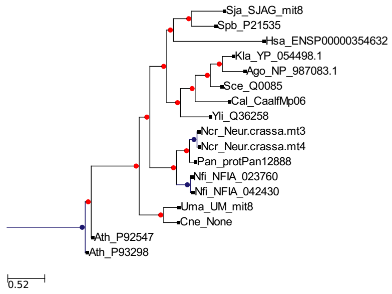

Complex V, F-type ATPase alpha

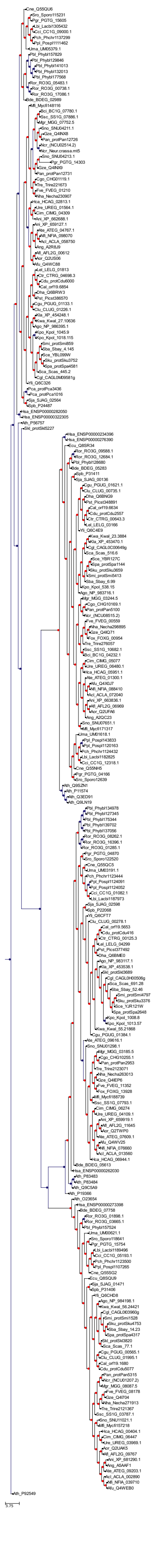

Complex V, F-type ATPase B

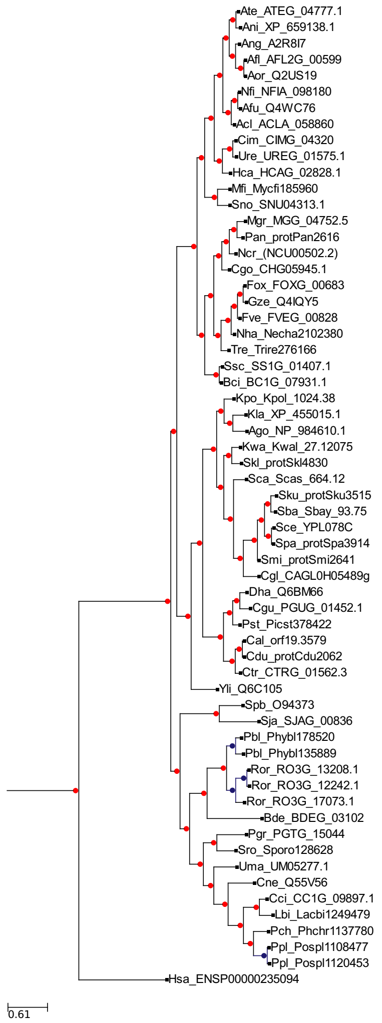

Complex V, F-type ATPase beta

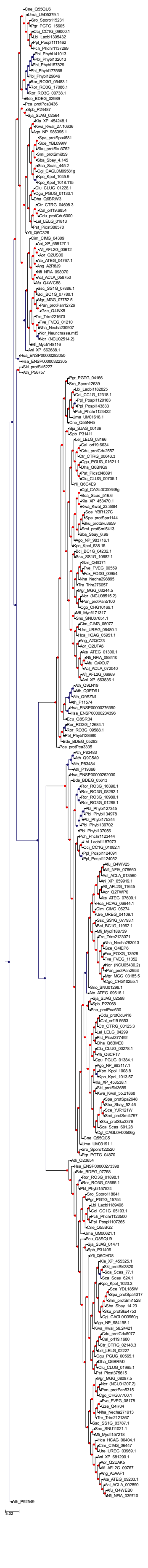

Complex V, F-type ATPase C (ATP9)

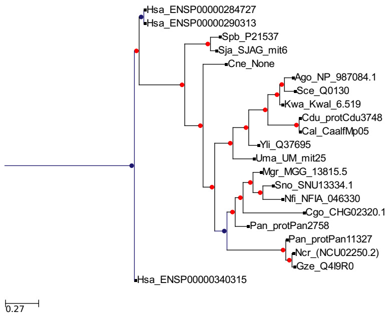

Complex V, F-type ATPase D

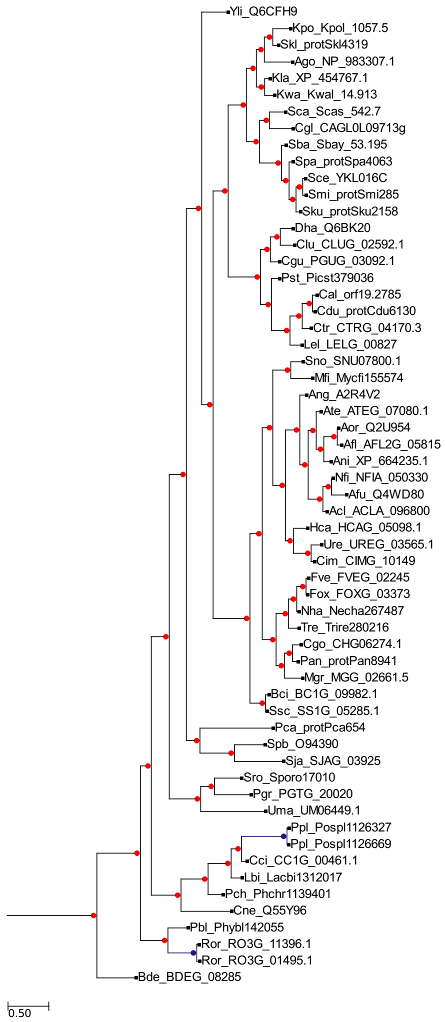

Complex V, F-type ATPase delta

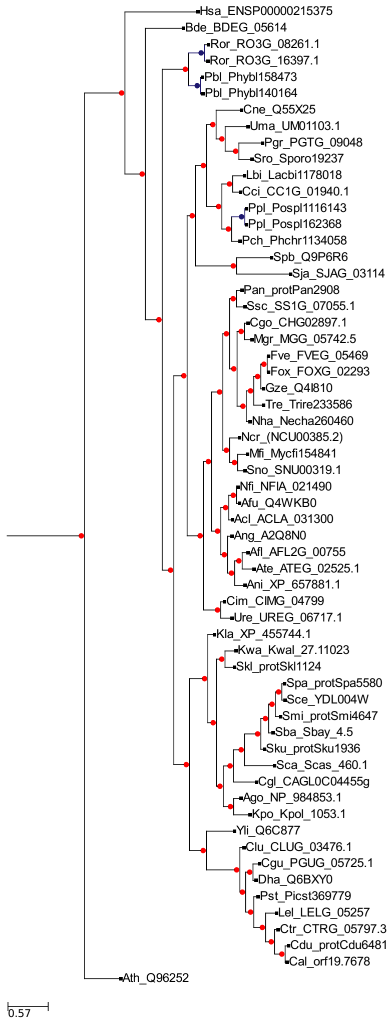

Complex V, F-type ATPase epsilon

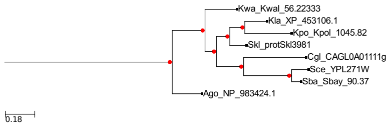

Complex V, F-type ATPase f

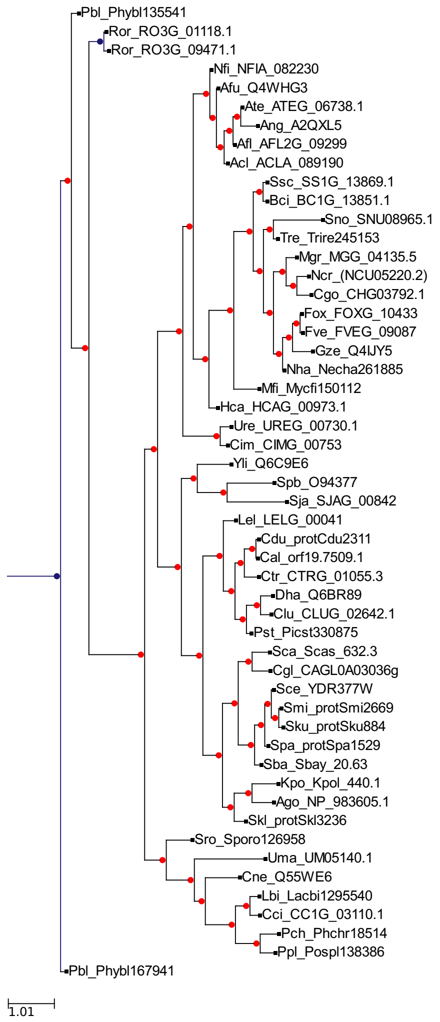

Complex V, F-type ATPase G

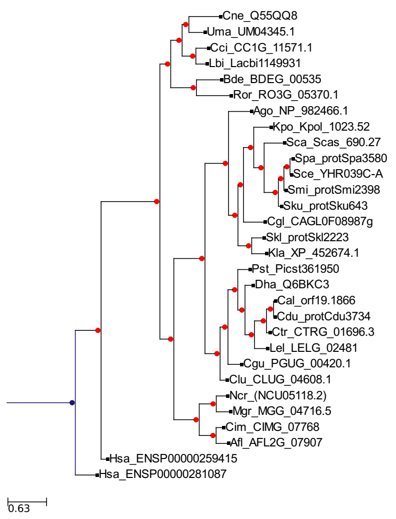

Complex V, F-type ATPase gamma

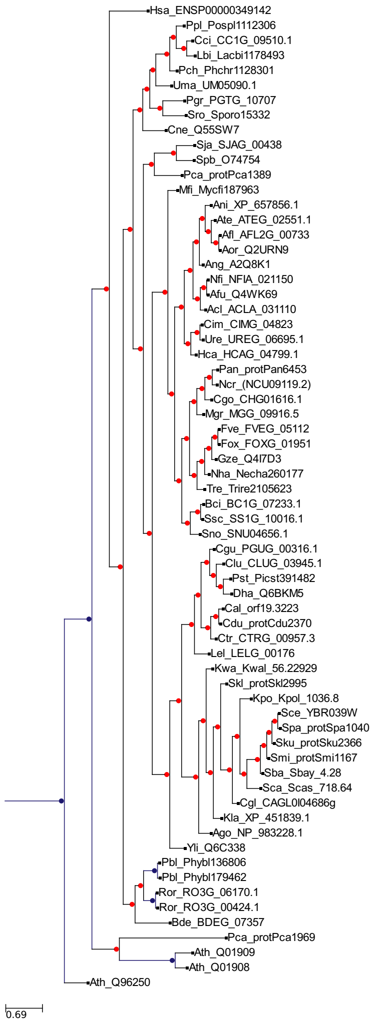

Complex V, F-type ATPase H

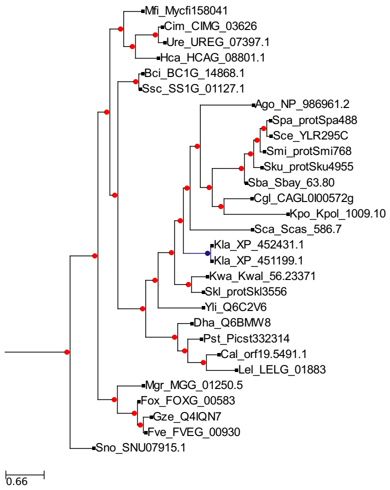

Complex V, F-type ATPase J

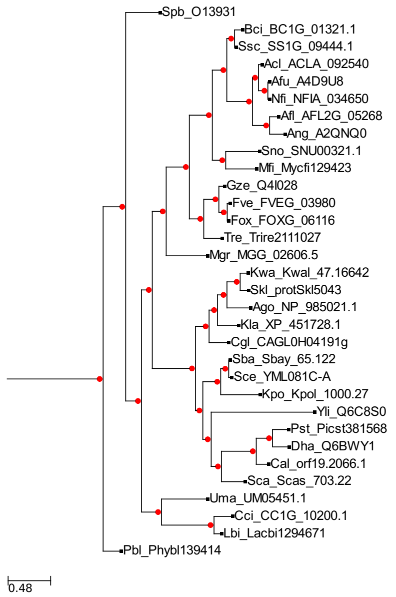

Complex V, F-type ATPase K

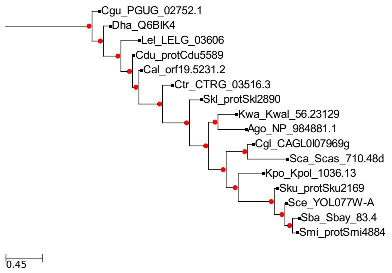

Complex V, F-type ATPase OSCP

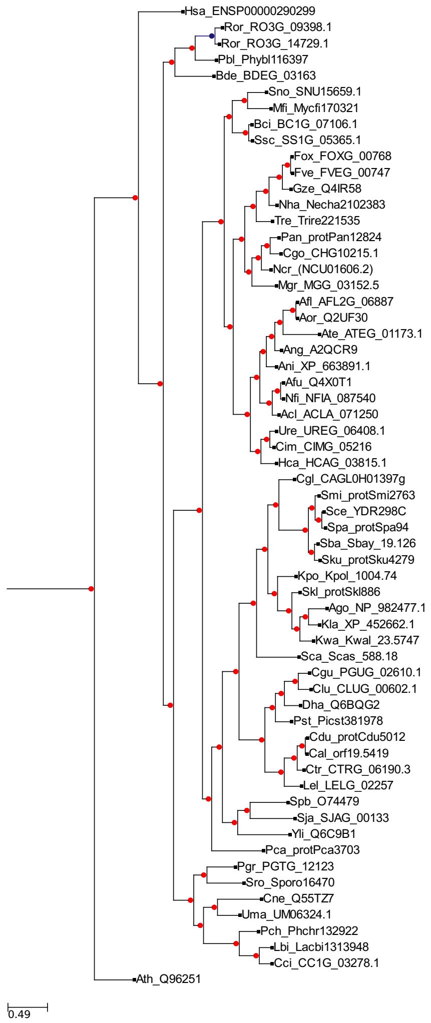

Alternative NADH

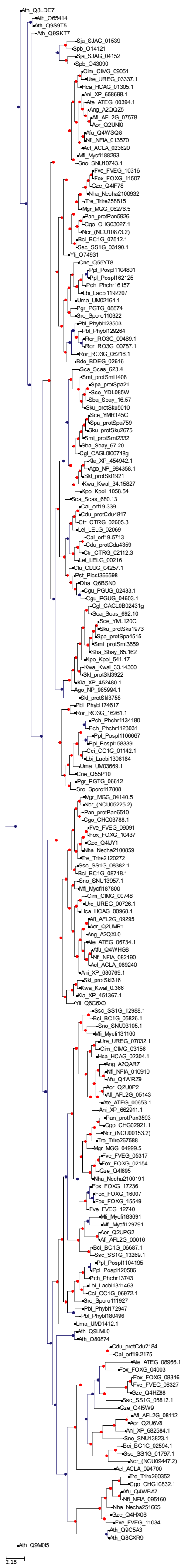

Alternative oxidase

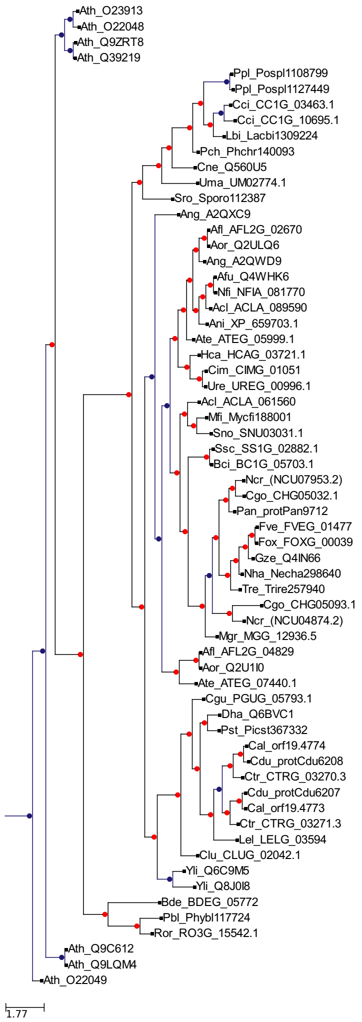

Supplement: Additional file 1 — Additional Material. Additional figures and tables cited in the text. [file 1471-2148-9-295-S1.PDF]
